# The Histone Variant MacroH2A1 Impacts Circadian Gene Expression and Cell Phenotype in an In Vitro Model of Hepatocellular Carcinoma

**DOI:** 10.3390/biomedicines9081057

**Published:** 2021-08-20

**Authors:** Annalucia Carbone, Elisabetta De Santis, Olga Cela, Vincenzo Giambra, Luca Miele, Giuseppe Marrone, Antonio Grieco, Marcus Buschbeck, Nazzareno Capitanio, Tommaso Mazza, Gianluigi Mazzoccoli

**Affiliations:** 1 Department of Medical Sciences, Division of Internal Medicine and Chronobiology Laboratory, Fondazione IRCCS Casa Sollievo della Sofferenza, 71013 San Giovanni Rotondo, Italy; annalucia.carbone@gmail.com; 2 Institute for Stem Cell Biology, Regenerative Medicine and Innovative Therapies (ISBReMIT), Fondazione IRCCS Casa Sollievo della Sofferenza, 71013 San Giovanni Rotondo, Italy; e.desantis@operapadrepio.it (E.D.S.); v.giambra@operapadrepio.it (V.G.); 3 Department of Clinical and Experimental Medicine, University of Foggia, 71100 Foggia, Italy; olga.cela@unifg.it (O.C.); nazzareno.capitanio@unifg.it (N.C.); 4 Fondazione Policlinico Universitario A. Gemelli-IRCCS, Catholic University of the Sacred Heart, 00168 Rome, Italy; luca.miele@policlinicogemelli.it (L.M.); giuseppe.marrone@policlinicogemelli.it (G.M.); antonio.grieco@unicatt.it (A.G.); 5 Josep Carreras Leukaemia Research Institute, IJC Building, Can Ruti Campus Ctra de Can Ruti, Camí de les Escoles s/n, 08916 Badalona, Spain; mbuschbeck@carrerasresearch.org; 6 Bioinformatics Unit, Fondazione IRCCS Casa Sollievo della Sofferenza, 71013 San Giovanni Rotondo, Italy; t.mazza@css-mendel.it

**Keywords:** circadian, cancer, biological clock, HCC, PER1

## Abstract

Hepatocellular carcinoma (HCC) is a leading cause of cancer-related death worldwide. A foremost risk factor for HCC is obesity/metabolic syndrome-related non-alcoholic fatty liver disease (NAFLD) and non-alcoholic steatohepatitis (NASH), which is prompted by remarkable changes in transcription patterns of genes enriching metabolic, immune/inflammatory, and circadian pathways. Epigenetic mechanisms play a role in NAFLD-associated HCC, and macroH2A1, a variant of histone H2A, is involved in the pathogenesis modulating the expression of oncogenes and/or tumor suppressor genes and interacting with SIRT1, which crucially impacts the circadian clock circuitry. Hence, we aimed to appraise if and how macroH2A1 regulated the expression patterns of circadian genes in the setting of NAFLD-associated HCC. We took advantage of an in vitro model of liver cancer represented by HepG2 (human hepatocarcinoma) cells stably knocked down for macroH2A1 and conducted whole transcriptome profiling and deep phenotyping analysis. We found up-regulation of PER1 along with several deregulated circadian genes, enriching several important pathways and functions related to cancer onset and progression, such as epithelial-to-mesenchymal transition, cell cycle deregulation, and DNA damage. PER1 silencing partially mitigated the malignant phenotype induced by the loss of macroH2A1 in HCC cells. In conclusion, our findings suggest a modulatory role for the core circadian protein PER1 in liver carcinogenesis in the context of a lack of the macroH2A1 epigenetic and transcriptional landscape.

## 1. Introduction

Liver cancer, the most common form of which is hepatocellular carcinoma (HCC), is a leading cause of death worldwide [1,2]. Therapeutic strategies to treat advanced HCC provide only marginal effects [3]. Obesity/metabolic syndrome, currently a pandemic, is a risk factor mainly for HCC among different types of cancer. In fact, obese individuals with a high body mass index (BMI 35–40) had the greatest increase in cancer risk (the risk of HCC was increased by ~5 times) [4]. Obesity is accompanied in more than 90% of cases by non-alcoholic fatty liver disease (NAFLD). NAFLD is the result of an imbalance between the availability of lipids (deriving from the uptake of circulating lipids or from de-novo lipogenesis) and the elimination of lipids (through the oxidation of free fatty acids or the secretion of lipoproteins rich in triglycerides). Consequently, visible lipid droplets accumulate in the hepatocytes. The natural history of NAFLD in humans is not easy to establish since most studies are retrospective; however, there is strong and convincing evidence that patients affected by NAFLD have a higher incidence of HCC. Subjects with NAFLD progress to non-alcoholic steatohepatitis (NASH) in 10% of the cases and eventually to HCC [5].

The high amount of fat in the liver can induce the development of HCC through inflammation, activating specific signal pathways such as those of NF-kB, STAT3/5, JNK, and SIRT1, and consequently the expression of a set of growth factors and cytokines (IL-6, IL-10, TNFα, and lymphotoxins) [3,4]. Indeed, alterations in hepatocyte metabolism and proliferation during steatosis and HCC are triggered by dramatic changes in gene transcription patterns. Metabolic pathways, inflammatory processes, and immune response are rhythmically driven by the biological clocks, light-entrainable intracellular mechanisms endowed in all cells and tissues, capable of self-sustaining oscillation, and committed to driving behavioral and physiological rhythms in the organism through hierarchical networking [6]. These molecular clockworks comprise transcriptional/translational feedback loops operated by core circadian genes and proteins (Arntl, Clock, Per1-2, Cry1-2), which drive rhythmic expression changes in thousands of downstream genes enriching pathways that control critical cell functions [7,8,9].

The biological clock plays a critical role in NAFLD onset and progression to NASH and HCC, impacting basic cellular processes, such as autophagy, cell cycle, DNA damage response, xenobiotic detoxification, reactive oxygen species production, and inactivation [10,11]; however, the epigenetic mechanisms involved in HCC and associated with obesity/metabolic syndrome/NAFLD have not been investigated in detail. DNA is wrapped around histone octamer’s backbone to the chromatin structure. The packaging of chromatin in the nucleus is regulated at different levels to allow transcriptional plasticity. One of these levels is the replacement of canonical histones (H2A, H2B, H3, and H4) with histone variants [12,13].

A variant of histone H2A is represented by macroH2A1, which is encoded by H2AFY, a gene present in both sex chromosomes and autosomes. Chromatin domains containing the macroH2A1 variant can be hundreds of kilobases long and occupy approximately a quarter of the human genome [14,15,16]. MacroH2A1 is thought to act as a strong transcriptional modulator that can repress transcription and activate some genes in response to growth signals that are not yet well defined [17,18]. Mice genetically ablated for macroH2A1 are hallmarked by liver steatosis and glucose and lipid metabolism alterations related to the genetic background [19,20]. MacroH2A1 is therefore considered a new factor involved in the pathogenesis of NAFLD. Interestingly, wild-type mice fed a lipogenic diet poor in methyl groups (methionine/choline-deficient diet), which induces liver steatosis and inflammation, show an increase in the total liver content of macroH2A1 [21]. Hence, the lack of macroH2A1 associated with chromatin impacts the complex interaction between enzymes and signal cascades that regulate the intracellular lipid turnover of hepatocytes [20].

MacroH2A1 is present in two isoforms, macroH2A1.1 and macroH2A1.2, generated following alternative RNA splicing. The expression of the macroH2A1.1 and macroH2A1.2 isoforms is predictive of the onset of lung cancer, breast cancer, and gastrointestinal cancers identifying this histone as a valuable marker for risk stratification in cancer patients [22]. The macroH2A1.1 isoform is expressed at high levels in cells that undergo senescence, a debated anticancer mechanism, suggesting that macroH2A1.1 may be a useful biomarker of senescent tumor cells [13,18].

The regulation of the expression of oncogenes and/or tumor suppressors by macroH2A1 in hepatocytes could be particularly relevant for the development of HCC associated with metabolic syndrome since the activity of these genes often mechanically connects NAFLD to the onset of HCC; therefore, macroH2A1 isoforms, in addition to being proposed as markers for lung, breast, and skin cancer, could play a key role in the pathogenesis of liver cancer as well [22,23,24]. As observed in macroH2A1 KO mice, NAFLD is associated with the development of a spectrum of liver damage, including carcinogenesis, and steatosis-associated HCC is histologically featured by immunopositivity for histone macroH2A1 isoforms marks [25]. Importantly, histone macroH2A1 deletion in HCC cells sets out CD4(+)CD25(+)FoxP3(+) regulatory T cells activation and upholds paracrine-mediated chemoresistance [26].

MacroH2A1.1 binds a small metabolite called O-acetyl-ADP-ribose (OAADPR) to high affinity, which macroH2A1.2 does not bind. OAADPR is generated by an enzymatic reaction catalyzed by SIRT1, a nicotinamide-adenine-dinucleotide (NAD)-dependent histone and protein deacetylase, whose activation is considered protective against cell metabolic and oxidative stress, along with being considered protective against aging [27]. SIRT1 is frequently found over-expressed in HCC and in this cancer type preserves liver cancer stem cells and their tumorigenic and self-renewal features, propping up tumor recurrence, metastatic spreading, and resistance to therapy [28,29]. Interestingly, liver-specific SIRT1 transgenic mice are protected against the development of HCC associated with metabolic syndrome [30], and SIRT1 is crucial for the functioning of the biological clock [31,32,33]. On these premises, and considering that macroH2A1 is a remarkable transcriptional modifier, we aimed to evaluate if and how this histone variant affected the expression pattern of circadian genes in the setting of HCC. We addressed this issue, taking advantage of an in vitro model of malignant hepatic tumor represented by HepG2 (human hepatocarcinoma) cells stably knocked down for macroH2A1 through short hairpin RNA and analyzed by RNA-Seq for whole transcriptome profiling and by deep cellular phenotyping. Several circadian genes were found deregulated, in particular, the core clock gene PER1 was up-regulated, with enrichment of several important pathways and functions related to carcinogenesis, such as epithelial-to-mesenchymal transition (EMT), altered cell cycle control, and DNA damage. PER1 silencing to some extent ameliorated HCC cell phenotype in the epigenetic and transcriptional context spawned by loss of macroH2A1 histone variant.

## 2. Materials and Methods

### 2.1. Primary Datasets

Bioinformatics analyses were performed on two publicly available genomic data: GSE117459 and GSE11923. The GSE117459 dataset renders the RNA-Seq profiling of the whole transcriptome of HepG2 cells knocked down for macroH2A1 [24]. Among the gene symbols in the dataset, we pointed out the genes included in the GSE11923 dataset, rendering high-temporal resolution profiling of C57BL/6J mouse liver samples through Affymetrix arrays. Circadian (24 ± 4 h) transcripts were categorized using Fisher’s G-test at a false-discovery rate of <0.05 and COSOPT [34].

Array probe IDs/nucleotide sequences of 24-h oscillating genes were listed using BioDBnet (https://biodbnet-abcc.ncifcrf.gov/db/db2db.php, accessed on 4 June 2021) and 1842 Ensembl Transcript IDs were recovered from the latter dataset.

### 2.2. Cell Culture

The HepG2 parental cell line, acquired from the American Type Culture Collection (ATCC, Rockville, MD, USA) and the HepG2 cell line with macroH2A1 knock out (KO) were cultured in Dulbecco’s Modified Eagle Medium (DMEM) high glucose (Thermo Fisher Scientific, Waltham, MA, USA) supplemented with 10% fetal bovine serum (FBS) (Thermo Fisher Scientific, Waltham, MA, USA) and 1% penicillin/streptomycin (P/S) (Thermo Fisher Scientific, Waltham, MA, USA). Cell cultures were grown in a 5% CO_2_ incubator at 37 °C.

### 2.3. Generation of Lentiviral Vectors

As previously described, lentiviral production and transduction of HepG2 cells were performed to stably suppress macroH2A1 expression through shRNAs [23,24,26].

### 2.4. RNA-Seq

For RNA-Seq, total RNA was extracted from control and macroH2A1 KD HepG2 cell lines with TRIzol Reagent (Thermo Fisher Scientific, Waltham, MA, USA). Indexed libraries were prepared from 2 mg/ea purified RNA with the TruSeq Total Stranded RNA Sample Prep Kit (Illumina, Cambridge, UK) according to the manufacturer’s instructions. Libraries were quantified using the Agilent 2100 Bioanalyzer (Agilent Technologies, Santa Clara, CA, USA) and pooled so that each index-tagged sample was present in equimolar amounts; the final concentration of the pooled samples was 2 nmol/L. Pooled samples were then subjected to cluster generation and sequencing using an Illumina HiSeq 2500 System (Illumina, Cambridge, UK) in a 2 × 100 paired-end format at a final concentration of 8 pmol/L. Short reads were aligned against the hg19 genome assembly using STAR (ver. 2.5.1a). Piled-up reads were counted with the htseq-count tool. Normalization of reads counts and their comparisons were performed using the EdgeR-based R pipeline. Regarding the assessment of differentially expressed circadian genes for macroH2A1 knockout (KO) versus control (CTL) cells, statistically significant differences between groups in gene expression were considered using a cut-off of >±1.5 for the fold change and <0.05 for the adjusted *p*-value ≤ 0.05. 

### 2.5. Analysis for Functional Categorization of the Most Significant Pathways Enriched by Deregulated Circadian Genes

Functional and pathway analyses were conducted using Ingenuity Pathway Analysis (IPA; QIAGEN, Redwood City, CA, USA; www.qiagen.com/ingenuity accessed on 4 June 2021) and specifically considering 1842 Ensembl Transcript IDs recovered from the GSE117459 dataset rendering the RNA-Seq profiling of the whole transcriptome of HepG2 cells knocked down for macroH2A1 and categorized as circadian (oscillating with 24 ± 4 h periodicity) using Fisher’s G-test at a false-discovery rate of <0.05 and COSOPT [34]. Array probe IDs/nucleotide sequences of 24 h oscillating genes were listed using BioDBnet (https://biodbnet-abcc.ncifcrf.gov/db/db2db.php accessed on 4 June 2021). Both enrichment analyses were based on the prior calculation of the activation z-scores, which infer the activation states of predicted biological diseases/functions and pathways. An enrichment score (Fisher’s exact test, *p*-value) was calculated to measure the overlap between observed and predicted regulated gene sets. We considered *p*-values < 0.05, z-scores > 1 (minimum activation threshold), and z-scores < −1 (minimum inhibition threshold) as significant.

### 2.6. Knockdown of PER1 by RNA Interference

Short interference RNA (siRNA) against PER1 was synthesized by (Sigma-Aldrich, St. Louis, MO, USA). The sequences of the sense and anti-sense PER1 siRNA were 5′-r (CUUUCCAACAGAUCUGUAA) d (TT)-3′ and 5′-r (UUACAGAUCUGUUGGAAAG) d (TT)-3′, respectively. The negative control (scrambled) siRNA sequences were as follows: 5′-r (UUCUCCGAACGUGUCACGU) d (TT)-3′ and 5′-r (ACGUGACACGUUCGGAGAA) d (TT)-3′. For the siRNA transfection experiments, control and macroH2A1 KD HepG2 cell lines were seeded in 6-well plates (5 × 10^5^ cells per well). After 24 h, cells were transiently transfected with the PER1-specific siRNA diluted in Opti-MEM (Thermo Fisher Scientific, Waltham, MA, USA) using Lipofectamine™ RNAiMAX Transfection Reagent (Thermo Fisher Scientific, Waltham, MA, USA) according to the manufacturer’s protocol. After transfection, the cells were incubated for 72 h and subjected to various analyses.

### 2.7. Western Blotting 

Cells transfected with siRNA were lysed using RIPA buffer supplemented with phosphatase and protease inhibitors (Roche, Basel, Switzerland). The protein amount was determined using a Pierce BCA Protein Assay kit (Thermo Fisher Scientific, Waltham, MA, USA). The obtained proteins were subjected to SDS–PAGE and then transferred to PVDF membranes, that were incubated overnight (ON) at 4 °C with specific antibodies against: PER1 (1:500), PER2 (1:500), Timeless (1:2000), TIPIN (1:1000), and BNIP3 (1:500), which were purchased from Abcam (Cambridge, UK); CRY1 (1:1000), CRY2 (1:1000), which were purchased from NOVUS (Centennial, CO, USA); ARNTL1 (1:1000), CLOCK (1:1000), SirT1 (1:1000), WEE1 (1:1000), c-MYC (1:1000), CDH1 (1:1000), ZEB1 (1:1000), LC3B (1:1000), Atg7 (1:1000), ULK1 (1:1000), and Beclin-1 (1:1000), which were purchased from Cell Signaling (Beverly, MA, USA); GABARAPL1 (1:1000) (Thermo Fisher Scientific, Waltham, MA, USA); C/EBPβ (1:500), β-Actin (1:1000), which were purchased from Santa Cruz Biotechnology (Dallas, TX, USA); p53 (1:1000) (Dako Agilent Pathology Solutions, Carpinteria, CA, USA). Membranes were then incubated for 1 h at RT with goat anti-mouse IgG or goat anti-rabbit IgG secondary antibodies (BIO-RAD, Hercules, CA, USA). Proteins were revealed with Pierce ECL Western Blotting Substrate (Thermo Fisher Scientific, Waltham, MA, USA) and images were acquired using ChemiDoc™ Imaging system (BIO-RAD, Hercules, CA, USA). Protein levels were quantified using the Image Lab software (version 2.1.0.35.deb) (BIO-RAD, Hercules, CA, USA) and normalized to actin.

### 2.8. Cell Proliferation Assay 

To evaluate the proliferation ability, HepG2 cells, control, and macroH2A1 KD, transfected with negative control and PER1 siRNA, were seeded into 96-well plates in 90 μL complete DMEM medium, at a density of 1 × 10^4^ cells/well, and incubated overnight at 37 °C in 5% CO_2_ atmosphere to enable cell adhesion. Cell number and density of viable cells were determined at 0, 24, 48, 72, and 96 h using PrestoBlue™ Cell Viability assay (Thermo Fisher Scientific, Waltham, MA, USA) according to the manufacturer’s instructions. Two biological replicates were prepared, and each condition was assayed in triplicate; the results are expressed as mean ± standard deviation (SD).

### 2.9. Cell Migration Assay 

Migration was assessed using an in vitro scratch wound healing assay. HepG2 cells with the different conditions were seeded in a 6-well plate at a density of 1 × 10^6^ cells/well for 24 h to achieve 100% confluence followed by starvation in serum-free DMEM medium for 2 h to completely inhibit cell proliferation. After generating a linear scratch wound in the cell monolayer with a sterile 200 µL pipette tip, the culture medium containing detached cells and cellular debris was removed by washing and replaced with a fresh medium. The scratched area was captured at 0 and 72 h after scratching using an Olympus Inverted Phase Contrast Microscope with a 10× phase objective. The width of the gap between the invasion fronts was measured using Fiji software (ImageJ) to calculate the rate of wound closure. The wound closure values refer to the initial position. All experiments were performed at least two times in triplicates.

### 2.10. Flow Cytometry Analysis 

#### 2.10.1. Cell Cycle 

To analyze cell cycle phase distribution, 10^5^/mL cells were cultured as previously described in wells of a 12-multiwell plate for each condition to evaluate the incorporation of BrdU after 2 h of in vitro growth. The incorporation of BrdU was carried out with 10 µL of BrdU 10 mg/mL by using APC BrdU Flow Kit (Becton Dickinson, BD, Franklin Lakes, NJ, USA). Subsequently, treated cells were fixed, permeabilized, and treated with DNase to expose incorporated BrdU. Cells were then stained with APC-conjugated anti-BrdU antibody and resuspended in a 7-AAD solution (1×). All FACS data were acquired on FACS Calibur, Canto2 (Becton Dickinson, BD, Franklin Lakes, NJ, USA) and analyzed using FlowJo software (Becton Dickinson, BD, Franklin Lakes, NJ, USA).

#### 2.10.2. Half Maximal Inhibitory Concentration (IC50) of Cisplatin

To determine cisplatin’s IC50, 10^5^ cells/500 uL were cultured as previously described in 24-multiwell plate and incubated in controlled atmosphere (37 °C, 5% O_2_, 5% CO_2_, RH > 95%) for 72 h. Cisplatin (Sigma-Aldrich, S. Louis, MO, USA) tested concentrations were 0.1, 0.5, 1, 5, 10, and 50 μmol/L. After 72 h of in vitro growth, cells were detached and washed once in PBS1X 3% FBS. The DAPI fluorescent DNA dye (1:1000 dilution; Sigma-Aldrich) was used to identify live cells.

#### 2.10.3. DNA Damage and Apoptosis Assay with and without Cisplatin

Cells for each condition were treated in vitro with 1 μmol/L cisplatin for 72 h to assess DNA damage. Subsequently, treated cells and negative controls were fixed in PFA 4% for 15 min at RT and then permeabilized in ice-cold 90% methanol for 10 min in ice. Cells were washed once in BD PERM/WASH buffer 1× (Becton Dickinson, BD, Franklin Lakes, NJ, USA) to remove methanol and then resuspended in 100 µL of 1:100 diluted Anti-Phospho-Histone H2AX (Ser139) (Cell Signaling, Beverly, MA, USA). After 1 h of primary antibody incubation, cells were stained for 30 min with AlexaFluor 488 conjugated secondary antibody (Thermo Fisher Scientific, Waltham, MA, USA). Cells were then washed once in BD PERM/WASH buffer 1X and resuspended in 400 µL of BD PERM/WASH buffer 1X for flow cytometric analysis. To assess apoptosis, cisplatin-treated cells as reported above and controls were stained following the kit protocol PE Annexin V Apoptosis Detection Kit I (RUO) (Becton Dickinson, BD, Franklin Lakes, NJ, USA).

#### 2.10.4. Mitochondria and Cellular Respiration Measurements 

Oxygen consumption rates (OCR) were assessed in intact HepG2 cells by Clark electrode (Oxygraph, Hansatech Instruments Ltd., Narborough Rd, Pentney, King’s Lynn PE32 1JL, UK). Briefly, 2–3 × 10^6^ cells were suspended in 0.5 mL of DMEM, and the attained endogenous basal OCR was followed for 5 min; after that, 1.0 µg/mL of the FoF1 ATP-synthase inhibitor oligomycin (OCROligom.) was added, followed by the addition of 1.0 μmol/L of the uncoupler valinomycin (OCRValinom.). Finally, 2 μmol/L antimycin A + 2 μmol/L rotenone (inhibitors of the mitochondrial respiratory chain complex III and complex I, respectively) were added and the attained residual OCR subtracted from the previously recorded OCRs to estimate the mitochondrial-dependent respiratory activity expressed as nmol of O_2_ consumed/min/10^6^ cells. The OCRValinom. is the maximal respiratory capacity; the difference between basal-OCR and OCROligom. is a measure of the ATP-generating respiration; the OCROligom./basal-OCR ratio is a measure of the proton leak (i.e., the capacity of the mitochondrial inner membrane to dissipate the respiration-mediated electrochemical proton gradient). Where indicated, 4 µM etomoxir, an inhibitor of carnitinepalmitoyltransferase 1a (CPT-1a), was present in the medium from the beginning of the assay.

#### 2.10.5. Statistical Analysis

Data are shown as mean ± standard error of the mean (SEM). Group comparison was assessed by one-way ANOVA and Tukey’s honestly significant difference test, using R (R Core Team, 2020). *p*-values were adjusted using the Benjamini–Hochberg procedure. Statistical significance was declared if *p*-value  ≤  0.05.

## 3. Results

### 3.1. Loss of MacroH2A1 Histone Variants Changes the Expression of Circadian Genes in HCC Cells

**Figure 1:**
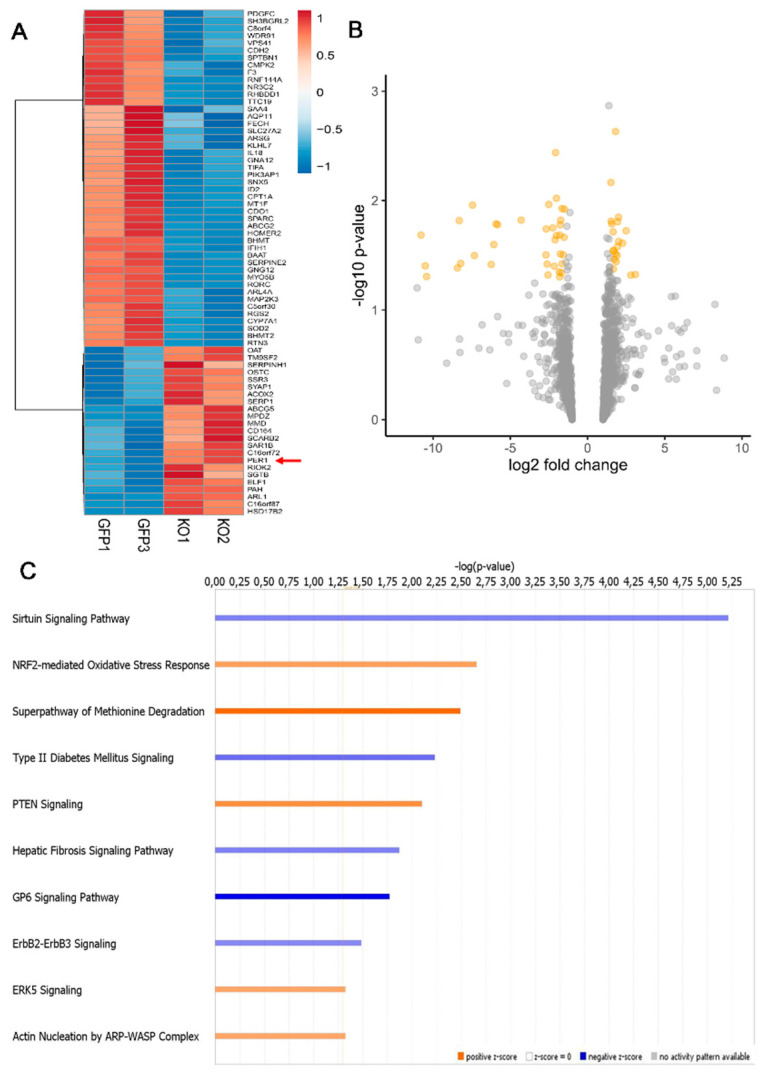
Histone variant macroH2A1 knockdown affects the expression level of circadian genes. (**A**) Heat-map rendering mRNA expression levels of 24 h oscillating genes upon histone variant macroH2A1 knock down in HepG2 cells. GFP1 and GFP3 indicate HepG2 control cells, KO1 and KO2 indicate macroH2A1 knocked-down HepG2 cells (biological replicates). (**B**) Volcano plot of differentially expressed circadian genes between HepG2 KD and HepG2 control cells. Indicated in orange are the genes with statistically significant different expression, i.e., *p*-value < 0.05 and abs (FC) > 2. (**C**) Enrichment analysis for functional categorization of the most significant pathways. Functional and pathway analyses were conducted using Ingenuity Pathway Analysis (IPA; QIAGEN, Redwood City, CA, USA; www.qiagen.com/ingenuity accessed on 4 June 2021).

Using a cut-off of >±1.5 for the fold change and <0.05 for the adjusted *p*-value, the assessment of differentially expressed circadian genes for macroH2A1 knockout (KO) versus control (CTL) cells showed a slight transcriptional divergence between the matched HepG2 cell lines with 69 differentially expressed genes, 24 up-regulated and 45 down-regulated (Figure 1, Supplementary Table S1).

Among the most up-regulated genes, HSD17B2 (fold change 263.67) encodes 17β-hydroxysteroid dehydrogenase type 2, an enzyme crucial for steroid hormones metabolism and for intratumoral estrogens and androgens metabolism (intracrinology) impacting tumor progression and patient’s prognosis in sex-steroid-dependent cancers as well as in estrogen and androgen receptor-positive liver cancer [35,36,37,38,39]. MPDZ (fold change 76.83) encodes phosphatidylinositol 3-monophosphate (PtdIns3P)-binding protein that dynamically handles key intracellular signaling and operates intracellular second messengers engaged in numerous cellular processes such as cell proliferation, migration, and survival [40]. Deregulation of PI3K-dependent pathways is straightforwardly engaged in carcinogenesis through abnormal activation of the PI3K/AKT/mTORC1 axis [41] and when highly expressed at the protein level in HCC tissue associates with a more favorable prognosis in patients affected by liver cancer [40,42]. MPDZ modulates DLL4-induced Notch signaling in the vasculature through physical interaction with DLL1 and DLL4, assisting their interaction with the adherents junction protein Nectin-2 and improving Notch signaling activity with tumor angiogenesis drop off [43].

The protein encoded by ABCG5 (fold change 16.35) is one of the ATP-binding cassette (ABC) transporters superfamily members, involved in extra and intracellular membrane transport of different molecules and, in particular, of neutral sterol in hepatobiliary and transintestinal cholesterol excretion and its inactivating mutations cause a rare genetic disorder, Sitosterolemia [44]. SGTB (fold change 3.09) encodes small glutamine-rich tetratricopeptide repeat containing beta, an isoform of SGT, a co-chaperone of heat shock cognate protein of 70 kDa (Hsc70), and its down-regulation by miR-365b enhances HCC cell migration and invasion in vitro and low SGTB tissue expression was found correlated with more favorable HCC progression and prognosis [45,46]. The protein-coding gene SERPINH1 (fold change 2.80) encodes a member of the serine protease inhibitor superfamily. This serpine is co-expressed with SNHG6 (small nucleolar RNA host gene 6) through the miRNA-139-5p interplay and enhances the in vitro malignant phenotype of HCC cell lines (HepG2, Hep3b, HLE, and Huh-7) [47]. The most down-regulated gene, GNG12 (fold change −115.27), encodes guanine nucleotide-binding protein subunit gamma-12, a G protein family member entailed in the modulation of the inflammatory signaling cascade, which when highly expressed in tumor tissue, enhances cancer cell growth and predicts poor prognosis in pancreatic cancer [48] as well as in liver cancer, in this case in cooperation with GNA12 (fold change −1.79) [42]. Another highly down-regulated gene, CDO1 (FC −60.54), encodes cysteine dioxygenase type 1 and is a tumor suppressor found silenced by promoter methylation in different cancer types, such as non-small cell lung cancer and gastrointestinal cancers, including liver cancer [49,50,51,52]. SPARC (secreted protein acidic and rich in cysteine, fold change −36.12) encodes a glycoprotein involved in extracellular matrix remodeling and influencing cell growth and hepatic fibrogenesis in chronic inflammation as well as cell proliferation, migration and invasion, angiogenesis, and tumor progression in HCC, where its decreased expression in tumor tissue predicts poorer prognosis [53,54,55]. CYP7A1 (fold change −31.38) encodes a member of the cytochrome P450 superfamily of rate-limiting monooxygenases engaged in drug metabolism, lipids/steroids/cholesterol synthesis, and cholesterol to bile acids conversion. Interestingly, bile acids homeostasis disorders along with augmented hepatic bile acids toxic levels associates with higher HCC incidence [56]. RHBDD1 (fold change −29.99) encodes an intramembrane-cleaving serine protease that, similar to other rhomboid proteins, is involved in human cancer progression with a growth-promoting role negatively impacting patients’ survival in different cancer types (e.g., colorectal cancer and breast cancer) [57,58,59].

Among the core clock genes encoding the circadian proteins that hardwire the molecular clockwork, we found up-regulation of PER1 (period circadian regulator 1, fold change 1.5), which is a candidate tumor suppressor gene and, when highly expressed in tumor tissue, is a favorable prognostic biomarker in liver cancer [42,60]. PER1 drives a key connection amid the molecular clockwork, the cell cycle machinery, and the DNA damage response (DDR) cooperating with ATM and Chk2 checkpoint proteins [61,62]. When over-expressed, PER1 was shown to set off apoptosis associated with DNA damage and altered expression of critical cell cycle controllers, such as WEE-1, Cyclin B1, and CDC2 in various human breast (MDAMB-231), colon (SW48), lung (NCI-H460), and endometrial (Ishikawa) cancer cell lines [61,62].

### 3.2. Circadian Genes Deregulated upon MacroH2A1 Knockout Enrich Important Pathways in HCC Cells

Performing functional and pathway analyses with IPA exclusively on 1842 circadian genes sorted out from the GSE117459 dataset (see Materials and Methods), we found significant enrichment in seven down-regulated pathways (Sirtuin signaling pathway, hepatic fibrosis signaling pathway, autophagy, GP6 signaling pathway, ErbB2-ErbB3 signaling, stearate biosynthesis I, fatty acid β-oxidation I, type 2 diabetes mellitus signaling), and three up-regulated pathways (NRF2-mediated oxidative stress response, PTEN signaling, superpathway of methionine degradation). (Figure 1, Supplementary Table S2).

Furthermore, we found significantly decreased activation for several functions related to carcinogenesis in the hepatobiliary and gastrointestinal system as well as lipid molecular transport and lipid and steroid synthesis/metabolism. On the contrary, we found increased activation of functions related to cancer aggressiveness and progression, such as epithelial-mesenchymal transition, cell cycle (interphase), DNA fragmentation, and recombination (Supplementary Table S3).

Among the significantly down-regulated pathways, the sirtuin signaling cascade is particularly relevant, which interplays both with macroH2A1 epigenetic regulation and circadian clock circuitry functioning. In the liver, SIRT1 mainly controls glucose and lipid metabolism, fine-tuning insulin sensitivity, gluconeogenesis/glycolysis, and fatty acid oxidation/cholesterol metabolism. SIRT1 operates as an intracellular metabolic sensor and, through deacetylation and activation of PGC1-α and PPARα, enhances glucose homeostasis and fatty acids β-oxidation [63]. Further, adjusting the transcriptional activity of CLOCK/BMAL1 heterodimer according to nutrient sensing through intracellular NAD+ levels, SIRT1 takes part in the functioning of the biological clock, and deacetylating PER2 regulates the expression of oscillating tissue-specific genes, joining cellular metabolism to the molecular clockwork [64].

### 3.3. MacroH2A1 Histone Knockdown in HCC Alters the Expression of Proteins Entailed in Key Cell Functions

**Figure 2:**
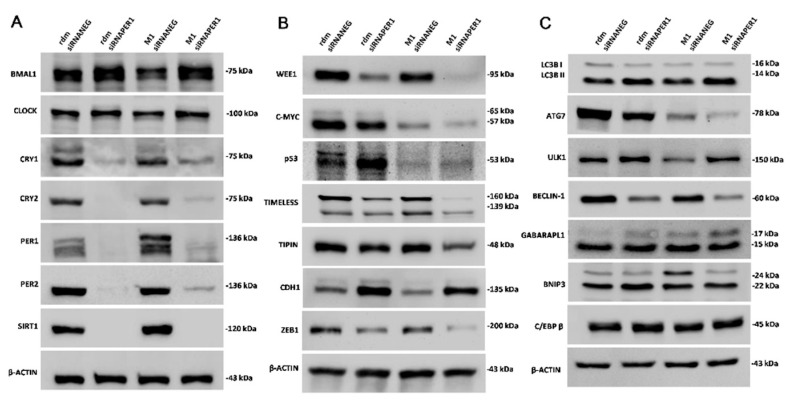
Western blot analysis of the expression levels of circadian proteins and regulatory proteins and transcription factors involved in key cell processes in the considered cell conditions. (**A**) Western blot analysis of the expression levels of proteins involved in the molecular clockwork. (**B**) Western blot analysis of the expression levels of proteins involved in cell processes. (**C**) Western blot analysis of the expression levels of proteins involved in the autophagic process. See the Materials and Methods section for more details. RDM siRNA NEG = siRNA scramble-treated HepG2 cells; RDM siRNA PER1 = siRNA PER1-treated HepG2 cells; M1 siRNA NEG = siRNA scramble-treated macroH2A1 knocked down HepG2 cells; M1 siRNA PER1 = siRNA PER1-treated macroH2A1 knocked down HepG2 cells; bars, standard error of mean (SEM); three biological replicates were each assayed in triplicate and results were expressed as mean ± standard error of mean (SEM).

Loss of macroH2A1 histone variants determines variations in the expression of different circadian proteins and silencing of PER1 seems to unveil that its deregulation may account, at least in part, for these alterations (Figure 2). In particular, the core transcription factor BMAL1 is increased in macroH2A1 KO cells and further increases upon PER1 silencing, while the expression of CLOCK does not seem to be influenced by the knockdown of macroH2A1 and subsequently of PER1. According to transcriptomics data, the expression of PER1 is increased following knockdown of the macroH2A1 histone variants, and its silencing is accompanied by a striking reduction in the expression of CRY1, CRY2, and PER2, as well as SIRT1, at the protein level. The reduction in the expression of the circadian proteins operating the negative arm of the transcriptional–translational feedback hardwiring of the biological clock could be related to the extremely low level of expression of SIRT1, which plays a fundamental role in the correct functioning of the circadian transcriptional process [65]. Knockdown of macroH2A1 histone decreases WEE-1 and, even more, c-MYC protein expression, with further reduction following PER1 silencing. These two proteins link the biological clock to cell cycle control (WEE-1 blocking G2/M transition and c-MYC upholding G0/G1 transition) and their alteration, in the context of macroH2A1 KO-related epigenetic landscape, should be deciphered considering other protein changes to explain the greater replication of macroH2A1 KO cells, in particular in comparison to cells with PER1 silencing [66]. The biological clock intervenes in the coordination of cell cycle progression, DNA replication, and DDR through CRY2 and the interaction of TIMELESS/TIPIN with the fork protection complex on the one hand and with the cell cycle checkpoint proteins Chk1 and the ATR-ATRIP complex on the other hand to organize mitotic kinase activation with DNA replication termination and DNA damage checkpoint response to protect fork integrity and genome stability [67,68,69]. Protein levels of TIMELESS were slightly increased upon knockdown for macroH2A1 histone, contributing probably to lower DNA damage-induced apoptosis upon cisplatin challenge, an effect that is reverted by PER1 silencing. Opposite patterns were shown for the p53 protein, which is greatly decreased in macroH2A1 KO cells, with partial rescue upon PER1 silencing, and could greatly contribute to the phenotypic changes observed in these cells, particularly regarding the cell cycle and apoptosis. P53 represses the transcription of PER1 and, for its part, PER1, through physical interaction, reduces the stability and transcriptional activity of p53 [70].

The biological clock also drives the circadian oscillation of the proteins involved in the autophagy process by controlling the oscillation of the transcription factors C/EBPβ, an important transactivator of autophagy proteins [71], as well as TFEB/TFE3, driving autophagy in relation to nutrient sensing and metabolism [72]. Interestingly, among the evaluated proteins, ULK1 and GABARAPL1 were previously described to oscillate with the circadian pattern, while the expression of ATG7 and BECLIN-1 was not rhythmic [71].

In our hands, some autophagy proteins, such as GABARPL1 and BNIP3, were increased, while LC3B, Beclin-1, and in particular ULK and ATG7, were decreased upon knockdown of macroH2A1 histone, with further reduction upon PER1 silencing. These latter protein changes suggest that the two different autophagic pathways, the conventional Atg5-dependent pathway and the alternative Atg5-independent pathway, are restrained. The interpretation of this pattern is difficult, considering that autophagy is a double-edged sword in carcinogenesis, on one side providing tumor suppression by restraining cancer-cell survival and inducing cell death, on the other side supporting the proliferation of cancer cells, aiding them in enduring the hostile tumor microenvironment and improving growth and aggressiveness [73]. The enhancement of malignant phenotype observed in macroH2A1 knocked down cells could also be related to a decrease in E-cadherin (epithelial, CDH1) in the presence of a high level of the transcription factor ZEB1 (Zinc Finger E-Box Binding Homeobox 1), which hallmarks the activation of the epithelial-to-mesenchymal transition (EMT) program, a long-lasting and reversible process involved in cancer progression and featured by loss of cell polarity and cell–cell adhesion, achievement of migratory and invasive features together with stem cell properties and reduced sensitivity to chemotherapy [74]. Interestingly, upon PER1 silencing, CDH1 increased and ZEB1 decreased in macroH2A1 knocked down cells, suggesting reasonable EMT revert and hinting that this core clock protein plays a key role in determining cell phenotype and fate in the epigenetic background triggered by knockdown of macroH2A1 histone.

### 3.4. Knockdown of MacroH2A1 Histone Modulates Proliferative and Migratory Capacity of HCC Cells

**Figure 3:**
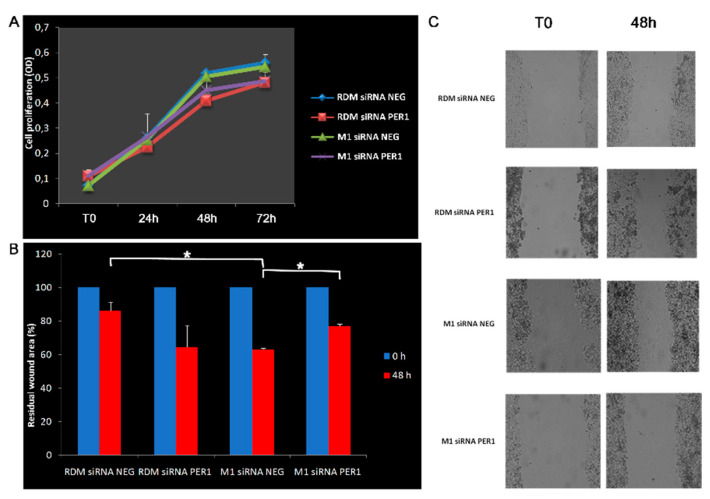
Evaluation of proliferative and migratory capacity: (**A**) cell proliferation curves; (**B**) wound area relative to closure (%) assessed at 48 h. Artificial wounds were made in confluent monolayers of control and macroH2A1 KO with or without PER1-silencing cells. Cell migration towards the wound, photographed from 0 h to 48 h. (**C**) Representative microphotographs of the residual wound area. Original magnification 10X. Cell migration was determined by the rate of scratched area closure over time using ImageJ™ software. See the Materials and Methods section for more details. RDM siRNA NEG = siRNA scramble-treated HepG2 cells; RDM siRNA PER1 = siRNA PER1-treated HepG2 cells; M1 siRNAneg = siRNA scramble-treated macroH2A1 knocked down HepG2 cells; M1 siRNA PER1 = siRNA PER1-treated macroH2A1 knocked down HepG2 cells; bars, standard error of mean (SEM); * *p* < 0.05; three biological replicates were each assayed in triplicate and results were expressed as mean ± standard error of mean (SEM).

The evaluation of cell proliferation did not reveal significant differences between control cells; however, cells knocked down for macroH2A1 histone variants show slower proliferation at 48 and 72 h. Following PER1 silencing, these cells seem to acquire greater proliferative capacity, although not statistically significant. As regards the ability to migrate, the picture seems to be the opposite, as following knockdown of macroH2A1 histone variants, the cells show greater ability to migrate when evaluated as a percentage of wound area closure after 48 h (*p*-value < 0.05), while upon silencing of PER1 this ability to migrate tends to decrease in the cells knocked down for macroH2A1 histone (*p*-value < 0.05) and to increase, albeit not significantly, in control cells. This pattern could be related to the macroH2A1 histone-linked epigenetic landscape and the biological clock’s ability to control the expression of metalloproteinases, which play a crucial role in inflammatory processes, extracellular matrix homeostasis, wound healing, and cancer cell migration [75,76] (Figure 3).

### 3.5. MacroH2A1 Histone Loss Impacts Cell Cycle, Anticancer Drug Sensitivity, DNA Damage/Apoptosis in HCC Cells

**Figure 4:**
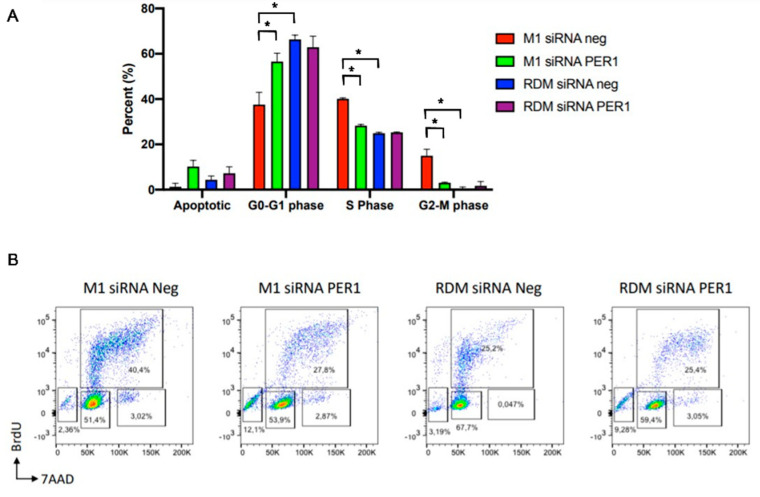
Cell cycle phase distribution analysis in HepG2 cells upon macroH2A1 knockdown and PER1 silencing. (**A**) The percentage of cells at G0/G1-, S-, and G2/M-phases is plotted. (**B**) Flow cytometry graphs showing the distribution of cycling cells by FACS analysis. See the Materials and Methods section for more details. RDM siRNA NEG = siRNA scramble-treated HepG2 cells; RDM siRNA PER1 = siRNA PER1-treated HepG2 cells; M1 siRNAneg = siRNA scramble-treated macroH2A1 knocked down HepG2 cells; M1 siRNA PER1 = siRNA PER1-treated macroH2A1 knocked down HepG2 cells; bars, standard error of mean (SEM); * *p* < 0.05; three biological replicates were each assayed in triplicate and results were expressed as mean ± standard error of mean (SEM).

The analysis of the results obtained with the evaluation of the cell cycle in flow cytometry shows a greater replicative capacity of the HepG2 macroH2A1 KO cells compared to the other cell line conditions and, in particular, the PER1 down-regulated cells and the control HepG2 cells, a difference that results in a greater number of cells in the S and G2-M phase (*p*-value < 0.05) and in fewer cells in the quiescent state referred to as G0 (*p*-value < 0.05) (Figure 4). These results corroborate the significant role played by the biological clock and PER1 in particular in the control of cell cycle progression interplaying with WEE-1 and c-MYC, as well as with p53, capable of setting off G1 cell cycle arrest through c-MYC transrepression and cell cycle arrest genes transactivation [61,77,78,79]. 

**Figure 5:**
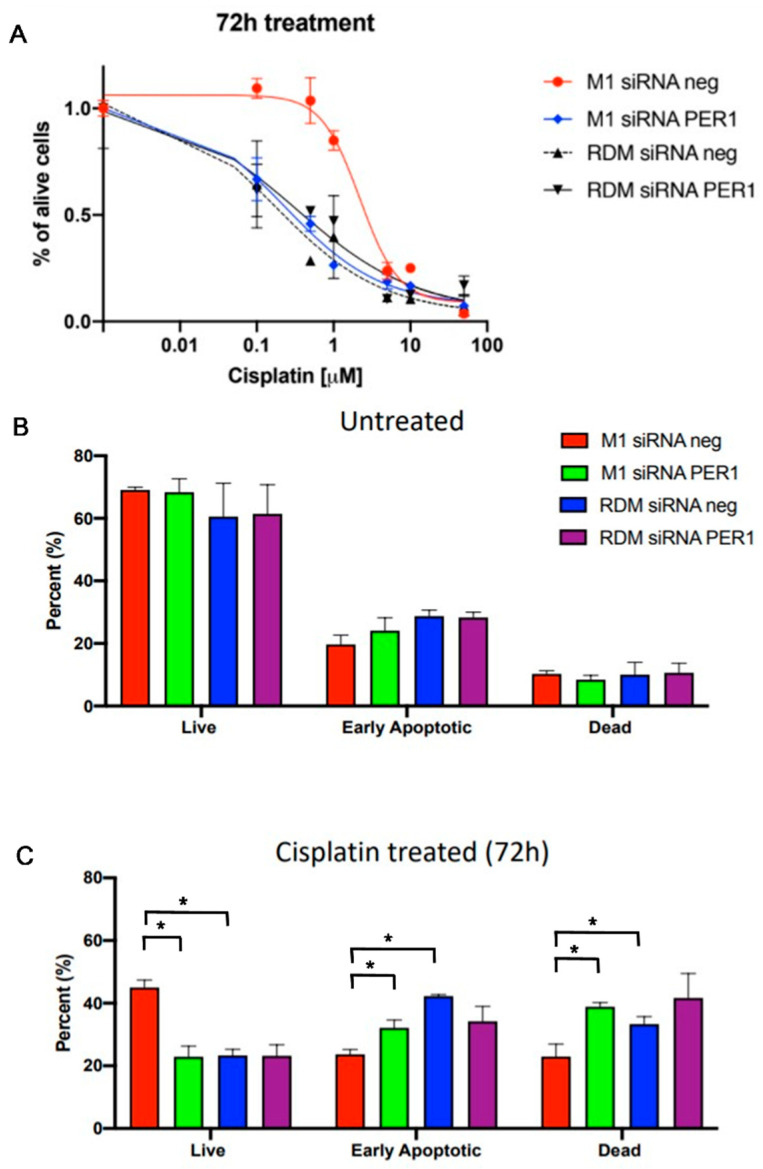
Evaluation of apoptosis changes upon macroH2A1 knockdown and PER1 silencing in basal conditions and upon cisplatin treatment. (**A**) Plot of the concentration–response sigmoidal curve using IC50 values to parameterize concentration–effect curves. (**B**) Apoptosis changes upon macroH2A1 knockdown and PER1 silencing in basal conditions; (**C**) Apoptosis changes upon macroH2A1 knockdown and PER1 silencing with cisplatin treatment. RDM siRNA NEG = siRNA scramble-treated HepG2 cells; RDM siRNA PER1 = siRNA PER1-treated HepG2 cells; M1 siRNAneg = siRNA scramble-treated macroH2A1 knocked down HepG2 cells; M1 siRNA PER1 = siRNA PER1-treated macroH2A1 knocked down HepG2 cells; bars, standard error of mean (SEM); * *p* < 0.05; three biological replicates were each assayed in triplicate and results were expressed as mean ± standard error of mean (SEM).

After 72 h of treatment with cisplatin at dosing scalars ranging from 0.1 to 100 μmol/L, the HepG2 macroH2A1 KO cells showed reduced sensitivity to the chemotherapeutic agent compared to the other cell line conditions (*p*-value < 0.05), supporting the importance of PER1 and the circadian clock circuitry in modulating cell response to chemotherapeutic agents [79] (Figure 5).

**Figure 6:**
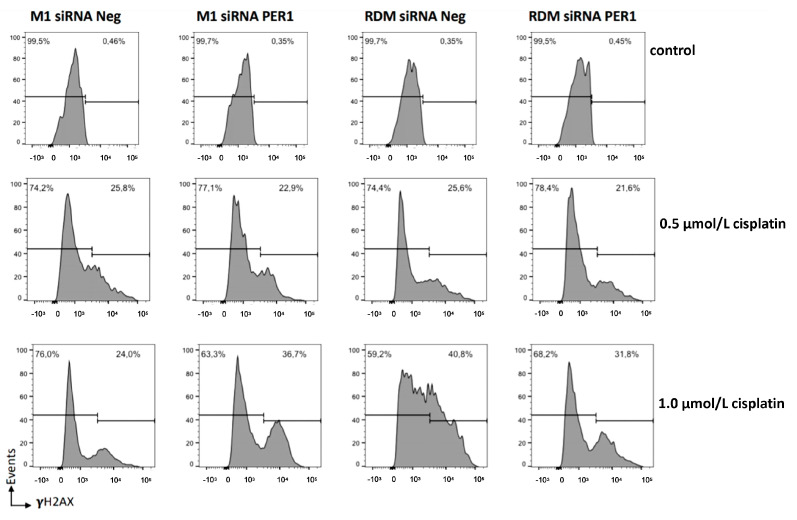
Flow cytometry detection of DNA damage. To evaluate DNA damage, we used the H2A.X phosphorylation assay, a cell-based assay using flow cytometry detection of levels of phosphorylated histone H2A.X on serine 139 (γ-H2AX), formed in response to DNA double-strand breaks (DSBs) generated by exogenous genotoxic agents. Cells for each condition were treated in vitro with 0, 0.5, and 1 μmol/L cisplatin for 72 h. See the Materials and Methods section for more details. RDM siRNA NEG = siRNA scramble-treated HepG2 cells; RDM siRNA PER1 = siRNA PER1-treated HepG2 cells; M1 siRNAneg = siRNA scramble-treated macroH2A1 knocked down HepG2 cells; M1 siRNA PER1 = siRNA PER1-treated macroH2A1 knocked down HepG2 cells; three biological replicates were each assayed in triplicate.

The nucleosomal histone protein H2AX is specifically phosphorylated at serine 139 (γ-H2AX) adjacent to DSBs and measuring γH2AX as a genotoxicity endpoint; there were no significant differences in DNA damage evaluated in the absence of anticancer drug treatment, while after treatment with cisplatin at a dosage of 1 μmol/L there was less evidence of DNA damage in the macroH2A1 KO cells compared to the PER1 down-regulated cells (*p*-value < 0.05) and the other cell line conditions, in particular, the control HepG2 cells, which showed higher levels of cisplatin-induced DNA damage, partly mitigated by PER1 silencing (*p*-value < 0.05) (Figure 6). These results corroborate the contrasting role PER1 and the molecular clockwork played in the DNA damage response [61,80], in this case, influenced by the loss of macroH2A1-related epigenetic background.

In basal conditions, there were no significant differences in the number of apoptotic cells, while following challenge with chemotherapy treatment represented by 1 μmol/L cisplatin dosing the HepG2 macroH2A1 KO cell type showed a greater number of viable cells (*p*-value < 0.05) and less presence of apoptotic cells when compared to the other cell line conditions and, in particular, the PER1 down-regulated cells (*p*-value < 0.05) and the control HepG2 cells (*p*-value < 0.05). These results are in agreement with previous studies showing that PER1 decreases the sensitivity of cancer cells to drug-induced apoptosis [70,81,82].

### 3.6. Loss of MacroH2A1 Decreases Mitochondrial Oxidative Phosphorylation Efficiency

**Figure 7:**
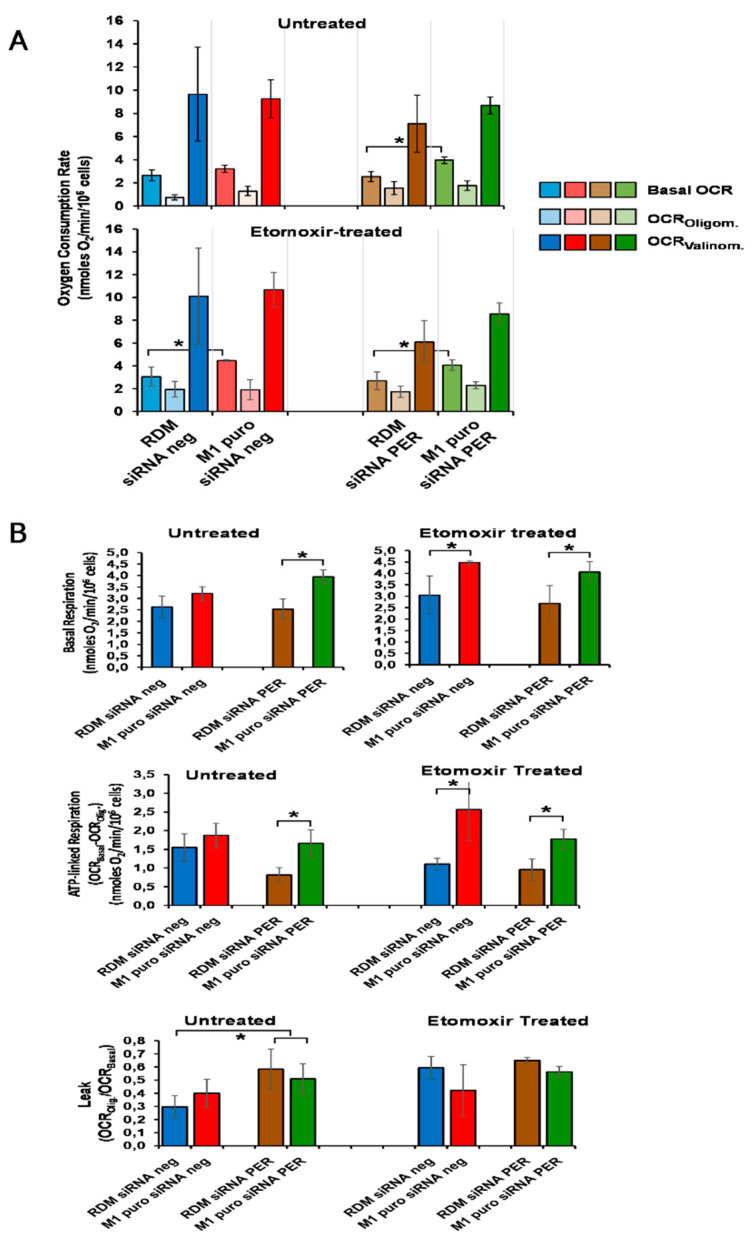
Effect of macroH2A1 and PER1 modulation on mitochondrial respiratory activity in HepG2 cells. Intact cells were assessed by high-resolution oximetry. (**A**) Oxygen consumption rates (OCR) outcomes. The upper and lower panel refer to OCRs measured in the absence and in the presence of etomoxir, respectively. The colored bars refer, with different tones, to basal OCR and following the addition of oligomycin and valinomycin (see legend). The values are corrected for mitochondria-unrelated respiration (expressed as nmoles O_2_/min/10^6^ cells). (**B**) Mitochondrial respiration-linked bioenergetics parameters inferred from the results in (**A**). Upper bipartite panels: basal respiration on an enlarged scale; middle panel: ATP-linked respiration attained subtracting from the basal OCRs the OCROligom; lower panels: normalized respiratory leak attained as the OCROligom/OCRBasal ratio. See the Materials and Methods section for more details. RDM siRNA NEG = siRNA scramble-treated HepG2 cells; RDM siRNA PER1 = siRNA PER1-treated HepG2 cells; M1 siRNA NEG = siRNA scramble-treated macroH2A1 knocked down HepG2 cells; M1 siRNA PER1 = siRNA PER1-treated macroH2A1 knocked down HepG2 cells; bars, standard error of mean (SEM); * *p* < 0.05; three biological replicates were each assayed in triplicate and results were expressed as mean ± standard error of mean (SEM).

Mitochondrial respiration in cancer has been for a long time erroneously underestimated because of a misunderstanding of Warburg’s effect [83]. Indeed, nowadays, the importance of mitochondrial oxidative metabolism has been fully re-evaluated in cancer development, maintenance, metastasis, and chemoresistance [84,85]. Moreover, mitochondrial oxidative phosphorylation is emerging to interplay with the circadian clockwork [86,87,88,89], as well as with epigenetic modifications [90,91]. On this basis, we assessed mitochondrial respiration in the context of macroH2A1 KO and/or PER1 silencing in HepG2 cells and upon treatment with the fatty acid oxidation inhibitor etomoxir. No significant effect of macroH2A1 KO and PER1 silencing was observed on basal oxygen consumption rate (basal OCR); however, in PER1 silenced HepG2 cells, part of the basal OCR was uncoupled from ATP synthesis, likely owing to increased passive dissipation of the proton motive force as indicated by enhanced proton leak. Interestingly, in macroH2A1 KO cells, PER1 silencing recovered ATP-linked respiration by increasing basal OCR. When mitochondrial respiration was assessed in the presence of etomoxir, a condition inhibiting long-chain fatty acid β-oxidation, macroH2A1 KO significantly stimulated both basal OCR and ATP-linked OCR irrespective of co-silencing of PER1 (Figure 7). The absence of significant changes in the fully uncoupled respiration (i.e., OCRValinom) in the macroH2A1 KO and/or PER1 silenced cells, either with or without etomoxir treatment, would rule out substantial variations in the content of the respiratory chain components, on the other hand suggesting modulation of the metabolic fluxes upstream of them and bringing on, among the functions found up-regulated in macroH2A1 KO cells, the up-regulation of oxidative stress response pathway mediated by NRF2, directly involved in cellular and mitochondrial bioenergetics regulation though management of metabolites propped up for mitochondrial respiration [92]. These results would indicate that macroH2A1 exerts a depressive effect on mitochondrial oxidative phosphorylation efficiency, which is more evident under metabolic stress conditions. Speculatively, this would prevent an adaptive shift away from glycolysis, a metabolic setting hallmarking cancer cells. Activation/expression of NRF2, a key driver of anti-inflammatory, antioxidant, and cytoprotective responses, transcriptionally impacts the expression of genes managing cellular protection from oxidative stress and inflammation, both biological clock-controlled processes [11] and is involved in the pathogenic mechanisms underlining onset and progression of liver diseases, including NAFLD/NASH and HCC.

## 4. Discussion

Histone modifications participate in epigenetic mechanisms regulating transcription, DNA replication, and damage repair, as well as mitotic chromosome segregation during carcinogenesis [93]. The expression level of H2AFY associated with prognosis in HCC patients [94] and macroH2A1 deletion has been shown to impact the phenotype of HCC cells, eliciting reduced proliferation rate, chemotherapy resistance, and stem-like metabolic rewiring (increased hypoxic responses and glycolysis), leading them to acquire cancer stem cell features, among which the capability to give rise to significantly larger and less differentiated tumors when injected into nude mice [24]. On the other side, emerging evidence shows that deregulation of circadian genes is closely associated with hepatic carcinogenesis and disruption of circadian rhythms, which are driven by the molecular clockwork, and take part in HCC promotion and progression [95,96,97,98,99,100,101,102,103]. We evaluated the expression of circadian genes sorted in the whole transcriptome of HepG2 cells knocked down for macroH2A1 and profiled through RNA-Seq [24]. 

Concerning the control HepG2 cells, the mRNA expression level of 69 circadian genes was deregulated, with 24 up-regulated genes and 45 down-regulated genes in macroH2A1 KO cells (Figure 1). These genes significantly enriched several remarkable pathways, of which some were down-regulated (Sirtuin signaling pathway, hepatic fibrosis signaling pathway, autophagy, GP6 signaling pathway, ErbB2-ErbB3 signaling, stearate biosynthesis I, fatty acid β-oxidation I, type 2 diabetes mellitus signaling), and the others up-regulated (NRF2-mediated oxidative stress response, PTEN signaling, and superpathway of methionine degradation). In addition, we found significantly decreased activation of several functions related to carcinogenesis in the hepato–biliary system and in several other different systems/tissues, lipid molecular transport, and lipid and steroid synthesis and metabolism. In contrast, EMT, cell cycle, DNA fragmentation, and recombination functions showed increased activation. Among the genes found up-regulated in macroH2A1 KO cells, PER1 encodes a core circadian protein of the molecular clockwork that takes part in several vital cellular processes, whether transcriptionally or by protein–protein interactions such as cell cycle and DNA damage response. PER1 blocks cyclin D1, necessary for cell cycle G1/S transition, and interacts with the checkpoint proteins to maintain checkpoint activation in the presence of DNA damage [104]. The biological clock drives the expression of WEE-1 kinase, which acts at the G2/M checkpoint, and is transcriptionally activated by BMAL1/CLOCK and repressed by PER1, which also inhibits the entry of cells from G1 into the S phase, likely through c-MYC stabilization [104]. In macroH2A1 KO cells, WEE1 protein levels were increased while c-MYC was decreased, so that the increased number of these cells in the G2/M and S phase could be related to changes of other molecular factors, mainly to a striking protein level decrease in p53, which induces cell-cycle arrest mostly through transcriptional activation of p21/WAF1 that in turn binds to cyclin E/Cdk2 and cyclin D/Cdk4 complexes to set out G1 arrest in the cell cycle [105]. In our hands, PER1 silencing smoothed back WEE1, c-MYC, and p53 protein changes and cell cycle patterns, suggesting a modulatory role of the core circadian protein in this interplay. Furthermore, PER1 upholds cell cycle arrest upon DNA damage through direct protein-protein interaction with the DSB-activated kinases ataxia telangiectasia mutated (ATM), which is responsible for phosphorylation of checkpoint kinase 2 (CHK2), and activation of p53 [106]. Interestingly, reduced levels of p53 in macroH2A1 KO cells co-occurred with increased levels of TIMELESS, suggesting that these cells could better resist induction of apoptosis and cope with replication stress-induced DNA damage. Moreover, autophagic proteins were decreased in macroH2A1 KO cells and further diminished upon PER1 silencing, hinting that autophagy contribution does not appear critical for cell survival upon loss of macroH2A1. Further, the unbalanced expression of E-cadherin (cell adhesion molecule) and ZEB1 (transcription factor involved in epithelial marker genes repression and mesenchymal marker genes activation) found in macroH2A1 KO cells, with partial regress upon PER1 silencing, pinpoints EMT as a further mechanism driving the malignant phenotype of HCC cells upon macroH2A1 knockdown.

We have to acknowledge some limitations in our study. Above all, there was no prior research on the specific topic we aimed to address, i.e., the changes in the expression of circadian genes with the epigenetic background determined by a lack of macroH2A1 histone variants. We could not rely on pre-existing data, and we planned an exploratory study to obtain preliminary results with the macroH2A1 KO-related epigenetic landscape and circadian genes modulation. The main limitation is that the study was entirely based on analyzing a single HCC cell line; thus, we advise a cautious interpretation of the findings. In this study, we lay the groundwork for a more complete research study in the future.

## 5. Conclusions

Cell cycle deregulation, replication stress-related DNA damage with DNA synthesis slow down/replication fork stalling, and genomic instability over and above autophagy and EMT are the main biological mechanisms sustaining carcinogenesis and metastatic spreading. Loss of macroH2A1 prompts phenotypic changes of HCC cells with the acquirement of stem-like features and chemotherapy resistance. Here, we show that the loss of macroH2A1 in HCC cells induces deregulation of several circadian genes, where the up-regulation of PER1, a core clock gene, stands out. In our study, PER1 silencing smoothed back changes of fundamental molecular factors controlling the cell cycle, apoptosis, DDR, EMT, and autophagy, involved in cancer onset and development. On this premise, a candidate tumor suppressor role for PER1 in liver cancer should be carefully evaluated in the context of a lack of macroH2A1 histones, but at the same time could represent a potential molecular target with implications for therapeutic strategies. 

## Data Availability

Not applicable.

## Supplementary Materials

The following are available online at https://www.mdpi.com/article/10.3390/biomedicines9081057/s1. Supplementary Tables S1–S3.
