# Supplementary material for: The Histone Variant MacroH2A1 Impacts Circadian Gene Expression and Cell Phenotype in an In Vitro Model of Hepatocellular Carcinoma
Source: Biomedicines. 2021 Aug 20;9(8):1057. doi: 10.3390/biomedicines9081057 (PMC8391426; doi:10.3390/biomedicines9081057)
Supplement: Supplementary file 1 [file biomedicines-09-01057-s001.zip › biomedicines-1343511 -supplementary.pdf]

Table S1. Differentially expressed circadian genes sorted in the whole transcriptome of HepG2 cells knocked-down for macroH2A1 and profiled through RNA-Seq in comparison with control HepG2 cells			
Gene ID	Gene description	*p*-value	Fold change
HSD17B2	*hydroxysteroid (17-beta) dehydrogenase 2*	0.033298	263.6635
MPDZ	*multiple PDZ domain crumbs cell polarity complex component*	0.046816	76.82502
ABCG5	*ATP-binding cassette. sub-family g (white). member 5*	0.040097	16.35242
SGTB	*small glutamine-rich tetratricopeptide repeat (tpr)-containing. beta*	0.047386	3.093502
SERPINH1	*serine (or cysteine) peptidase inhibitor. clade H. member 1*	0.047949	2.80151
ACOX2	*acyl-coenzyme a oxidase 2. branched chain*	0.018885	2.498472
CD164	*sialomucin core protein 24 (endolyn)*	0.024452	2.268357
OAT	*ornithine aminotransferase*	0.023627	2.018097
SYAP1	*synapse associated protein 1*	0.014185	2.008324
C16orf87	*chromosome 16 open reading frame 87*	0.015596	1.947027
ELF1	*e74-like factor 1*	0.026288	1.941783
MMD	*monocyte to macrophage differentiation-associated*	0.031814	1.913883
SCARB2	*scavenger receptor class B. member 2*	0.036201	1.849744
PAH	*phenylalanine hydroxylase*	0.030445	1.816262
ARL1	*ADP-ribosylation factor-like 1*	0.002337	1.81595
SAR1B	*SAR1 gene homolog b (s. cerevisiae)*	0.034401	1.722179
SERP1	*stress-associated endoplasmic reticulum protein 1*	0.028741	1.687226
OSTC	*oligosaccharyltransferase complex subunit*	0.042151	1.667932
TM9SF2	*transmembrane 9 superfamily member 2*	0.028584	1.664967
SSR3	*signal sequence receptor. gamma*	0.019285	1.65734
RIOK2	*rio kinase 2 (yeast)*	0.016325	1.567359
C16orf72	*chromosome 16 open reading frame 72*	0.015424	1.527632
PER1	*period homolog 1 (drosophila)*	0.006832	1.51221
RTN3	*reticulon 3*	0.011982	-1.50179
KLHL7	*kelch-like 7 (drosophila)*	0.021659	-1.51883
ARL4A	*ADP-ribosylation factor-like 4a*	0.031309	-1.57754
VPS41	*vacuolar protein sorting 41 (yeast)*	0.037859	-1.64827
WDR91	*WD repeat domain 91*	0.011823	-1.65977
AQP11	*aquaporin 11*	0.045677	-1.70335
TIFA	*traf-interacting protein with forkhead-associated domain*	0.015102	-1.73665
TTC19	*tetratricopeptide repeat domain 19*	0.030689	-1.76779
MYO5B	*myosin Vb*	0.01665	-1.77951
GNA12	*guanine nucleotide binding protein. alpha 12*	0.020672	-1.788
FECH	*ferrochelatase*	0.04973	-1.80066
SNX5	*sorting nexin 5*	0.044838	-1.83988
C5orf30	*chromosome 5 open reading frame 30 (MACIR - macrophage immunometabolism regulator)*	0.009526	-2.01614
IFIH1	*interferon induced with helicase c domain 1*	0.020856	-2.01981
SPTBN1	*spectrin beta. non-erythrocytic 1*	0.003645	-2.07228

PDGFC	*platelet-derived growth factor. c polypeptide*	0.02281	-2.1033
MAP2K3	*mitogen-activated protein kinase kinase 3*	0.039758	-2.15536
RORC	*rar-related orphan receptor gamma*	0.017741	-2.2504
BAAT	*bile acid-coenzyme a: amino acid n-acyltransferase*	0.010839	-2.51111
SLC27A2	*solute carrier family 27 (fatty acid transporter). member 2*	0.047782	-2.56038
ID2	*inhibitor of dna binding 2*	0.038002	-2.61672
SOD2	*superoxide dismutase 2. mitochondrial*	0.018167	-2.67649
IL18	*interleukin 18*	0.031359	-2.69189
F3	*coagulation factor iii*	0.015085	-4.30378
SERPINE2	*serine (or cysteine) peptidase inhibitor. clade e. member 2*	0.016606	-5.82444
C8orf4	*chromosome 8 open reading frame 4 (TCIM - transcriptional and immune response regulator*	0.016299	-5.93032
ARSG	*arylsulfatase g*	0.025216	-6.0746
SH3BGRL2	*SH3 domain binding glutamic acid-rich protein like 2*	0.038313	-6.24096
PIK3AP1	*phosphoinositide-3-kinase adaptor protein 1*	0.031764	-7.32897
RGS2	*regulator of g-protein signaling 2*	0.011005	-7.45041
MT1F	*metallothionein 1f*	0.037504	-8.2128
CDH2	*cadherin 2*	0.015223	-8.30998
SAA4	*serum amyloid a 4*	0.041197	-8.42766
ABCG2	*ATP-binding cassette. sub-family g (white). member 2*	0.049408	-10.4263
CPT1A	*carnitine palmitoyltransferase 1a. liver*	0.03947	-10.5143
CMPK2	*cytidine monophosphate (ump-cmp) kinase 2. mitochondrial*	0.020668	-10.7883
BHMT2	*betaine-homocysteine methyltransferase 2*	0.011336	-13.1606
NR3C2	*nuclear receptor subfamily 3. group c. member 2*	0.027234	-13.7428
HOMER2	*homer homolog 2 (drosophila)*	0.048467	-21.43
BHMT	*betaine-homocysteine methyltransferase*	0.006191	-25.7045
RNF144A	*ring finger protein 144a*	0.040718	-27.3101
RHBDD1	*rhomboid domain containing 1*	0.042967	-29.9971
CYP7A1	*cytochrome p450. family 7. subfamily a. polypeptide1*	0.033018	-31.383
SPARC	*secreted acidic cysteine rich glycoprotein*	0.046717	-36.121
CDO1	*cysteine dioxygenase 1. cytosolic*	0.038785	-60.5354
GNG12	*guanine nucleotide binding protein (g protein). gamma 12*	0.000291	-115.271

Supplementary Table S2. Ingenuity Canonical Pathways significantly enriched			
Pathway	-log(p-value)	z-score	Molecules
Superpathway of Methionine Degradation	2.49E00	2.236	AHCY,BHMT,BHMT2,CDO1,MRM2
PTEN Signaling	2.1E00	1.667	FGFR2,FOXO3,GSK3A,HRAS,MAGI3,PDGFRB,PDPK1,PIK3CA,YWHAH
NRF2-mediated Oxidative Stress Response	2.66E00	1.414	CCT7,DNAJB1,DNAJC5,FMO1,GCLC,GSTM2,HRAS,KEAP1,MAP2K3,MAP3K7,PIK3CA,SOD2,STIP1
ERK5 Signaling	1.32E00	1.342	FOXO3,GNA12,HRAS,MEF2A,YWHAH
Actin Nucleation by ARP-WASP Complex	1.32E00	1.342	GNA12,HRAS,PPP1R12A,RHOD,ROCK1
Cholecystokinin/Gastrin-mediated Signaling	1.34E00	1.134	GNA12,HRAS,IL18,MAP2K3,MEF2A,RHOD,ROCK1
Heme Biosynthesis II	4.2E00	1	ALAS1,FECH,PPOX,UROD
Inhibition of ARE-Mediated mRNA Degradation Pathway	2.19E00	1	CNOT4,CNOT6,EXOSC2,MAP3K7,PPP2R1A,PRKAR2A,PSME2,PSMF1,YWHAH
IL-1 Signaling	1.41E00	1	GNA12,GNG12,MAP2K3,MAP3K7,NFKBIA,PRKAR2A
Fatty Acid Activation	3.48E00	-1	ACSL3,ACSL4,ACSL5,SLC27A2
Bile Acid Biosynthesis, Neutral Pathway	3.48E00	-1	AKR1D1,BAAT,CYP7A1,HSD3B7
PPAR Signaling	2.65E00	-1	CITED2,HRAS,IL18,MAP3K7,NCOA1,NFKBIA,NRIP1,PDGFC,PDGFRB
Senescence Pathway	2.4E00	-1	CAPN2,DMTF1,E2F5,EIF4EBP1,ELF1,FOXO3,HRAS,KAT2B,MAP2K3,MAP3K7,PIK3CA,PPP2R1A,SAA4,SMAD7,SOD2,YPEL3
Cardiac β-adrenergic Signaling	2.26E00	-1.134	AKAP11,AKAP9,GNG12,PDE2A,PDE9A,PKIG,PPP1R12A,PPP1R14B,PPP2R1A,PRKAR2A
Fatty Acid β-oxidation I	2.78E00	-1.342	ACSL3,ACSL4,ACSL5,AUH,SLC27A2
Intrinsic Prothrombin Activation Pathway	2.25E00	-1.342	COL5A3,F11,FGA,FGB,FGG
Stearate Biosynthesis I (Animals)	2.08E00	-1.342	ACSL3,ACSL4,ACSL5,ELOVL6,SLC27A2
ErbB2-ErbB3 Signaling	1.49E00	-1.342	ERBB3,GSK3A,HRAS,PDPK1,PIK3CA
Autophagy	2.87E00	-1.342	ATG3,ATG7,CTSH,MAP1LC3B,RB1CC1,ULK1,VPS11
Sirtuin Signaling Pathway	5.21E00	-1.414	ACLY,ACSS2,ATG3,ATG4D,CDH1,CLOCK,CPT1A,ESRRA,FOXO3,G6PD,GABARAP,GTF3C2,MAP1LC3B,NDUFA12,NDUFA9,NDUFB9,NDUFS8,NR1H4,PPIF,SIRT5,SOD2,TIMM10,TIMM44
Hepatic Fibrosis Signaling Pathway	1.87E00	-1.414	COL5A3,DVL1,HRAS,IL18,JAK2,KLF9,MAP2K3,MAP3K7,NFKBIA,PDGFC,PDGFRB,PIK3CA,PRKAR2A,RHOD,ROCK1,SMAD7,SOD2,WNT5B
Type II Diabetes Mellitus Signaling	2.23E00	-1.633	ACSL3,ACSL4,ACSL5,IRS1,MAP3K7,NFKBIA,PDPK1,PIK3CA,SLC27A2,SLC2A2
GP6 Signaling Pathway	1.77E00	-2.828	COL27A1,COL5A3,FGA,FGB,FGG,GSK3A,PDPK1,PIK3CA
An increase in the Z-score value indicates pathway activation, while the decrease in the Z-score indicates pathway inhibition. On a logarithmic scale (-log (p-value) a P-value equal to 0.05 corresponds to 1.3 and the statistical significance of the result increases with the decrease in p-value and with the increase in - log (p-value) values.			

Supplementary Table S3: Significantly deregulated functions

Categories	Function	Diseases Or Functions Annotation	P-Value	Z-Score	Molecules	
Cell Cycle	Interphase	Interphase	2.45E-04	2.587	ABCB1,AKAP9,BAP1,BID,CAMKK2,CDH1,CEBPD,CES1,CYLD,DCTN1,DGKZ,DMTF1,DPP4,E2F5,EGR1,EIF4EBP1,ERBB3,ESRRA,FOXO3,GNA12,HRAS,HSPB1,ID2,ID4,ING4,IRS1,JAK2,KITLG,KLF12,KPNB1,LMNA,LPIN1,MAP2K3,MRNIP,NET1,NFKBIA,NRIP1,OGA,POLE4,PPIF,PPM1A,PPP1R12A,PPP2R1A,PRPF19,PSEN2,RASSF3,RB1CC1,RPA1,SMAD7,SOD2,TCP1,TUBB	52
Lipid Metabolism, Small Molecule Biochemistry	Binding	Binding Of Lipid	1.02E-03	2.414	CPT1A,DNAJB1,F2R,FKBP4,IRS1,NR1H4,NR3C2,SIRT5,STIP1	9
DNA Replication, Recombination, And Repair	Degradation	Degradation Of DNA	2.41E-04	2.253	ABCB1,BIK,CAPN2,CD47,DHX9,ENDOG,HRAS,HSPB1,KEAP1,KITLG,KPNB1,NFKBIA,PIK3CA,PRDX6,SOD2,STK24,VPS13A	17
Cell Death And Survival,DNA Replication, Recombination, And Repair	Fragmentation	Fragmentation Of DNA	4.78E-04	2.174	ABCB1,BIK,CAPN2,ENDOG,HRAS,HSPB1,KEAP1,KITLG,KPNB1,NFKBIA,PIK3CA,PRDX6,SOD2,STK24,VPS13A	15
DNA Replication, Recombination, And Repair,Nucleic Acid Metabolism, Small Molecule Biochemistry	Hydrolysis	Hydrolysis Of Nucleotide	1.04E-04	1.873	ABCG2,ANXA7,ATP1A1,CCT4,CCT5,ITPA,N4BP2,NME1,PDE2A,PDE9A,RAN,SNRNP200,TBC1D16	13
Infectious Diseases	Infection	Infection Of Cells	1.20E-04	1.649	AFG3L2,ARGLU1,CAMKK2,CBLB,CD164,CDH1,CLDND1,CLOCK,CLTA,CXADR,CYB5B,DCTN1,DHX9,DNAJB1,DPP4,DVL1,F2R,F3,FAM172A,FAM76B,FPGS,GABARAP,GPD2,HMGCR,HRAS,IL18,KLHDC2,KPNB1,LIN7C,LSM14B,MAP3K7,MPDZ,MRPL44,NUP62,PER3,PNRC1,PRPF6,PSEN2,PSME2,RAB1B,RABEPK,RB1CC1,REPIN1,SBF2,SLC10A1,SLC20A1,SPTBN1,ST3GAL4,STARD3NL,STIP1	52

					,UBE2L3,UNG	
Infectious Diseases	Infection	Infection By HIV-1	5.78E-04	1.399	AFG3L2,ARGLU1,CAMKK2,CD164,CLDND1,CLOCK,CLTA,DHX9,DNAJB1,DPP4,DVL1,FAM172A,FAM76B,FPGS,GABARAP,GPD2,IL18,KLHDC2,KPNB1,LIN7C,LSM14B,MAP3K7,MPDZ,MRPL44,NUP62,PER3,PNRC1,PRPF6,PSEN2,PSME2,RAB1B,RABEPK,RB1CC1,REPIN1,SBF2,SPTBN1,STARD3NL,STIP1,UBE2L3,UNG	40
Cellular Development	Epithelial-Mesenchymal Transition	Epithelial-Mesenchymal Transition	9.41E-04	1.376	AHNAK,ARHGAP21,CDH1,DICER1,ERBB3,FGFR2,FOXO3,HOOK1,HRAS,IRS1,MACROH2A1,MEF2A,NCOA1,NFKBIA,PDLIM1,PDPK1,PIK3CA,PRKAR2A,RICTOR,SMAD7	20
Cellular Assembly And Organization	Development	Development Of Cytoplasm	1.26E-05	1.371	AKAP11,AKAP9,ARHGAP18,ARHGAP6,ATG3,CD47,CIB3,CNP,COL5A3,CUL4B,DCTN1,DMD,DSTN,F2R,FKBP4,GDA,GNA12,GNG12,HRAS,IRS1,MPRIP,NET1,NRIP1,PDPK1,PES1,PEX26,PEX6,PLD1,PPP1R9A,RB1CC1,RHOD,RICTOR,ROCK1,SDC4,SNX9,SPARC,STXBP3,TMEM41B,TUBB,ULK1,UTRN,WIPI2	42
Cardiovascular Disease,Organismal Injury And Abnormalities	Fibrosis	Fibrosis Of Heart	3.21E-05	1.315	DICER1,DMD,DSP,EGR1,F11,F3,GAS2L3,GSK3A,HRAS,LMNA,MBNL2,NR3C2,PDE9A,PDGFC,PFKFB1,PIK3CA,PNKD,ROCK1,SOD2,UTRN	20
Cell Death And Survival,Organismal Injury And Abnormalities	Necrosis	Necrosis Of Epithelial Tissue	3.51E-04	1.275	ABCG2,ARHGAP18,BID,BIK,CA3,CDH1,CDH2,CEBPD,CITED2,CNP,CUL4B,CYLD,CYP7A1,DICER1,EGR1,ENDOG,ERBB3,F3,FKBP4,FOXO3,GSDMD,HRAS,HSPB1,ID2,IL18,ING4,IRS1,JAK2,MAP2K3,NCOA1,NFKBIA,NR1H4,PDGFRB,PDPK1,PPM1A,PRDX6,RB1CC1,SLC20A1,SOD2,SPARC,	42

					SPTBN1,TRIB3	
Cell Death And Survival	Cell Death	Cell Death Of Leukemia Cell Lines	6.49E-04	1.138	ABCB1,ABCB4,BID,BIK,CD47,CDH1,CEBPD,CYLD,DICER1,DPP4,EIF4EBP1,FOXO3,HLF,HSPB1,JAK2,KITLG,MAP1LC3B,NFKBIA,NR3C2,PDGFC,PPP1R1B,RCAN1,RICTOR,SOD2,TUBB6,VPS13A	26
Cell Death And Survival	Apoptosis	Apoptosis Of Mesothelioma Cell Lines	5.12E-04	1.131	BAP1,BID,BIK,DVL1,PDPK1	5
Cellular Compromise,Inflammatory Response	Degranulation	Degranulation Of Phagocytes	4.59E-04	1.117	ACLY,AGPAT2,ATP6V1D,CD47,CD9,CDA,CMTM6,CORO1B,CTSH,DGKZ,DNAJC5,DSP,GSDMD,GYG1,KCMF1,KITLG,KPNB1,LPIN1,PDPK1,PLD1,PRDX6,PSMD1,PSMD11,PSMD2,ROCK1,SLC27A2,SLC2A5,SLC9A3R1,STOM,STXBP3,TMBIM1,TRIB3,TUBB	33
Organismal Injury And Abnormalities	Benign Lesion	Benign Lesion	1.13E-06	1.098	ABCB1,ABCG2,AHNAK,ANXA7,ARIH2,ATP1A1,BAP1,BDH1,BHMT,BID,C9orf16,CDH1,CDH2,CEBPD,CLMN,CNP,COL27A1,COL5A3,CYLD,DICER1,DMD,DMTF1,DVL1,EGR1,EIF4EBP1,ERBB3,FGFR2,FH,FLCN,FOXO3,GSK3A,HRAS,ICK,ID4,ING4,KDM6A,KITLG,LRIG1,MLX,NHLRC2,NINJ1,NR1H4,NR3C2,NR5A2,NRIP1,NUDT1,OAZ1,OSBPL1A,PDGFC,PDGFRB,PGAP1,PIK3CA,PKHD1,POR,PRDX6,PSEN2,PSMA6,SERPINE2,SLC2A5,SLC30A10,SOD2,SPON2,SPOP,SPTBN1,SYNE1,TRIB3,UBAC1,UGP2,UNG,UTRN,WNT5B	71
Cell Death And Survival	Cell Viability	Cell Viability Of Tumor Cell Lines	1.27E-08	1.093	ABCB1,ABCG2,ACSL5,ATG3,BID,BUD23,CAMKK2,CAPN2,CARS1,CDH1,CDH2,CEBPD,CES1,DHX9,DIO1,DPP4,DPP9,DUSP19,EGR1,ERBB3,ESRRA,FBXO9,FGFR2,FKBP4,FOXO3,FPGS,	78

					GCLC,GSK3A,HRAS,HSPB1,ID2,ID4,IGFBP2,JAK2,JMJD1C,KDM6A,KHK,KITLG,KPNB1,LRIG1,MACROH2A1,MAP1LC3B,MAP2K3,MAP3K7,MCOLN1,NEK7,NFKBIA,NUP62,PDGFRB,PDPK1,PES1,PFDN2,PHKA2,PIK3CA,PPP1R1B,PPP1R9A,PPP2R1A,PRPF19,PRPS2,PSMA6,RB1CC1,RCAN1,RICTOR,RPA1,SHPRH,SLC2A2,SNRNP200,SNX10,SOD2,SPOP,STIP1,STX8,TBC1D16,TCP1,THRSP,UBE2L3,USP47,YPEL3	
Hematological System Development And Function	Hemostasis	Hemostasis	7.02E-04	1.093	ABCB1,ANXA7,CYP4F2,DGKZ,DMD,F11,F2R,F3,FADS2,FGA,FGB,FGG,GNA12,GSK3A,HMG20B,ITPK1,JAK2,JMJD1C,NPC1,PDPK1,PIK3CA,PRKAR2A,PSEN2,SERPINE2,ST3GAL4	25
DNA Replication, Recombination, And Repair	Cleavage	Cleavage Of DNA Fragment	7.81E-04	1.091	ENDOG,HRAS,TSN,UNG	4
Neurological Disease	Cognitive Impairment	Cognitive Impairment	4.57E-04	1	ACSL4,AHSA1,ARSG,ATP1A1,CA3,CAMKK2,CD47,CUL4B,CXADR,DDC,DMD,DPP4,DSP,FBXO31,FGFR2,FMO5,FOXO3,GCNT2,GSK3A,GSS,HMGCR,KDM6A,L2HGDH,MBNL2,MYO6,NCOA1,NR3C2,NUS1,OTUD6B,PEX6,PGAP1,PPP1R1B,PPP2R1A,PSEN2,RCAN1,SCAPER,SLC1A4,SLC35A3,SYNE1,SYT1,TBC1D20,TBC1D24,TUBB,UBR1,WDR26,WDR81	46
Cellular Assembly And Organization	Organization	Organization Of Organelle	1.10E-03	1	AFG3L2,AKAP9,ALAS1,ARHGAP21,ARHGAP6,CD2AP,CDH1,CDH2,CENPV,CHCHD3,CNP,CORO1B,CXADR,DSP,DVL1,ESRRA,FLCN,GOLGB1,HOOK1,ITPA,LMNA,LPIN1,MYO1B,PDE2A,PES1,PEX6,PPP1R9A,RPA1,RUFY3,SDC4,SERPINH1,SIRT5,SLK,SNX10,SOD2,SPA	42

					RC,SPTBN1,SYNE1,TBC1D20,TPR,TUBB,WDR81	
Cancer,Organismal Injury And Abnormalities	Malignant Neoplasm Of Retroperitoneum	Malignant Neoplasm Of Retroperitoneum	1.59E-05	-1	AASDH,ABCB1,ABCB4,ABCG2,ABTB2,ACAT2,ACLY,ACOX2,ACSM5,ADH4,AHNAK,AKAP11,AKAP9,ALAS1,ALG13,ANKRD12,ANP32A,ARHGAP21,ATXN2L,BAP1,BRD8,C9orf152,CAMKK2,CAPN2,CBLB,CCDC66,CCT3,CD9,CDC14B,CDH1,CDH2,CHD6,COL5A3,CUL4B,DCTN1,DHX9,DIAPH2,DICER1,DIO1,DMD,DOCK4,DPP4,DUSP19,E2F5,EGR1,ERBB3,ESRRA,EXOC6,EXTL2,F11,F3,FGA,FGFR2,FH,FLCN,FOXO3,FTSJ3,G6PD,GABARAP,GAS2L3,GCLC,GOLGB1,GRHPR,GTF3C2,HERC4,HMGCR,HRAS,IL18,IRF6,JAK2,KDM6A,KEAP1,KIAA0100,KLF12,KLF9,KLHL7,LMNA,LSM14B,LSS,MAGI3,MAP2K3,MIA2,MKNK2,MLLT10,MOCOS,MPDZ,MPP1,MRPS27,MYO1B,MYO5B,N4BP2L1,NAGK,NCOA1,NFKBIA,NGEF,NINJ1,NIPBL,NME1,NPC1,NR5A2,NRIP1,NUP62,PAN2,PAQR9,PCDH1,PDE2A,PDGFRB,PHKA2,PIK3CA,PKHD1,PLXNA2,PNKD,POR,PPP1R1B,PPP1R9A,PPP2R1A,PRPF6,PRPS1,PSMD1,PSMD11,PSMD2,PSMF1,PXMP4,RAI14,RB1CC1,RBBP5,RCL1,RPA1,SBF2,SCAPER,SEC63,SENP6,SERPINE2,SERPINH1,SETDB2,SHPRH,SLC1A4,SLC20A2,SLC2A5,SLC2A9,SLC39A8,SLC9A3R1,SMAD7,SNRNP200,SOD2,SPARC,SPTBN1,ST3GAL4,STARD7,STXBP3,SYNE1,TEX2,TMEM126A,TMEM51,TNRC6A,TOP1MT,	170

					TRIB3,TUBB,TUBB6,TUBGCP2,TXNL1,UBE2F,UGP2,UROD,UTP14A,UTRN,VPS13A,WNT5B,ZFAND2B,ZNF654	
Cancer,Organismal Injury And Abnormalities,Renal And Urological Disease	Neoplasia	Renal Tumor	2.41E-05	-1	ABCB1,ACOX2,AHNAK,AKAP9,ALAS1,ARHGAP21,ATXN2L,BAP1,CCT3,CD9,CDC14B,CDH1,DICER1,DIO1,DUSP19,ERBB3,EXTL2,FGFR2,FH,FLCN,FOXO3,FTSJ3,G6PD,GABARAP,GRHPR,GTF3C2,HMGCR,HRAS,ID2,IL18,IRS1,KDM6A,KEAP1,KLHL7,LMNA,MAP2K3,MLLT10,MOCOS,MPP1,N4BP2L1,NCOA1,NFKBIA,NGEF,NIPBL,NME1,NRIP1,NUP62,PCDH1,PDE2A,PDGFRB,PIK3CA,PKHD1,PNKD,POR,PPP1R1B,PPP2R1A,PSMD1,PSMD2,RAI14,RCL1,SENP6,SLC1A4,SLC20A2,SLC2A9,SLC39A8,SLC9A3R1,SMAD7,SNRNP200,SOD2,SPARC,SPTBN1,STXBP3,SYNE1,TMEM51,TUBB,TUBB6,UBE2F,UTP14A,UTRN,VPS13A	80
Cancer,Organismal Injury And Abnormalities,Renal And Urological Disease	Urinary Tract Tumor	Urinary Tract Tumor	3.53E-05	-1	ABCB1,ACOX2,AFG3L2,AHNAK,AKAP11,AKAP9,ALAS1,ANKRD12,ARHGAP21,ATP1B1,ATXN2L,BAP1,BRD8,CARS1,CCT3,CD9,CDC14B,CDH1,CHD6,CLK2,CROT,DDC,DHX9,DIAPH2,DICER1,DIO1,DIPK2A,DSP,DUSP19,ERBB3,ESRRA,EXTL2,FGA,FGFR2,FGFRL1,FH,FLCN,FOXO3,FTSJ3,G6PD,GABARAP,GOLGB1,GRHPR,GTF3C2,HMGCR,HRAS,HSPB1,ID2,IL18,IRS1,KAT2B,KDM6A,KEAP1,KLHDC2,KLHL7,KYNU,LMNA,LPIN1,LSM14B,MACROH2A1,MAP2K3,MLLT10,MOCOS,MON2,MPP1,N4BP2L1,NAGK,NCOA1,N	123

					FKBIA,NGEF,NINJ1,NIPBL,NME1,NRIP1,NUP62,OAZ1,OSBPL1A,PCDH1,PDE2A,PDGFRB,PDRG1,PIK3CA,PKHD1,PLXNA2,PNKD,POR,PPP1R1B,PPP2R1A,PSMD1,PSMD2,RAI14,RB1CC1,RCL1,RICTOR,ROCK1,SBF2,SCYL1,SEC63,SENP6,SHPRH,SLC1A4,SLC20A2,SLC2A9,SLC39A8,SLC9A3R1,SMAD7,SNRNP200,SOD2,SPARC,SPOP,SPTBN1,STXBP3,SYNE1,TBC1D16,TMEM51,TUBB,TUBB6,UBE2F,UGP2,UTP14A,UTRN,VPS13A,WIPI2	
Reproductive System Development And Function	Decidualization	Decidualization	5,91E-05	-1	CDH1,CITED2,EGR1,FGFR2,FKBP4,LGR5,NCOA1,NR5A2	8
Free Radical Scavenging,Molecular Transport	Quantity	Quantity Of Reactive Oxygen Species	3,36E-04	-1,001	BID,CA3,CITED2,DNAJB1,DPP4,FOXO3,G6PD,HRAS,IL18,JAK2,KEAP1,NFKBIA,NR1H4,OGA,PRDX6,PSEN2,SOD2,TXN2	18
Cellular Movement	Migration	Migration Of Tumor Cell Lines	3.26E-04	-1.002	ACSL4,AHNAK,AKAP11,ARHGAP21,CAMKK2,CAPN2,CBLB,CD47,CD9,CDH1,CDH2,CMTM8,CTDSP1,CTSH,DPP4,DSP,E2F5,EGR1,EIF4EBP1,ENPP1,ERBB3,ESRRA,F2R,F3,FGA,FGFR2,FH,FOXO3,GNA12,HMGCR,HRAS,ID2,IGFBP2,IL18,ING4,IRS1,JAK2,KEAP1,KITLG,LMNA,NET1,NFKBIA,NINJ1,NME1,NR1H4,NR5A2,PDLIM1,PIK3CA,PLD1,PLXNA2,PPIF,PPP1R1B,RICTOR,SDC4,SLC9A3R1,SOD2,SPARC,STK24,WDR26	59
Lipid Metabolism,Small Molecule Biochemistry,Vitamin And Mineral Metabolism	Metabolism	Metabolism Of Sterol	4.81E-09	-1.01	ABCG5,ABCG8,ACAT2,ACLY,AKR1D1,CES1,CYP39A1,CYP7A1,ELOVL6,FECH,G6PD,HMGCR,HSD17B2,HSD3B7,KPNB1,LSS,NPC1,NR1H4,POR,RAN,SOAT2	21
Cell Cycle,Cellular	Senescence	Senescence Of	3.67E-04	-1.049	AHCY,CDC14B,DICE	14

Development,Connective Tissue Development And Function		Fibroblast Cell Lines			R1,DMTF1,EGR1,FGFR2,GDA,HRAS,MAP3K7,NUDT1,PDPK1,TNRC6A,UNG,YPEL3	
Cell Cycle	Cell Cycle Progression	Cell Cycle Progression	5.78E-06	-1.051	ABCG2,ACSM5,AHNAK,AKAP9,BAP1,CAPN2,CCT4,CDH1,CITED2,CYLD,DGKZ,DHX9,DICER1,DMTF1,DPP4,E2F5,EGR1,EIF4EBP1,ERBB3,FGFR2,FGFRL1,FOXK1,FOXO3,GNA12,HECA,HMG20B,HRAS,HSPB1,ID2,IGFBP2,ING4,IRF6,IRS1,JAK2,KAT2B,KITLG,KPNB1,LGALS4,LMNA,LRIG1,MAGED1,MAP3K7,NEK7,NET1,NFKBIA,NME1,NUP62,NUP88,PDGFRB,PDPK1,PER1,PER3,PES1,PIK3CA,PPM1A,PPP1R12A,PPP2R1A,PRKAR2A,PSEN2,RAN,RASSF3,RHOD,RNF167,RPA1,SENP6,SETDB2,SLC20A1,SLC9A3R1,SMAD7,SPARC,TBRG4,TCP1,TIFA,TPR,TUBB	75
Cancer,Hematological Disease,Immunological Disease,Organismal Injury And Abnormalities	Neoplasia	Neoplasia Of Leukocytes	6.23E-05	-1.053	ABCB1,ABCG2,AHNAK,AKAP11,ANXA7,ASAP2,ATP1B1,BAP1,BID,CBLB,CCT3,CCT7,CD47,CDH1,CDO1,CEBPD,CHD6,CITED2,CNP,COL27A1,CTSH,CUL4B,DDC,DEK,DGKZ,DICER1,DIO1,DMD,DMTF1,DPP4,DSP,EGR1,EIF4EBP1,ENPP1,ERBB3,EXTL2,F2R,F3,FAM102A,FBXO31,FBXO9,FGFR2,FOXO3,FPGS,GNA12,GPD2,GRHPR,HECA,HMGCR,HRAS,HSPB1,ICK,ID2,IL18,JAK2,JMJD1C,KDM6A,KIAA0100,KITLG,KPNB1,LPIN1,LRIG1,LTBR,MAGED1,MAP2K3,MEF2A,MIA2,MKNK2,MLLT10,MPDZ,MRPL20,MYO5B,NFKBIA,NINJ1,NIPBL,NME1,PALMD,PDE9A,PDGFC,PDGFRB,PDPK1,PER1,PIK3CA,PLXNA2,PNKD,PNRC1,P	113

					OLE4,POR,PRPF19,PRPS1,PSMD1,PSMD2,RAN,RICTOR,RPA1,SARS1,SERPINH1,SHMT1,SLC25A32,SMAD7,SOD2,SPARC,SPTBN1,STIP1,STK24,SYT1,TPR,TUBB6,TUT4,UBE2F,UBR1,UNG,VPS13A	
Cancer,Gastrointestinal Disease,Organismal Injury And Abnormalities	Neoplasia	Gastrointestinal Tumor	7.05E-14	-1.054	AASDH,ABCB1,ABCB4,ABCG2,ABCG5,ABTB2,ACAD8,ACLY,ACOX2,ACSL4,ACSL5,ACSS2,ACTR6,AFG3L2,AGPAT2,AHCY,AHNAK,AHSA1,AKAP11,AKAP9,ALAS1,ALDH1L1,ALG13,ANKRD12,ANXA7,ARF3,ARHGAP18,ARHGAP21,ARHGAP6,ARIH2,ARL6IP4,ARSG,ASAP2,ASB13,ATG3,ATG4D,ATP1A1,ATP1B1,ATP6V1D,ATXN2L,BAAT,BAP1,BDH1,BHMT,BHMT2,BID,BLMH,BRD8,C11orf54,C9orf152,C9orf16,CAMKK2,CAPN2,CARS1,CBLB,CCBE1,CCDC66,CCT4,CCT5,CCT7,CD2AP,CD9,CD99L2,CDH1,CDH2,CDO1,CEBPD,CENPV,CES1,CGN,CHCHD3,CHCHD4,CHD6,CHPT1,CHRAC1,CIB3,CITED2,CLDND1,CLK2,CLMN,CLOCK,CMTM6,CNOT4,CNP,COL27A1,COL5A3,CORO1B,CPEB4,CPT1A,CRIP2,CROT,CS,CTDSP1,CTPS2,CTSH,CUL4B,CXADR,CYLD,CYP2U1,CYP4F2,CYP7A1,CYTH1,DAZAP1,DCTN1,DDC,DDX60,DEK,DGKZ,DHX9,DIAPH2,DICER1,DIO1,DIPK2A,DIXDC1,DMD,DMTF1,DNAJC5,DOCK4,DPP4,DPP9,DSP,DSTN,DUSP19,DVL1,E2F5,ECHDC1,EEPD1,EGR1,EIF4EBP1,ELF1,ERBB3,ESRRA,EXOC6,EXOSC2,F11,F2R,F3,FADS2,FAM102A,FAM160B1,FAM172A,F	471

ECH,FGA,FGB,FGFR
2,FGFRL1,FGG,FKB
P4,FLCN,FMO1,FMO
5,FOXK1,FOXO3,FP
GS,FTSJ3,G6PD,GA
S2L3,GCAT,GCLC,G
CNT2,GDA,GNA12,G
NG12,GOLGB1,GPD
2,GPN1,GRHPR,GSD
MD,GSK3A,GSS,GTF
2F1,GTF3C2,HECA,H
ECTD3,HERC4,HMG
20B,HMGCR,HNRNP
C,HNRNPD,HOMER2
,HOOK1,HRAS,HSD1
7B2,HSD3B7,HSPB1,
ICK,ID2,ID4,IGFBP2,I
L18,ING4,IRF6,IRS1,I
TPK1,JAK2,JMJD1C,
KAT2B,KCMF1,KDM6
A,KEAP1,KIAA0100,K
ITLG,KLF12,KLF9,KL
HDC2,KLHL21,KLHL
7,KPNB1,KTN1,KYN
U,LARS2,LGALS4,LG
R5,LIN7A,LIN7C,LMN
A,LRIG1,LRP12,LRR
C28,LRRC3,LSM14B,
LSS,LTBR,MACROH
2A1,MAGED1,MAGI3
,MAP2K3,MAP3K7,M
BNL2,MCOLN1,MEF2
A,MIA2,MKNK2,MLLT
10,MLX,MOCOS,MO
N2,MPDZ,MPRIP,MR
NIP,MRPL12,MRPL2
0,MRPL24,MRPS25,
MRPS27,MRPS9,MT
1F,MYO1B,MYO5B,M
YO6,N4BP2,NCALD,
NCOA1,NDUFA12,N
DUFA9,NEK7,NET1,
NFKBIA,NGEF,NHLR
C2,NIPBL,NME1,NO
SIP,NPC1,NR1H4,NR
2F6,NR3C2,NR5A2,N
RIP1,NT5C,NUDT1,N
UDT19,NUDT7,NUP6
2,NUP88,OAZ1,OSB
PL1A,OXR1,PALMD,
PAN2,PAN3,PAQR9,
PCDH1,PCGF5,PDE2
A,PDE9A,PDGFC,PD
GFRB,PDLIM1,PDP2,
PDPK1,PDRG1,PER1
,PER3,PES1,PEX6,P
FDN2,PGAP1,PHF20
L1,PHKA2,PIK3AP1,
PIK3CA,PKHD1,PLD
1,PLXNA2,PMPCA,P
NKD,PNRC1,POR,PP
M1A,PPOX,PPP1R12

A,PPP1R14B,PPP1R
1B,PPP1R3B,PPP1R
9A,PPP2R1A,PRDX6,
PRPF39,PRPF6,PRP
S1,PSMD1,PSMD11,
PSMD8,PSPC1,PSP
H,PTBP1,QDPR,RAB
11FIP2,RABGAP1L,R
AI14,RALGPS2,RAN,
RASA3,RASSF3,RB1
CC1,RBBP5,RBBP7,
REPIN1,RGS2,RHBD
D1,RICTOR,ROCK1,
RPA1,RUFY3,SAA4,
SAR1B,SARS1,SBF2,
SCAPER,SCARB2,S
CYL1,SDC4,SEC63,S
ECISBP2L,SENP6,S
ERPINA6,SERPINE2,
SERPINH1,SESN3,S
ETDB2,SFRP5,SGK2
,SGTB,SH3D19,SHM
T1,SHPRH,SIRT5,SL
C1A4,SLC20A1,SLC2
0A2,SLC27A2,SLC2A
2,SLC2A5,SLC2A9,S
LC30A10,SLC35B3,S
LC38A2,SLC39A10,S
LC39A8,SLC6A12,SL
K,SMAD7,SMAP1,SN
APC2,SNRNP200,SN
TB1,SNX10,SNX9,SO
AT2,SOD2,SPARC,S
PG21,SPON2,SPOP,
SPTBN1,SPTBN2,ST
3GAL4,STIP1,STK40,
STOM,STT3B,STX8,
STXBP3,SULF2,SYA
P1,SYNE1,SYT1,TBC
1D16,TBC1D20,TBC1
D24,TBRG4,TCP1,TE
P1,TEX2,THAP4,THR
SP,TIGD2,TIMM44,T
K1,TLE1,TMBIM1,TM
EM126A,TMEM219,T
MEM41A,TMEM41B,
TMEM62,TMEM97,T
NFAIP8L1,TNRC6A,T
OP1MT,TPI1,TPR,TP
RKB,TRIB3,TRMT1,T
SPAN33,TTC1,TTC1
9,TUBB,TUBB6,TUB
GCP2,TXNL1,UBAC1
,UBE2L3,UBR1,UBTF
,UBXN1,UBXN6,UCK
1,UGP2,ULK1,UNG,U
PP2,UROC1,UROD,U
SP47,UTP14A,UTRN,
UXS1,VPS13A,WDR1
8,WDR26,WDR81,WI
PI2,WNT5B,YWHAH,
ZCCHC24,ZNF23,ZN

					F32,ZNF326	
Cell-To-Cell Signaling And Interaction	Interaction	Interaction Of Tumor Cell Lines	2,01E-04	-1,057	ARHGAP21,CD47,CD9,CDH1,CDH2,CXADR,CYTH1,DPP4,DSP,EGR1,ERBB3,F2R,F3,FGA,FGFR2,HRAS,IL18,IRS1,JAK2,KITLG,LGALS4,LTBR,MYO6,NET1,NFKBIA,NME1,NR1H4,PLD1,RCAN1,RICTOR,TCIM,UTRN	32
Cellular Compromise	Nucleation	Nucleation Of Cells	1.02E-03	-1.067	CD2AP,GAS2L3,HRAS,KPNB1,LMNA,NEK7,PPP1R12A,RHOD	8
Cancer,Gastrointestinal Disease,Organismal Injury And Abnormalities	Large Intestine Neoplasm	Large Intestine Neoplasm	1.52E-14	-1.091	AASDH,ABCB1,ABCB4,ABCG2,ABCG5,ABTB2,ACAD8,ACLY,ACOX2,ACSL4,ACSL5,ACSS2,ACTR6,AFG3L2,AGPAT2,AHCY,AHNAK,AHSA1,AKAP11,AKAP9,ALAS1,ALDH1L1,ALG13,ANKRD12,ANXA7,ARF3,ARHGAP18,ARHGAP21,ARHGAP6,ARIH2,ARL6IP4,ARSG,ASAP2,ASB13,ATG3,ATG4D,ATP1A1,ATP1B1,ATP6V1D,ATXN2L,BAAT,BAP1,BDH1,BHMT2,BID,BLMH,BRD8,C11orf54,C9orf152,C9orf16,CAMKK2,CAPN2,CARS1,CBLB,CCBE1,CCDC66,CCT4,CCT5,CCT7,CD2AP,CD9,CD99L2,CDH1,CDH2,CDO1,CEBPD,CENPV,CES1,CGN,CHCHD3,CHCHD4,CHD6,CHPT1,CHRAC1,CIB3,CITED2,CLDND1,CLK2,CLMN,CLOCK,CMTM6,CNOT4,CNP,COL27A1,COL5A3,CORO1B,CPEB4,CPT1A,CRIP2,CROT,CS,CTDSP1,CTPS2,CUL4B,CXADR,CYLD,CYP2U1,CYP4F2,CYP7A1,CYTH1,DAZAP1,DCTN1,DDC,DDX60,DEK,DGKZ,DHX9,DIAPH2,DICER1,DIO1,DIPK2A,DIXDC1,DMD,DMTF1,DNAJC5,DOCK4,DPP4,DPP9,DSP,DSTN,DUSP19,DVL1,ECHDC1,EEPD1,EGR1,EIF4EBP1,EL	457

F1,ERBB3,ESRRA,E
XOC6,EXOSC2,F11,
F2R,F3,FADS2,FAM1
02A,FAM160B1,FAM
172A,FECH,FGA,FG
B,FGFR2,FGFRL1,F
GG,FKBP4,FLCN,FM
O1,FMO5,FOXK1,FP
GS,FTSJ3,G6PD,GA
S2L3,GCAT,GCLC,G
CNT2,GDA,GNA12,G
NG12,GOLGB1,GPD
2,GPN1,GRHPR,GSD
MD,GSK3A,GSS,GTF
2F1,GTF3C2,HECA,H
ECTD3,HERC4,HMG
20B,HMGCR,HNRNP
C,HNRNPD,HOMER2
,HOOK1,HRAS,HSD1
7B2,HSD3B7,HSPB1,
ICK,ID2,ID4,IGFBP2,I
L18,ING4,IRF6,IRS1,I
TPK1,JAK2,JMJD1C,
KAT2B,KCMF1,KDM6
A,KEAP1,KIAA0100,K
ITLG,KLF12,KLF9,KL
HDC2,KLHL21,KLHL
7,KPNB1,KTN1,KYN
U,LARS2,LGALS4,LG
R5,LIN7A,LMNA,LRI
G1,LRP12,LRRC28,L
RRC3,LSM14B,LSS,L
TBR,MACROH2A1,M
AGED1,MAGI3,MAP2
K3,MAP3K7,MBNL2,
MEF2A,MIA2,MKNK2
,MLLT10,MLX,MOCO
S,MON2,MPDZ,MPRI
P,MRNIP,MRPL12,M
RPL20,MRPL24,MRP
S25,MRPS27,MRPS9
,MT1F,MYO1B,MYO5
B,MYO6,N4BP2,NCA
LD,NCOA1,NDUFA12
,NDUFA9,NEK7,NET
1,NFKBIA,NGEF,NHL
RC2,NIPBL,NME1,N
OSIP,NPC1,NR1H4,N
R2F6,NR3C2,NR5A2,
NRIP1,NT5C,NUDT1,
NUDT19,NUDT7,NUP
62,NUP88,OAZ1,OSB
PL1A,OXR1,PALMD,
PAN2,PAN3,PAQR9,
PCDH1,PCGF5,PDE2
A,PDE9A,PDGFC,PD
GFRB,PDLIM1,PDP2,
PDPK1,PDRG1,PER1
,PER3,PEX6,PFDN2,
PGAP1,PHF20L1,PH
KA2,PIK3AP1,PIK3C
A,PKHD1,PLD1,PLX

NA2,PMPCA,PNKD,P
NRC1,POR,PPM1A,P
POX,PPP1R12A,PPP
1R14B,PPP1R1B,PP
P1R3B,PPP1R9A,PP
P2R1A,PRDX6,PRPF
39,PRPF6,PRPS1,PS
MD1,PSMD11,PSMD
8,PSPC1,PSPH,PTB
P1,QDPR,RAB11FIP
2,RABGAP1L,RAI14,
RALGPS2,RAN,RAS
A3,RASSF3,RB1CC1,
RBBP5,RBBP7,REPI
N1,RHBDD1,RICTOR
,ROCK1,RPA1,RUFY
3,SAA4,SAR1B,SAR
S1,SBF2,SCAPER,S
CYL1,SEC63,SECISB
P2L,SENP6,SERPIN
A6,SERPINE2,SERPI
NH1,SESN3,SETDB2
,SFRP5,SGK2,SGTB,
SH3D19,SHMT1,SHP
RH,SIRT5,SLC1A4,S
LC20A1,SLC20A2,SL
C27A2,SLC2A2,SLC2
A5,SLC2A9,SLC30A1
0,SLC35B3,SLC38A2
,SLC39A10,SLC39A8
,SLC6A12,SLK,SMAD
7,SMAP1,SNAPC2,S
NRNP200,SNX10,SN
X9,SOAT2,SOD2,SP
ARC,SPG21,SPOP,S
PTBN1,SPTBN2,ST3
GAL4,STIP1,STK40,S
TOM,STT3B,STX8,S
TXBP3,SULF2,SYAP
1,SYNE1,SYT1,TBC1
D16,TBC1D20,TBC1
D24,TBRG4,TCP1,TE
P1,TEX2,THAP4,THR
SP,TIGD2,TIMM44,T
K1,TLE1,TMBIM1,TM
EM126A,TMEM219,T
MEM41A,TMEM41B,
TMEM62,TMEM97,T
NFAIP8L1,TNRC6A,T
OP1MT,TPI1,TPR,TP
RKB,TRIB3,TRMT1,T
SPAN33,TTC1,TTC1
9,TUBB,TUBB6,TUB
GCP2,TXNL1,UBAC1
,UBE2L3,UBR1,UBTF
,UBXN1,UBXN6,UCK
1,UGP2,ULK1,UNG,U
PP2,UROC1,UROD,U
SP47,UTRN,UXS1,V
PS13A,WDR18,WDR
26,WDR81,WIPI2,WN
T5B,YWHAH,ZCCHC

					24,ZNF23,ZNF326	
Cancer,Gastrointestinal Disease,Organismal Injury And Abnormalities	Colorectal Neoplasia	Colorectal Tumor	3.84E-07	-1.091	AASDH,ABCB1,ABCG2,ABCG5,ACLY,ACOX2,ACSL5,ACSS2,ACTR6,AFG3L2,AGPAT2,AHCY,AKAP9,ALAS1,ALDH1L1,ALG13,ANXA7,ARHGAP21,ARL6IP4,ASAP2,ASB13,ATG3,ATG4D,ATXN2L,BAAT,BAP1,BID,C11orf54,C9orf152,C9orf16,CARS1,CCDC66,CCT4,CCT5,CCT7,CD2AP,CD9,CD99L2,CDH1,CDH2,CDO1,CEBPD,CES1,CGN,CHCHD3,CHD6,CHPT1,CHRAC1,CLDND1,CLK2,CLMN,CLOCK,CMTM6,CNOT4,CNP,COL27A1,COL5A3,CPEB4,CPT1A,CS,CTDSP1,CTPS2,CUL4B,CYLD,CYP4F2,DCTN1,DDX60,DGKZ,DHX9,DICER1,DMD,DMTF1,DOCK4,DSTN,EGR1,EIF4EBP1,ELF1,ERBB3,ESRRA,F2R,F3,FAM160B1,FECH,FGA,FGB,FGFR2,FGG,FLCN,FMO1,FMO5,FPGS,G6PD,GAS2L3,GNA12,GNG12,GSS,GTF2F1,HECA,HECTD3,HMGCR,HNRNPC,HRAS,HSD17B2,HSPB1,ICK,ID2,IGFBP2,IL18,ING4,IRF6,IRS1,JAK2,JMJD1C,KAT2B,KDM6A,KEAP1,KIAA0100,KITLG,KLF12,KLHL7,KPNB1,KTN1,LGALS4,LGR5,LIN7A,LRIG1,LRP12,LRRC3,LTBR,MACROH2A1,MAGED1,MAP3K7,MEF2A,MIA2,MKNK2,MLLT10,MLX,MON2,MPDZ,MPRIP,MRNIP,MRPS9,MT1F,MYO5B,MYO6,NCOA1,NEK7,NET1,NGEF,NHLRC2,NIPBL,NME1,NOSIP,NR1H4,NR3C2,NR5A2,NT5C,NUDT19,NUDT7,NUP62,OAZ1,OSBPL1A,PAQR9,PCDH1,PCGF5,PDE9A,PDGFRB,PDLIM1,PDP2,PDPK1,PDRG1,	271

					PER1,PFDN2,PGAP1,PHF20L1,PHKA2,PIK3AP1,PIK3CA,PKHD1,PLD1,PLXNA2,PNKD,PNRC1,POR,PPP1R14B,PPP1R1B,PPP1R9A,PPP2R1A,PRDX6,PRPF39,PRPF6,PSMD8,PTBP1,QDPR,RABGAP1L,RASSF3,RB1CC1,RBBP5,RICTOR,ROCK1,RPA1,RUFY3,SAA4,SBF2,SCAPER,SCYL1,SEC63,SECISBP2L,SENP6,SERPINE2,SESN3,SGK2,SHPRH,SLC1A4,SLC20A1,SLC20A2,SLC27A2,SLC2A2,SLC30A10,SLC35B3,SLC38A2,SLC39A10,SLC39A8,SLC6A12,SMAD7,SMAP1,SNRNP200,SNX9,SOAT2,SOD2,SPARC,SPOP,SPTBN1,SPTBN2,STIP1,STX8,SULF2,SYAP1,SYNE1,SYT1,TBC1D16,TBC1D24,TBRG4,TCP1,TEP1,TIMM44,TK1,TMEM126A,TMEM41B,TMEM97,TNRC6A,TOP1MT,TPI1,TPR,TRIB3,TTC1,TUBB,UBAC1,UBTF,UGP2,ULK1,UNG,UROC1,UTRN,VPS13A,WDR18,WDR26,WDR81,WNT5B,ZCCHC24,ZNF23	
Lipid Metabolism,Molecular Transport,Small Molecule Biochemistry	Concentration	Concentration Of Lipid	4.91E-07	-1.095	ABCB1,ABCB4,ABCG2,ABCG5,ABCG8,ACAT2,ACLY,ACSL4,AGPAT2,ATP1A1,BHMT,BID,CES1,CLOCK,CPT1A,CYP4F2,CYP7A1,DGKZ,DICER1,DIO1,EGR1,F2R,FADS2,FGFR2,FKBP4,FMO5,FOXO3,G6PD,GPD2,GSK3A,HMGCR,HRAS,HSD3B7,IL18,IRS1,JAK2,KITLG,LGR5,LMNA,LPIN1,MACROH2A1,NCOA1,NPC1,NR1H4,NR5A2,NUS1,OGA,PDGFRB,PFKFB1,PIK3CA,PLD1,POR,PRDX6,PRPF19,PSEN2,ROCK1,SERPINA6,SIRT5,SLC2A2,SLC9A3R1,SOAT	65

					2,SOD2,STARD4,THRSP,UBR1	
Lipid Metabolism,Small Molecule Biochemistry	Metabolism	Fatty Acid Metabolism	3.36E-08	-1.107	ABCB1,ABCB4,ABCG2,ABCG5,ABCG8,ACLY,ACSL3,ACSL4,ACSL5,ACSM1,ACSS2,BAAT,CD9,CES1,CPT1A,CROT,CS,CYP4F2,CYP7A1,EGR1,EIF4EBP1,ELOVL6,F2R,FADS2,FH,FOXO3,HLF,HRAS,IL18,JAK2,KITLG,LPIN1,LSS,MAP2K3,NFKBIA,NPC1,NR1H4,NUDT19,NUDT7,OSBPL1A,POR,PRDX6,SCARB2,SERPINA6,SLC10A1,SLC27A2,SLC38A2,SLC9A3R1,SNTB1,SOAT2,STARD4,THRSP,TRIB3	53
Cancer,Organismal Injury And Abnormalities	Neoplasia	Epithelial Neoplasm	1.54E-28	-1.125	AASDH,ABCB1,ABCB4,ABCG2,ABCG5,ABCG8,ABTB2,ACAD8,ACAT2,ACLY,ACOX2,ACSL3,ACSL4,ACSL5,ACSM1,ACSM5,ACSS2,ACTR6,ADH4,AFG3L2,AGPAT2,AHCY,AHNAK,AHSA1,AKAP11,AKAP9,AKR1D1,ALAS1,ALDH1L1,ALG13,AMDHD1,ANKRD12,ANP32A,ANXA7,ARF3,ARGLU1,ARHGAP18,ARHGAP21,ARHGAP6,ARIH2,ARL15,ARL4A,ARL6IP4,ARSG,ASAP2,ASB13,ATG3,ATG4D,ATP1A1,ATP1B1,ATP6V1D,ATXN2L,AUH,BAAT,BAP1,BDH1,BHMT,BHMT2,BID,BIK,BLMH,BRD8,BUD23,C11orf54,C9orf152,C9orf16,CA3,CAMKK2,CAPN2,CARS1,CBLB,CCBE1,CCDC66,CCT3,CCT4,CCT5,CCT6A,CCT7,CD2AP,CD47,CD9,CD99L2,CDA,CDC14B,CDH1,CDH2,CDO1,CEBPD,CENPV,CES1,CGN,CHCHD3,CHCHD4,CHD6,CHPT1,CHRAC1,CIB3,CITED2,CLDND1,CLK2,CLMN,CLOCK,CMTM6,CMTM8,CNN3,CNOT4,CNOT6,CNP,C	583

OL27A1,COL5A3,CO
RO1B,CPEB4,CPT1A
,CRIP2,CRLS1,CROT
,CS,CSRNP1,CTDSP
1,CTPS2,CTSH,CUL4
B,CXADR,CYB5B,CY
LD,CYP2U1,CYP39A
1,CYP4F2,CYP7A1,C
YTH1,DAZAP1,DCTN
1,DDC,DDX60,DEK,D
GKZ,DHX9,DIAPH2,D
ICER1,DIO1,DIPK2A,
DIXDC1,DMD,DMTF1
,DNAJB1,DNAJC5,D
OCK4,DPP4,DPP9,D
SP,DSTN,DTYMK,DU
SP19,DVL1,E2F5,EC
HDC1,EEPD1,EFHD2
,EGR1,EIF1AX,EIF4E
BP1,ELF1,ENDOG,E
NPP1,ERBB3,ESRRA
,EXOC6,EXOSC2,EX
TL2,F11,F2R,F3,FAD
S2,FAM102A,FAM11
8B,FAM160B1,FAM1
72A,FAM76B,FAM89
A,FBXO31,FBXO9,FE
CH,FGA,FGB,FGFR2
,FGFRL1,FGG,FH,FK
BP4,FLCN,FMO1,FM
O5,FOXK1,FOXO3,F
PGS,FTSJ3,G6PD,G
AS2L3,GCAT,GCLC,
GCNT2,GDA,GNA12,
GNG12,GOLGB1,GP
D2,GPN1,GRHPR,GS
DMD,GSK3A,GSS,G
STM2,GTF2A2,GTF2
F1,GTF3C2,GYG1,H
ECA,HECTD3,HERC
4,HLF,HMG20B,HMG
CR,HNRNPC,HNRNP
D,HOMER2,HOOK1,
HRAS,HSD17B2,HSD
3B7,HSPB1,ICK,ID2,I
D4,IGFBP2,IL18,ING
4,IRF6,IRS1,ITPA,ITP
K1,JAK2,JMJD1C,KA
T2B,KCMF1,KDM6A,
KEAP1,KHK,KIAA010
0,KITLG,KLF12,KLF9,
KLHDC2,KLHL21,KL
HL7,KPNB1,KTN1,KY
NU,L2HGDH,LARS2,
LGALS4,LGR5,LIN7A
,LIN7C,LITAF,LMNA,
LPIN1,LRIG1,LRP12,
LRRC28,LRRC3,LSM
14B,LSS,LTBR,MAC
ROH2A1,MAGED1,M
AGI3,MAP1LC3B,MA
P2K3,MAP3K7,MBNL

2,MCOLN1,MEF2A,M
IA2,MKNK2,MLLT10,
MLX,MMD,MOCOS,M
ON2,MPDZ,MPP1,M
PRIP,MRNIP,MRPL1
2,MRPL20,MRPL24,
MRPL44,MRPS22,M
RPS25,MRPS27,MR
PS9,MT1F,MYO1B,M
YO5B,MYO6,N4BP2,
N4BP2L1,NAGK,NCA
LD,NCOA1,NDUFA12
,NDUFA9,NDUFB9,N
EK7,NET1,NFKBIA,N
GEF,NHLRC2,NINJ1,
NIPBL,NME1,NOSIP,
NPC1,NR1H4,NR2F6
,NR3C2,NR5A2,NRIP
1,NT5C,NUDT1,NUD
T19,NUDT7,NUP62,N
UP88,NUS1,OAZ1,O
SBPL1A,OTUD6B,OX
R1,PALMD,PAN2,PA
N3,PAQR9,PCDH1,P
CGF5,PDCD2,PDE2A
,PDE9A,PDGFC,PDG
FRB,PDLIM1,PDP2,P
DPK1,PDRG1,PER1,
PER3,PES1,PEX26,P
EX6,PFDN2,PFKFB1,
PGAP1,PHF20L1,PH
KA2,PIK3AP1,PIK3C
A,PKHD1,PLD1,PLX
NA2,PMPCA,PNKD,P
NRC1,POR,PPIF,PP
M1A,PPOX,PPP1R12
A,PPP1R14B,PPP1R
1B,PPP1R3B,PPP1R
9A,PPP2R1A,PRDX6,
PRPF19,PRPF39,PR
PF6,PRPS1,PRPS2,P
SEN2,PSMA6,PSMD
1,PSMD11,PSMD2,P
SMD8,PSME2,PSMG
1,PSPC1,PSPH,PTB
P1,PTBP2,PXMP4,Q
DPR,RAB11FIP2,RA
B1B,RABEPK,RABG
AP1L,RAI14,RALGPS
2,RAN,RASA3,RASS
F3,RB1CC1,RBBP5,
RBBP7,RBKS,RCAN
1,RCL1,REPIN1,RGS
2,RHBDD1,RHOD,RI
CTOR,RNF14,RNF14
4A,RNF167,ROCK1,
RPA1,RPIA,RUFY3,S
AA4,SAR1B,SARS1,
SBF2,SCAPER,SCA
RB2,SCYL1,SDC4,S
DR42E1,SEC63,SECI
SBP2L,SENP6,SERP

					INA6,SERPINE2,SERPINH1,SESN3,SETDB2,SFRP5,SGK2,SGTB,SH3D19,SHMT1,SHPRH,SIRT5,SLC10A1,SLC16A5,SLC1A4,SLC20A1,SLC20A2,SLC25A32,SLC27A2,SLC2A2,SLC2A5,SLC2A9,SLC30A10,SLC35A3,SLC35B1,SLC35B3,SLC38A2,SLC39A10,SLC39A8,SLC6A12,SLC9A3R1,SLK,SMAD7,SMAP1,SNAPC2,SNRNP200,SNTB1,SNX10,SNX5,SNX9,SOAT2,SOD2,SPARC,SPG21,SPON2,SPOP,SPTBN1,SPTBN2,ST3GAL4,STARD7,STIP1,STK24,STK40,STOM,STT3B,STX18,STX8,STXBP3,SULF2,SUMF1,SYAP1,SYNE1,SYT1,TBC1D16,TBC1D20,TBC1D24,TBRG4,TCIM,TCP1,TEP1,TEX2,THAP4,THRSP,TIFA,TIGD2,TIMM10,TIMM44,TK1,TLCD4,TLE1,TMBIM1,TMEM126A,TMEM219,TMEM41A,TMEM41B,TMEM51,TMEM62,TMEM97,TNFAIP2,TNFAIP8L1,TNRC6A,TOP1MT,TPI1,TPR,TPRG1L,TPRKB,TRIB3,TRMT1,TSN,TSPAN33,TSTA3,TTC1,TTC19,TUBB,TUBB6,TUBGCP2,TXN2,TXNL1,UBAC1,UBE2F,UBE2L3,UBR1,UBTF,UBXN1,UBXN6,UCK1,UGP2,UGT2B10,ULK1,UNG,UPP2,UROC1,UROD,USP47,UTP14A,UTRN,UXS1,VPS13A,WDR18,WDR26,WDR81,WIPI2,WNT5B,YPEL3,YWHAH,ZCCHC24,ZFAND2B,ZKSCAN1,ZNF23,ZNF32,ZNF326,ZNF654	
Cancer,Organismal Injury And Abnormalities,Reproductive System Disease	Neoplasia	Prostatic Tumor	3.33E-06	-1.131	ABCB1,ABCB4,ABCG2,ACAD8,ACLY,ACOX2,ACSL3,ACSL4,ACSM1,ACSS2,AGPAT2,AHNAK,AKAP11,ALDH1L1,ALG13,ANK	198

RD12,ARHGAP18,AR
HGAP6,ARIH2,ASAP
2,ATP1A1,ATP6V1D,
ATXN2L,BAAT,BAP1,
BDH1,BRD8,CA3,CB
LB,CD2AP,CDH1,CD
H2,CDO1,CES1,CGN
,CHD6,CLDND1,CNO
T4,CNOT6,CNP,COL
5A3,CPT1A,CSRNP1,
CYP7A1,CYTH1,DCT
N1,DDX60,DGKZ,DIC
ER1,DMD,DOCK4,DS
P,EFHD2,EGR1,EIF1
AX,EIF4EBP1,ERBB3
,F11,FAM160B1,FAM
89A,FGA,FGFR2,FG
FRL1,FH,FKBP4,FLC
N,FOXK1,FOXO3,GD
A,GNA12,GOLGB1,G
PD2,GSDMD,GTF2A
2,HLF,HMGCR,HRAS
,HSD3B7,HSPB1,ID2,
IGFBP2,ING4,IRS1,J
AK2,JMJD1C,KDM6A
,KIAA0100,KTN1,LAR
S2,LGALS4,LGR5,LI
N7C,LITAF,LRIG1,LR
P12,LRRC28,LSS,LT
BR,MAGI3,MBNL2,MI
A2,MKNK2,MLLT10,
MMD,MOCOS,MON2,
MPDZ,MPRIP,MRPS
25,MYO5B,MYO6,N4
BP2,NCOA1,NFKBIA,
NIPBL,NME1,NRIP1,
NUDT7,NUP88,OAZ1
,OTUD6B,PAN3,PCD
H1,PDGFRB,PDP2,P
DPK1,PER1,PEX6,PI
K3CA,PKHD1,PLD1,
PLXNA2,POR,PPIF,P
PP1R1B,PPP1R3B,P
PP1R9A,PPP2R1A,P
RPF6,PSEN2,PSMD1
,PSMD2,PXMP4,RAB
11FIP2,RALGPS2,RC
AN1,RCL1,REPIN1,R
HOD,RNF144A,ROC
K1,SBF2,SCAPER,S
CARB2,SCYL1,SDC4
,SDR42E1,SECISBP2
L,SERPINA6,SERPIN
E2,SESN3,SIRT5,SL
C16A5,SLC1A4,SLC2
5A32,SLC39A10,SLC
6A12,SLK,SMAP1,SN
RNP200,SPARC,SPO
P,SPTBN1,SPTBN2,
ST3GAL4,STX8,STX
BP3,SULF2,SYNE1,T
BC1D16,TBC1D24,T

					K1,TNFAIP2,TNRC6A,TOP1MT,TPR,TRMT1,TSPAN33,UBAC1,UBE2F,UBR1,UROC1,USP47,UTRN,VPS13A,YPEL3,ZKSCAN1,ZNF654	
Cancer,Organismal Injury And Abnormalities,Reproductive System Disease	Neoplasia	Neoplasia Of Mammary Gland	1.06E-03	-1.131	CEBPD,ERBB3,HRAS,IRS1,JAK2,PIK3CA	6
Cancer,Organismal Injury And Abnormalities	Development	Development Of Adenocarcinoma	5.73E-09	-1.134	ABCB1,ABCB4,ABCG5,ACAD8,ACOX2,ACSL3,ACSL4,ACSM1,ACSS2,AGPAT2,AHNAK,AKAP11,AKAP9,ALAS1,ALDH1L1,ALG13,ANKRD12,ANP32A,ARHGAP21,ARHGAP6,ARIH2,ARL15,ATXN2L,AUH,BAP1,BDH1,BRD8,BUD23,CAPN2,CCT3,CCT5,CCT6A,CCT7,CD47,CD9,CDC14B,CDH1,CDH2,CDO1,CES1,CGN,CHD6,CLMN,CMTM8,CNOT4,CNOT6,COL27A1,COL5A3,CPEB4,CS,CUL4B,CXADR,CYP4F2,CYP7A1,DAZAP1,DDX60,DEK,DHX9,DIAPH2,DICER1,DIO1,DIPK2A,DIXDC1,DMD,DOCK4,DPP4,DPP9,DSP,DSTN,DUSP19,E2F5,EIF1AX,ELF1,ERBB3,EXOC6,EXTL2,F11,F2R,F3,FAM160B1,FECH,FGA,FGB,FGFR2,FGG,FH,FLCN,FOXO3,FTSJ3,G6PD,GAS2L3,GNA12,GOLGB1,GRHPR,GSK3A,GSTM2,GTF3C2,GYG1,HECA,HECTD3,HERC4,HMGCR,HRAS,ICK,IGFBP2,IL18,ING4,IRF6,IRS1,ITPA,JAK2,JMJD1C,KAT2B,KCMF1,KDM6A,KEAP1,KIAA0100,KLHL7,KTN1,KYNU,L2HGDH,LGR5,LMNA,LRIG1,LRRC28,LSM14B,MAGED1,MAGI3,MAP2K3,MCOLN1,MEF2A,MIA2,MLLT10,MOCOS,MON2,MPDZ,MPP1,MPRIP,MRPL44,MRPS22,	262

					MRPS27,MYO5B,MYO6,N4BP2L1,NCOA1,NDUFA9,NET1,NFKBIA,NGEF,NHLRC2,NIPBL,NME1,NOSIP,NPC1,NRIP1,NUDT1,NUP62,NUP88,OSBPL1A,OXR1,PALMD,PAN2,PAN3,PCGF5,PDCD2,PDE2A,PDGFRB,PDLIM1,PDP2,PER1,PER3,PGAP1,PHF20L1,PHKA2,PIK3CA,PKHD1,PLXNA2,PNKD,POR,PPP1R1B,PPP1R9A,PPP2R1A,PRPF19,PRPF6,PSMD1,PSMD2,PTBP2,RABEPK,RABGAP1L,RAI14,RALGPS2,RB1CC1,RBBP5,RBBP7,RCL1,RHBDD1,RICTOR,ROCK1,RPA1,RPIA,SBF2,SCAPER,SECISBP2L,SENP6,SESN3,SETDB2,SLC1A4,SLC20A1,SLC20A2,SLC2A2,SLC2A9,SLC35B3,SLC39A10,SLC39A8,SLC9A3R1,SMAD7,SNRNP200,SNX10,SOD2,SPARC,SPON2,SPOP,SPTBN1,SPTBN2,STARD7,STK24,STK40,STOM,STT3B,STX8,STXBP3,SYNE1,TBC1D20,TBRG4,TEP1,TIGD2,TK1,TLCD4,TLE1,TMEM126A,TMEM51,TNFAIP2,TOP1MT,TPI1,TPR,TPRG1L,TRIB3,TTC1,TUBB,TUBB6,TXNL1,UBE2F,UGP2,UGT2B10,UTP14A,UTRN,UXS1,VPS13A,ZCCHC24,ZFAND2B,ZKSCAN1,ZNF32	
Cancer,Organismal Injury And Abnormalities	Lymphoreticular Neoplasm	Lymphoreticular Neoplasm	6,06E-05	-1,152	ABCB1,ABCB4,ABCG2,ABCG5,ABCG8,ACSL4,AHNAK,AKAP11,ALAS1,ANXA7,ASAP2,ATG4D,ATP1A1,ATP1B1,BAAT,BAP1,BHMT,CAPN2,CBLB,CCT3,CCT7,CD47,CDA,CDH1,CDO1,CHD6,CNP,COL27A1,CPT1A,CTPS2,CTSH,DAZAP1,DDX60,DEK,DGKZ,DHX9,DICER1,DIPK2A,DMD,DMTF1,D	154

					NAJC5,DPP4,DSP,ECHDC3,EGR1,EIF4EBP1,ERBB3,F3,FAM102A,FBXO31,FGFR2,FGG,FOXO3,FPGS,GNA12,GPD2,GYG1,HECA,HECTD3,HMG20B,HMGCR,HNRNPD,HOOK1,HRAS,ICK,ID2,ID4,IL18,IRS1,JAK2,JMJD1C,KDM6A,KIAA0100,KITLG,KPNB1,LARS2,LGALS4,LPIN1,LRIG1,LRRC3,MAP2K3,MEF2A,MIA2,MKNK2,MLLT10,MPDZ,MRPL20,MRPL44,MYO5B,NET1,NFKBIA,NINJ1,NME1,NRIP1,NUP88,OSBPL1A,PALMD,PAN3,PAQR9,PDGFC,PDGFRB,PDPK1,PER1,PER3,PES1,PEX6,PIK3AP1,PIK3CA,PKHD1,PLXNA2,POLE4,POR,PPM1A,PPP1R14B,PPP1R1B,PPP1R3B,PRPS1,PSEN2,PSMD1,PSMD2,PSME2,RAN,RHOD,RICTOR,RPA1,SARS1,SCARB2,SERPINH1,SGK2,SHMT1,SHPRH,SMAD7,SOAT2,SOD2,SPARC,SPTBN1,STIP1,STK24,STK40,SYNE1,SYT1,TCIM,TEP1,TPI1,TPR,TUBB6,TUT4,UBE2F,UBR1,UBXN1,UGT2B10,UNG,UROC1,VPS13A	
Endocrine System Disorders,Gastrointestinal Disease,Organismal Injury And Abnormalities	Pancreatic Mass	Pancreatic Mass	6.08E-04	-1.154	AASDH,ABCB4,ABCG2,ABTB2,ACAT2,ACLY,ACSM5,ADH4,AHNAK,AKAP11,AKAP9,ALG13,ANKRD12,ANP32A,BAP1,BRD8,C9orf152,CAMKK2,CAPN2,CBLB,CCDC66,CDH1,CDH2,CHD6,COL5A3,CUL4B,CYLD,CYP4F2,DCTN1,DHX9,DIAPH2,DMD,DOCK4,DPP4,E2F5,ECHDC3,EGR1,EIF1AX,ERBB3,ESRRA,EXOC6,F11,F3,FGA,FGFR2,FMO1,FTSJ3,GAS2L3,GCLC,GOLGB1,GRHPR,HERC4,HMGCR,HNRNPC,HRAS	140

					,IL18,IRF6,JAK2,KDM6A,KEAP1,KIAA0100,KLF12,KLF9,LGR5,LIN7A,LMNA,LRIG1,LSM14B,LSS,MAGI3,MIA2,MKNK2,MPDZ,MRPL24,MRPS27,MYO1B,MYO5B,NAGK,NCOA1,NGEF,NINJ1,NIPBL,NPC1,NR5A2,PAN2,PAQR9,PCDH1,PDGFRB,PHKA2,PIK3CA,PKHD1,PLXNA2,POR,PPP1R9A,PPP2R1A,PRPF6,PRPS1,PSMD1,PSMD11,PSMF1,PXMP4,RAB11FIP2,RB1CC1,RBBP5,RHOD,RPA1,SBF2,SCAPER,SEC63,SERPINE2,SERPINH1,SETDB2,SHMT1,SHPRH,SLC20A2,SLC2A5,SLC2A9,SLC35B1,SLC9A3R1,SMAD7,SNX10,SPTBN1,ST3GAL4,STARD7,SYNE1,TEP1,TEX2,TMEM126A,TNRC6A,TOP1MT,TRIB3,TUBB6,TUBGCP2,TXNL1,UGP2,UROD,UTRN,WNT5B,ZFAND2B,ZNF654	
Cancer,Endocrine System Disorders,Gastrointestinal Disease,Organismal Injury And Abnormalities	Pancreatic Tumor	Pancreatic Tumor	8.25E-04	-1.154	AASDH,ABCB4,ABCG2,ABTB2,ACAT2,ACLY,ACSM5,ADH4,AHNAK,AKAP11,AKAP9,ALG13,ANKRD12,ANP32A,BAP1,BRD8,C9orf152,CAMKK2,CAPN2,CBLB,CCDC66,CDH1,CDH2,CHD6,COL5A3,CUL4B,CYLD,CYP4F2,DCTN1,DHX9,DIAPH2,DMD,DOCK4,DPP4,E2F5,ECHDC3,EGR1,EIF1AX,ERBB3,ESRRA,EXOC6,F11,F3,FGA,FGFR2,FMO1,FTSJ3,GAS2L3,GCLC,GOLGB1,GRHPR,HERC4,HMGCR,HNRNPC,HRAS,IL18,IRF6,JAK2,KDM6A,KEAP1,KIAA0100,KLF12,KLF9,LGR5,LIN7A,LMNA,LRIG1,LSM14B,LSS,MAGI3,MIA2,MKNK2,MPDZ,MRPL24,MRPS27,MYO1B,MYO5B,NAGK,NCOA1,NGEF,NINJ1,NI	139

					PBL,NPC1,NR5A2,PAN2,PAQR9,PCDH1,PDGFRB,PHKA2,PIK3CA,PKHD1,PLXNA2,POR,PPP1R9A,PPP2R1A,PRPF6,PRPS1,PSMD1,PSMD11,PSMF1,PXMP4,RAB11FIP2,RB1CC1,RBBP5,RHOD,RPA1,SBF2,SCAPER,SEC63,SERPINE2,SERPINH1,SETDB2,SHMT1,SHPRH,SLC20A2,SLC2A5,SLC2A9,SLC35B1,SLC9A3R1,SNX10,SPTBN1,ST3GAL4,STARD7,SYNE1,TEP1,TEX2,TMEM126A,TNRC6A,TOP1MT,TRIB3,TUBB6,TUBGCP2,TXNL1,UGP2,UROD,UTRN,WNT5B,ZFAND2B,ZNF654	
Cancer,Organismal Injury And Abnormalities	Tumorigenesis	Tumorigenesis Of Tissue	6.34E-29	-1.156	AASDH,ABCB1,ABCB4,ABCG2,ABCG5,ABCG8,ABTB2,ACAD8,ACAT2,ACLY,ACOX2,ACSL3,ACSL4,ACSL5,ACSM1,ACSM5,ACSS2,ACTR6,ADH4,AFG3L2,AGPAT2,AHCY,AHNAK,AHSA1,AKAP11,AKAP9,AKR1D1,ALAS1,ALDH1L1,ALG13,AMDHD1,ANKRD12,ANP32A,ANXA7,ARF3,ARGLU1,ARHGAP18,ARHGAP21,ARHGAP6,ARIH2,ARL15,ARL4A,ARL6IP4,ARSG,ASAP2,ASB13,ATG3,ATG4D,ATP1A1,ATP1B1,ATP6V1D,ATXN2L,AUH,BAAT,BAP1,BDH1,BHMT,BHMT2,BID,BIK,BLMH,BRD8,BUD23,C11orf54,C9orf152,C9orf16,CA3,CAMKK2,CAPN2,CARS1,CBLB,CCBE1,CCDC66,CCT3,CCT4,CCT5,CCT6A,CCT7,CD2AP,CD47,CD9,CD99L2,CDA,CDC14B,CDH1,CDH2,CDO1,CEBPD,CENPV,CES1,CGN,CHCHD3,CHCHD4,CHD6,CHPT1,CHRAC1,CIB3,CITED2,CLDND1,CLK2,CLMN,CLOCK,CM	585

TM6,CMTM8,CNN3,C
NOT4,CNOT6,CNP,C
OL27A1,COL5A3,CO
RO1B,CPEB4,CPT1A
,CRIP2,CRLS1,CROT
,CS,CSRNP1,CTDSP
1,CTPS2,CTSH,CUL4
B,CXADR,CYB5B,CY
LD,CYP2U1,CYP39A
1,CYP4F2,CYP7A1,C
YTH1,DAZAP1,DCTN
1,DDC,DDX60,DEK,D
GKZ,DHX9,DIAPH2,D
ICER1,DIO1,DIPK2A,
DIXDC1,DMD,DMTF1
,DNAJB1,DNAJC5,D
OCK4,DPP4,DPP9,D
SP,DSTN,DTYMK,DU
SP19,DVL1,E2F5,EC
HDC1,ECHDC3,EEP
D1,EFHD2,EGR1,EIF
1AX,EIF4EBP1,ELF1,
ENDOG,ENPP1,ERB
B3,ESRRA,EXOC6,E
XOSC2,EXTL2,F11,F
2R,F3,FADS2,FAM10
2A,FAM118B,FAM16
0B1,FAM172A,FAM7
6B,FAM89A,FBXO31,
FBXO9,FECH,FGA,F
GB,FGFR2,FGFRL1,
FGG,FH,FKBP4,FLC
N,FMO1,FMO5,FOXK
1,FOXO3,FPGS,FTSJ
3,G6PD,GAS2L3,GC
AT,GCLC,GCNT2,GD
A,GNA12,GNG12,GO
LGB1,GPD2,GPN1,G
RHPR,GSDMD,GSK3
A,GSS,GSTM2,GTF2
A2,GTF2F1,GTF3C2,
GYG1,HECA,HECTD
3,HERC4,HLF,HMG2
0B,HMGCR,HNRNPC
,HNRNPD,HOMER2,
HOOK1,HRAS,HSD1
7B2,HSD3B7,HSPB1,
ICK,ID2,ID4,IGFBP2,I
L18,ING4,IRF6,IRS1,I
TPA,ITPK1,JAK2,JMJ
D1C,KAT2B,KCMF1,
KDM6A,KEAP1,KHK,
KIAA0100,KITLG,KLF
12,KLF9,KLHDC2,KL
HL21,KLHL7,KPNB1,
KTN1,KYNU,L2HGD
H,LARS2,LGALS4,LG
R5,LIN7A,LIN7C,LITA
F,LMNA,LPIN1,LRIG1
,LRP12,LRRC28,LRR
C3,LSM14B,LSS,LTB
R,MACROH2A1,MAG

ED1,MAGI3,MAP1LC
3B,MAP2K3,MAP3K7
,MBNL2,MCOLN1,ME
F2A,MIA2,MKNK2,ML
LT10,MLX,MMD,MOC
OS,MON2,MPDZ,MP
P1,MPRIP,MRNIP,M
RPL12,MRPL20,MRP
L24,MRPL44,MRPS2
2,MRPS25,MRPS27,
MRPS9,MT1F,MYO1
B,MYO5B,MYO6,N4B
P2,N4BP2L1,NAGK,N
CALD,NCOA1,NDUF
A12,NDUFA9,NDUFB
9,NEK7,NET1,NFKBI
A,NGEF,NHLRC2,NI
NJ1,NIPBL,NME1,NO
SIP,NPC1,NR1H4,NR
2F6,NR3C2,NR5A2,N
RIP1,NT5C,NUDT1,N
UDT19,NUDT7,NUP6
2,NUP88,NUS1,OAZ1
,OSBPL1A,OTUD6B,
OXR1,PALMD,PAN2,
PAN3,PAQR9,PCDH
1,PCGF5,PDCD2,PD
E2A,PDE9A,PDGFC,
PDGFRB,PDLIM1,PD
P2,PDPK1,PDRG1,P
ER1,PER3,PES1,PE
X26,PEX6,PFDN2,PF
KFB1,PGAP1,PHF20
L1,PHKA2,PIK3AP1,
PIK3CA,PKHD1,PLD
1,PLXNA2,PMPCA,P
NKD,PNRC1,POLE4,
POR,PPIF,PPM1A,P
POX,PPP1R12A,PPP
1R14B,PPP1R1B,PP
P1R3B,PPP1R9A,PP
P2R1A,PRDX6,PRPF
19,PRPF39,PRPF6,P
RPS1,PRPS2,PSEN2
,PSMA6,PSMD1,PSM
D11,PSMD2,PSMD8,
PSME2,PSMG1,PSP
C1,PSPH,PTBP1,PT
BP2,PXMP4,QDPR,R
AB11FIP2,RAB1B,RA
BEPK,RABGAP1L,RA
I14,RALGPS2,RAN,R
ASA3,RASSF3,RB1C
C1,RBBP5,RBBP7,R
BKS,RCAN1,RCL1,R
EPIN1,RGS2,RHBDD
1,RHOD,RICTOR,RN
F14,RNF144A,RNF16
7,ROCK1,RPA1,RPIA
,RUFY3,SAA4,SAR1
B,SARS1,SBF2,SCA
PER,SCARB2,SCYL1

					,SDC4,SDR42E1,SEC63,SECISBP2L,SENP6,SERPINA6,SERPINE2,SERPINH1,SESN3,SETDB2,SFRP5,SGK2,SGTB,SH3D19,SHMT1,SHPRH,SIRT5,SLC10A1,SLC16A5,SLC1A4,SLC20A1,SLC20A2,SLC25A32,SLC27A2,SLC2A2,SLC2A5,SLC2A9,SLC30A10,SLC35A3,SLC35B1,SLC35B3,SLC38A2,SLC39A10,SLC39A8,SLC6A12,SLC9A3R1,SLK,SMAD7,SMAP1,SNAPC2,SNRNP200,SNTB1,SNX10,SNX5,SNX9,SOAT2,SOD2,SPARC,SPG21,SPON2,SPOP,SPTBN1,SPTBN2,ST3GAL4,STARD7,STIP1,STK24,STK40,STOM,STT3B,STX18,STX8,STXBP3,SULF2,SUMF1,SYAP1,SYNE1,SYT1,TBC1D16,TBC1D20,TBC1D24,TBRG4,TCIM,TCP1,TEP1,TEX2,THAP4,THRSP,TIFA,TIGD2,TIMM10,TIMM44,TK1,TLCD4,TLE1,TMBIM1,TMEM126A,TMEM219,TMEM41A,TMEM41B,TMEM51,TMEM62,TMEM97,TNFAIP2,TNFAIP8L1,TNRC6A,TOP1MT,TPI1,TPR,TPRG1L,TPRKB,TRIB3,TRMT1,TSN,TSPAN33,TSTA3,TTC1,TTC19,TUBB,TUBB6,TUBGCP2,TXN2,TXNL1,UBAC1,UBE2F,UBE2L3,UBR1,UBTF,UBXN1,UBXN6,UCK1,UGP2,UGT2B10,ULK1,UNG,UPP2,UROC1,UROD,USP47,UTP14A,UTRN,UXS1,VPS13A,WDR18,WDR26,WDR81,WIPI2,WNT5B,YPEL3,YWHAH,ZCCHC24,ZFAND2B,ZKSCAN1,ZNF23,ZNF32,ZNF326,ZNF654	
Gastrointestinal Disease,Organismal Injury And Abnormalities	Colorectal Disorder	Colorectal Disorder	3.45E-07	-1.159	AASDH,ABCB1,ABCG2,ABCG5,ACLY,ACOX2,ACSL5,ACSS2,ACTR6,AFG3L2,AGP	278

AT2,AHCY,AHNAK,A
KAP9,ALAS1,ALDH1
L1,ALG13,ANXA7,AR
HGAP21,ARL6IP4,AS
AP2,ASB13,ATG3,AT
G4D,ATP1B1,ATXN2
L,BAAT,BAP1,BID,C1
1orf54,C9orf152,C9or
f16,CARS1,CCDC66,
CCT4,CCT5,CCT7,C
D2AP,CD47,CD9,CD
99L2,CDH1,CDH2,C
DO1,CEBPD,CES1,C
GN,CHCHD3,CHD6,
CHPT1,CHRAC1,CL
DND1,CLK2,CLMN,C
LOCK,CMTM6,CNOT
4,CNP,COL27A1,CO
L5A3,CPEB4,CPT1A,
CS,CTDSP1,CTPS2,
CUL4B,CYLD,CYP4F
2,DCTN1,DDX60,DG
KZ,DHX9,DICER1,D
MD,DMTF1,DOCK4,D
STN,EGR1,EIF4EBP
1,ELF1,ERBB3,ESRR
A,F2R,F3,FAM160B1,
FECH,FGA,FGB,FGF
R2,FGG,FLCN,FMO1
,FMO5,FOXO3,FPGS
,G6PD,GAS2L3,GNA
12,GNG12,GSS,GTF
2F1,HECA,HECTD3,
HMGCR,HNRNPC,H
RAS,HSD17B2,HSPB
1,ICK,ID2,IGFBP2,IL1
8,ING4,IRF6,IRS1,JA
K2,JMJD1C,KAT2B,K
DM6A,KEAP1,KIAA0
100,KITLG,KLF12,KL
HL7,KPNB1,KTN1,LG
ALS4,LGR5,LIN7A,L
RIG1,LRP12,LRRC3,
LTBR,MACROH2A1,
MAGED1,MAP3K7,M
EF2A,MIA2,MKNK2,
MLLT10,MLX,MON2,
MPDZ,MPRIP,MRNIP
,MRPS9,MT1F,MYO5
B,MYO6,NCOA1,NEK
7,NET1,NFKBIA,NGE
F,NHLRC2,NIPBL,NM
E1,NOSIP,NR1H4,NR
3C2,NR5A2,NT5C,N
UDT19,NUDT7,NUP6
2,OAZ1,OSBPL1A,PA
QR9,PCDH1,PCGF5,
PDE9A,PDGFRB,PD
LIM1,PDP2,PDPK1,P
DRG1,PER1,PFDN2,
PGAP1,PHF20L1,PH
KA2,PIK3AP1,PIK3C

					A,PKHD1,PLD1,PLXNA2,PNKD,PNRC1,POR,PPP1R14B,PPP1R1B,PPP1R9A,PPP2R1A,PRDX6,PRPF39,PRPF6,PSMD8,PTBP1,QDPR,RABGAP1L,RASSF3,RB1CC1,RBBP5,RICTOR,ROCK1,RPA1,RUFY3,SAA4,SBF2,SCAPER,SCYL1,SEC63,SECISBP2L,SENP6,SERPINE2,SESN3,SGK2,SHPRH,SLC1A4,SLC20A1,SLC20A2,SLC27A2,SLC2A2,SLC2A5,SLC30A10,SLC35B3,SLC38A2,SLC39A10,SLC39A8,SLC6A12,SMAD7,SMAP1,SNRNP200,SNX9,SOAT2,SOD2,SPARC,SPOP,SPTBN1,SPTBN2,STIP1,STX8,SULF2,SYAP1,SYNE1,SYT1,TBC1D16,TBC1D24,TBRG4,TCP1,TEP1,TIMM44,TK1,TMEM126A,TMEM41B,TMEM97,TNRC6A,TOP1MT,TPI1,TPR,TRIB3,TSTA3,TTC1,TUBB,UBAC1,UBTF,UGP2,ULK1,UNG,UROC1,UTRN,VPS13A,WDR18,WDR26,WDR81,WNT5B,ZCCHC24,ZNF23	
Cell-To-Cell Signaling And Interaction	Binding	Binding Of Tumor Cell Lines	5.02E-04	-1.215	ARHGAP21,CD47,CD9,CDH1,CDH2,CXADR,CYTH1,DPP4,DSP,EGR1,ERBB3,F2R,F3,FGA,FGFR2,HRAS,IL18,IRS1,JAK2,LGALS4,LTBR,MYO6,NET1,NFKBIA,NME1,NR1H4,PLD1,RCAN1,TCIM,UTRN	30
Cancer,Organismal Injury And Abnormalities	Adenoma Formation	Adenoma	1.35E-05	-1.226	ABCB1,ABCG2,ATP1A1,BHMT,BID,C9orf16,CDH1,CDH2,CEBPD,CLMN,EGR1,EIF4EBP1,ERBB3,FGFR2,FOXO3,GSK3A,HRAS,ICK,ID4,LRIG1,NR1H4,NR3C2,NR5A2,NRIP1,NUDT1,OAZ1,OSBPL1A,PIK3CA,PRDX6,SLC30A10,SPOP,SPTBN1,TRIB3,UGP2,UNG	35
Cancer,Organismal	Nonhematologi	Nonhematologi	1.38E-31	-1.303	AASDH,ABCB1,ABC	592

Injury And Abnormalities	c Malignant Neoplasm	c Malignant Neoplasm	B4,ABCG2,ABCG5,ABCG8,ABTB2,ACAD8,ACAT2,ACLY,ACOX2,ACSL3,ACSL4,ACSL5,ACSM1,ACSM5,ACSS2,ACTR6,ADH4,AFG3L2,AGPAT2,AHCY,AHNAK,AHSA1,AKAP11,AKAP9,AKR1D1,ALAS1,ALDH1L1,ALG13,AMDHD1,ANKRD12,ANP32A,ANXA7,ARF3,ARGLU1,ARHGAP18,ARHGAP21,ARHGAP6,ARIH2,ARL15,ARL4A,ARL6IP4,ARSG,ASAP2,ASB13,ATG3,ATG4D,ATP1A1,ATP1B1,ATP6V1D,ATXN2L,AUH,BAAT,BAP1,BDH1,BHMT,BHMT2,BID,BIK,BLMH,BRD8,BUD23,C11orf54,C9orf152,C9orf16,CA3,CAMKK2,CAPN2,CARS1,CBLB,CCBE1,CCDC66,CCT3,CCT4,CCT5,CCT6A,CCT7,CD2AP,CD47,CD9,CD99L2,CDA,CDC14B,CDH1,CDH2,CDO1,CEBPD,CENPV,CES1,CGN,CHCHD3,CHCHD4,CHD6,CHPT1,CHRAC1,CIB3,CITED2,CLDND1,CLK2,CLMN,CLOCK,CLTA,CMTM6,CMTM8,CNN3,CNOT4,CNOT6,CNP,COL27A1,COL5A3,CORO1B,CPEB4,CPT1A,CRIP2,CRLS1,CROT,CS,CSRNP1,CTDSP1,CTPS2,CTSH,CUL4B,CXADR,CYB5B,CYLD,CYP2U1,CYP39A1,CYP4F2,CYP7A1,CYTH1,DAZAP1,DCTN1,DDC,DDX60,DEK,DGKZ,DHX9,DIAPH2,DICER1,DIO1,DIPK2A,DIXDC1,DMD,DMTF1,DNAJB1,DNAJC5,DOCK4,DPP4,DPP9,DSP,DSTN,DTYMK,DUSP19,DVL1,E2F5,ECHDC1,ECHDC3,EEPD1,EFHD2,EGR1,EIF1AX,EIF4EBP1,ELF1,ENDOG,ENPP1,ERBB3,ESRRA,EXOC6,EXOSC2,EX

TL2,F11,F2R,F3,FAD
S2,FAM102A,FAM11
8B,FAM160B1,FAM1
72A,FAM76B,FAM89
A,FBXO31,FBXO9,FE
CH,FGA,FGB,FGFR2
,FGFRL1,FGG,FH,FK
BP4,FLCN,FMO1,FM
O5,FOXK1,FOXO3,F
PGS,FTSJ3,G6PD,G
ABARAP,GAS2L3,GC
AT,GCLC,GCNT2,GD
A,GNA12,GNG12,GO
LGB1,GPD2,GPN1,G
RHPR,GSDMD,GSK3
A,GSS,GSTM2,GTF2
A2,GTF2F1,GTF3C2,
GYG1,HECA,HECTD
3,HERC4,HLF,HMG2
0B,HMGCR,HNRNPC
,HNRNPD,HOMER2,
HOOK1,HRAS,HSD1
7B2,HSD3B7,HSPB1,
ICK,ID2,ID4,IGFBP2,I
L18,ING4,IRF6,IRS1,I
TPA,ITPK1,JAK2,JMJ
D1C,KAT2B,KCMF1,
KDM6A,KEAP1,KHK,
KIAA0100,KITLG,KLF
12,KLF9,KLHDC2,KL
HL21,KLHL7,KPNB1,
KTN1,KYNU,L2HGD
H,LARS2,LGALS4,LG
R5,LIN7A,LIN7C,LITA
F,LMNA,LPIN1,LRIG1
,LRP12,LRRC28,LRR
C3,LSM14B,LSS,LTB
R,MACROH2A1,MAG
ED1,MAGI3,MAP1LC
3B,MAP2K3,MAP3K7
,MBNL2,MCOLN1,ME
F2A,MIA2,MKNK2,ML
LT10,MLX,MMD,MOC
OS,MON2,MPDZ,MP
P1,MPRIP,MRNIP,M
RPL12,MRPL20,MRP
L24,MRPL44,MRPS2
2,MRPS25,MRPS27,
MRPS9,MT1F,MYO1
B,MYO5B,MYO6,N4B
P2,N4BP2L1,NAGK,N
CALD,NCOA1,NDUF
A12,NDUFA9,NDUFB
9,NEK7,NET1,NFKBI
A,NGEF,NHLRC2,NI
CN1,NINJ1,NIPBL,N
ME1,NOP10,NOSIP,
NPC1,NR1H4,NR2F6
,NR3C2,NR5A2,NRIP
1,NT5C,NUDT1,NUD
T19,NUDT7,NUP62,N
UP88,NUS1,OAZ1,O

SBPL1A,OTUD6B,OX
R1,PALMD,PAN2,PA
N3,PAQR9,PCDH1,P
CGF5,PDCD2,PDE2A
,PDE9A,PDGFC,PDG
FRB,PDLIM1,PDP2,P
DPK1,PDRG1,PER1,
PER3,PES1,PEX26,P
EX6,PFDN2,PFKFB1,
PGAP1,PHF20L1,PH
KA2,PIK3AP1,PIK3C
A,PKHD1,PLD1,PLX
NA2,PMPCA,PNKD,P
NRC1,POR,PPIF,PP
M1A,PPOX,PPP1R12
A,PPP1R14B,PPP1R
1B,PPP1R3B,PPP1R
9A,PPP2R1A,PRDX6,
PRPF19,PRPF39,PR
PF6,PRPS1,PRPS2,P
SEN2,PSMA6,PSMD
1,PSMD11,PSMD2,P
SMD8,PSME2,PSMG
1,PSPC1,PSPH,PTB
P1,PTBP2,PXMP4,Q
DPR,RAB11FIP2,RA
B1B,RABEPK,RABG
AP1L,RAI14,RALGPS
2,RAN,RASA3,RASS
F3,RB1CC1,RBBP5,
RBBP7,RBKS,RCAN
1,RCL1,REPIN1,RGS
2,RHBDD1,RHOD,RI
CTOR,RNF14,RNF14
4A,RNF167,ROCK1,
RPA1,RPIA,RUFY3,S
AA4,SAR1B,SARS1,
SBF2,SCAPER,SCA
RB2,SCGN,SCOC,S
CYL1,SDC4,SDR42E
1,SEC63,SECISBP2L
,SENP6,SERPINA6,S
ERPINE2,SERPINH1,
SESN3,SETDB2,SFR
P5,SGK2,SGTB,SH3
BGRL2,SH3D19,SHM
T1,SHPRH,SIRT5,SL
C10A1,SLC16A5,SLC
1A4,SLC20A1,SLC20
A2,SLC25A32,SLC27
A2,SLC2A2,SLC2A5,
SLC2A9,SLC30A10,S
LC35A3,SLC35B1,SL
C35B3,SLC38A2,SLC
39A10,SLC39A8,SLC
6A12,SLC9A3R1,SLK
,SMAD7,SMAP1,SNA
PC2,SNRNP200,SNT
B1,SNX10,SNX5,SNX
9,SOAT2,SOD2,SPA
RC,SPG21,SPON2,S
POP,SPTBN1,SPTBN

					2,ST3GAL4,STARD4,STARD7,STIP1,STK24,STK40,STOM,STT3B,STX18,STX8,STXBP3,SULF2,SUMF1,SYAP1,SYNE1,SYT1,TBC1D16,TBC1D20,TBC1D24,TBRG4,TCIM,TCP1,TEP1,TEX2,THAP4,THRSP,TIFA,TIGD2,TIMM10,TIMM44,TK1,TLCD4,TLE1,TMBIM1,TMEM126A,TMEM219,TMEM41A,TMEM41B,TMEM51,TMEM62,TMEM97,TNFAIP2,TNFAIP8L1,TNRC6A,TOP1MT,TPI1,TPR,TPRG1L,TPRKB,TRIB3,TRMT1,TSN,TSPAN33,TSTA3,TTC1,TTC19,TUBB,TUBB6,TUBGCP2,TXN2,TXNL1,UBAC1,UBE2F,UBE2L3,UBR1,UBTF,UBXN1,UBXN6,UCK1,UGP2,UGT2B10,ULK1,UNG,UPP2,UROC1,UROD,USP47,UTP14A,UTRN,UXS1,VPS13A,WDR18,WDR26,WDR81,WIPI2,WNT5B,YPEL3,YWHAH,ZCCHC24,ZFAND2B,ZKSCAN1,ZNF23,ZNF32,ZNF326,ZNF654	
Cancer,Organismal Injury And Abnormalities	Pelvic Tumor	Pelvic Tumor	1.54E-09	-1.311	ABCB1,ABCB4,ABCG2,ABCG5,ACAD8,ACLY,ACOX2,ACSL3,ACSL4,ACSM1,ACSS2,AFG3L2,AGPAT2,AHNAK,AKAP11,AKAP9,ALAS1,ALDH1L1,ALG13,ANKRD12,ARHGAP18,ARHGAP21,ARHGAP6,ARIH2,ARL15,ASAP2,ATP1A1,ATP6V1D,ATXN2L,AUH,BAAT,BAP1,BDH1,BIK,BRD8,C11orf54,CA3,CAPN2,CARS1,CBLB,CCDC66,CCT5,CCT6A,CD2AP,CD47,CD99L2,CDA,CDH1,CDH2,CDO1,CES1,CGN,CHD6,CLDND1,CLK2,CLMN,CMTM8,CNOT4,CNOT6,CNP,COL5A3,CPEB4,CPT1A,CROT,CS,CSRNP1,CTSH,CXADR,CYP4F2,CYP7A1,CYTH1,	348

DAZAP1,DCTN1,DD
C,DDX60,DGKZ,DHX
9,DIAPH2,DICER1,DI
O1,DIPK2A,DIXDC1,
DMD,DOCK4,DPP4,D
PP9,DSP,DSTN,DVL
1,EFHD2,EGR1,EIF1
AX,EIF4EBP1,ELF1,E
NPP1,ERBB3,ESRRA
,EXOC6,F11,F2R,F3,
FAM160B1,FAM172A
,FAM89A,FECH,FGA,
FGFR2,FGFRL1,FGG
,FH,FKBP4,FLCN,FO
XK1,FOXO3,GDA,GN
A12,GOLGB1,GPD2,
GRHPR,GSDMD,GS
K3A,GSTM2,GTF2A2
,GTF2F1,GYG1,HEC
A,HECTD3,HLF,HMG
CR,HRAS,HSD3B7,H
SPB1,ICK,ID2,IGFBP
2,IL18,ING4,IRF6,IRS
1,ITPA,JAK2,JMJD1C
,KAT2B,KCMF1,KDM
6A,KEAP1,KIAA0100,
KLF9,KLHDC2,KTN1,
KYNU,L2HGDH,LAR
S2,LGALS4,LGR5,LI
N7C,LITAF,LMNA,LPI
N1,LRIG1,LRP12,LR
RC28,LSM14B,LSS,L
TBR,MACROH2A1,M
AGED1,MAGI3,MBNL
2,MCOLN1,MEF2A,M
IA2,MKNK2,MLLT10,
MLX,MMD,MOCOS,M
ON2,MPDZ,MPRIP,M
RPL44,MRPS22,MRP
S25,MRPS27,MYO1B
,MYO5B,MYO6,N4BP
2,NAGK,NCOA1,NDU
FA9,NEK7,NFKBIA,N
HLRC2,NINJ1,NIPBL,
NME1,NOSIP,NPC1,
NR2F6,NR3C2,NR5A
2,NRIP1,NUDT7,NUP
62,NUP88,OAZ1,OSB
PL1A,OTUD6B,OXR1
,PALMD,PAN3,PCDH
1,PCGF5,PDCD2,PD
E2A,PDE9A,PDGFRB
,PDLIM1,PDP2,PDPK
1,PDRG1,PER1,PER
3,PEX6,PGAP1,PHF2
0L1,PHKA2,PIK3CA,
PKHD1,PLD1,PLXNA
2,PNRC1,POR,PPIF,
PPP1R1B,PPP1R3B,
PPP1R9A,PPP2R1A,
PRPF6,PSEN2,PSMA
6,PSMD1,PSMD2,PT

					BP1,PTBP2,PXMP4,RAB11FIP2,RABEPK,RABGAP1L,RALGPS2,RB1CC1,RBBP5,RBBP7,RCAN1,RCL1,REPIN1,RGS2,RHOD,RICTOR,RNF144A,ROCK1,RPIA,SBF2,SCAPER,SCARB2,SCYL1,SDC4,SDR42E1,SEC63,SECISBP2L,SENP6,SERPINA6,SERPINE2,SESN3,SETDB2,SHPRH,SIRT5,SLC16A5,SLC1A4,SLC20A1,SLC20A2,SLC25A32,SLC2A2,SLC2A5,SLC38A2,SLC39A10,SLC6A12,SLK,SMAP1,SNRNP200,SNX10,SOD2,SPARC,SPOP,SPTBN1,SPTBN2,ST3GAL4,STARD7,STK24,STK40,STOM,STT3B,STX8,STXBP3,SULF2,SYNE1,TBC1D16,TBC1D20,TBC1D24,TEP1,TEX2,TIGD2,TK1,TLE1,TMEM126A,TNFAIP2,TNRC6A,TOP1MT,TPI1,TPR,TPRG1L,TRIB3,TRMT1,TSPAN33,TTC1,TUBB,TXNL1,UBAC1,UBE2F,UBR1,UGP2,UGT2B10,UROC1,UROD,USP47,UTRN,UXS1,VPS13A,WIPI2,WNT5B,YPEL3,ZCCHC24,ZFAND2B,ZKSCAN1,ZNF23,ZNF32,ZNF654	
Cancer,Cellular Development,Cellular Growth And Proliferation,Organismal Injury And Abnormalities,Tumor Morphology	Proliferation	Proliferation Of Cancer Cells	1.18E-03	-1.325	ABCG2,AHCY,BIK,CDH1,DICER1,DOCK4,EGR1,EIF4EBP1,ERBB3,F2R,FOXO3,HMGCR,HRAS,ID2,IL18,IRS1,KITLG,LITAF,PDPK1,PLD1,RB1CC1,RCAN1,TLE1,ULK1	24
Cell Cycle,Cell-To-Cell Signaling And Interaction,Cellular Growth And Proliferation	Contact Growth Inhibition	Contact Growth Inhibition	8.28E-04	-1.345	ABCG2,CDH1,EGR1,ERBB3,FOXO3,HRAS,ING4,JAK2,NME1,PER1,PRKAR2A,RHOD,SMAD7,TIFA	14
Cancer,Organismal Injury And Abnormalities	Development	Development Of Malignant Tumor	3.96E-11	-1.356	AASDH,ABCB1,ABCB4,ABCG5,ACAD8,ACLY,ACOX2,ACSL3,ACSL4,ACSM1,ACSS2,ACTR6,AFG3L2,AGPAT2,AHNAK,AKAP11,AKAP9,ALAS1,ALD	386

H1L1,ALG13,ANKRD
12,ANP32A,ANXA7,A
RHGAP21,ARHGAP6
,ARIH2,ARL15,ARL6I
P4,ASAP2,ASB13,AT
G3,ATG4D,ATXN2L,
AUH,BAAT,BAP1,BD
H1,BHMT,BRD8,BUD
23,C11orf54,C9orf152
,CAPN2,CARS1,CBL
B,CCDC66,CCT3,CC
T5,CCT6A,CCT7,CD2
AP,CD47,CD9,CD99L
2,CDC14B,CDH1,CD
H2,CDO1,CES1,CGN
,CHCHD3,CHD6,CHP
T1,CHRAC1,CLDND1
,CLK2,CLMN,CLOCK,
CMTM6,CMTM8,CNO
T4,CNOT6,CNP,COL
27A1,COL5A3,CPEB
4,CPT1A,CS,CTDSP
1,CTPS2,CUL4B,CXA
DR,CYLD,CYP4F2,C
YP7A1,DAZAP1,DCT
N1,DDX60,DEK,DGK
Z,DHX9,DIAPH2,DIC
ER1,DIO1,DIPK2A,DI
XDC1,DMD,DMTF1,D
OCK4,DPP4,DPP9,D
SP,DSTN,DUSP19,E
2F5,EGR1,EIF1AX,E
LF1,ERBB3,ESRRA,
EXOC6,EXTL2,F11,F
2R,F3,FAM102A,FAM
160B1,FAM172A,FEC
H,FGA,FGB,FGFR2,F
GG,FH,FLCN,FMO1,
FMO5,FOXO3,FTSJ3
,G6PD,GAS2L3,GNA
12,GNG12,GOLGB1,
GPD2,GRHPR,GSK3
A,GSS,GSTM2,GTF2
F1,GTF3C2,GYG1,H
ECA,HECTD3,HERC
4,HMGCR,HNRNPC,
HRAS,HSPB1,ICK,ID
2,IGFBP2,IL18,ING4,I
RF6,IRS1,ITPA,JAK2,
JMJD1C,KAT2B,KCM
F1,KDM6A,KEAP1,KI
AA0100,KITLG,KLF1
2,KLHL7,KPNB1,KTN
1,KYNU,L2HGDH,LG
R5,LIN7A,LMNA,LRI
G1,LRP12,LRRC28,L
RRC3,LSM14B,LTBR
,MAGED1,MAGI3,MA
P2K3,MAP3K7,MBNL
2,MCOLN1,MEF2A,M
IA2,MKNK2,MLLT10,
MLX,MOCOS,MON2,

MPDZ,MPP1,MPRIP,
MRNIP,MRPL44,MRP
S22,MRPS27,MRPS9
,MYO1B,MYO5B,MY
O6,N4BP2L1,NCOA1,
NDUFA9,NEK7,NET1
,NFKBIA,NGEF,NHL
RC2,NINJ1,NIPBL,N
ME1,NOSIP,NPC1,N
R1H4,NR2F6,NR3C2,
NRIP1,NUDT1,NUDT
19,NUDT7,NUP62,N
UP88,OAZ1,OSBPL1
A,OXR1,PALMD,PAN
2,PAN3,PAQR9,PCD
H1,PCGF5,PDCD2,P
DE2A,PDE9A,PDGF
C,PDGFRB,PDLIM1,
PDP2,PDPK1,PER1,
PER3,PFDN2,PGAP1
,PHF20L1,PHKA2,PI
K3AP1,PIK3CA,PKH
D1,PLD1,PLXNA2,P
MPCA,PNKD,PNRC1,
POR,PPP1R14B,PPP
1R1B,PPP1R9A,PPP
2R1A,PRPF19,PRPF
39,PRPF6,PSMD1,P
SMD2,PSMD8,PTBP
1,PTBP2,QDPR,RAB
EPK,RABGAP1L,RAI
14,RALGPS2,RASSF
3,RB1CC1,RBBP5,R
BBP7,RCL1,RHBDD1
,RICTOR,ROCK1,RP
A1,RPIA,RUFY3,SAA
4,SARS1,SBF2,SCAP
ER,SCARB2,SCYL1,
SDC4,SEC63,SECIS
BP2L,SENP6,SERPI
NE2,SERPINH1,SES
N3,SETDB2,SGTB,S
HPRH,SLC1A4,SLC2
0A1,SLC20A2,SLC2A
2,SLC2A9,SLC35B3,
SLC38A2,SLC39A10,
SLC39A8,SLC6A12,S
LC9A3R1,SMAD7,SM
AP1,SNRNP200,SNT
B1,SNX10,SNX9,SO
AT2,SOD2,SPARC,S
PON2,SPOP,SPTBN
1,SPTBN2,STARD7,S
TIP1,STK24,STK40,S
TOM,STT3B,STX8,S
TXBP3,SULF2,SYAP
1,SYNE1,SYT1,TBC1
D16,TBC1D20,TBC1
D24,TBRG4,TCP1,TE
P1,TEX2,TIGD2,TIM
M44,TK1,TLCD4,TLE
1,TMEM126A,TMEM4

					1B,TMEM51,TMEM97,TNFAIP2,TNRC6A,TOP1MT,TPI1,TPR,TPRG1L,TRIB3,TTC1,TUBB,TUBB6,TXNL1,UBAC1,UBE2F,UBTF,UGP2,UGT2B10,ULK1,UNG,UROC1,UTP14A,UTRN,UXS1,VPS13A,WDR18,WDR26,WDR81,WIPI2,WNT5B,ZCCHC24,ZFAND2B,ZKSCAN1,ZNF32	
Carbohydrate Metabolism,Molecular Transport	Transport	Transport Of Carbohydrate	1.45E-07	-1.357	ABCB1,ABCB4,DGKZ,ENPP1,ERBB3,GSK3A,IRS1,MAP2K3,MEF2A,NPC1,OGA,PDGFRB,PDPK1,PGAP1,PIK3CA,PPP1R3B,REPIN1,ROCK1,SLC2A2,SLC2A5,SLC2A9,SLC35A3,STXBP3,TRIB3	24
Lipid Metabolism,Small Molecule Biochemistry,Vitamin And Mineral Metabolism	Metabolism	Steroid Metabolism	1.56E-05	-1.362	ABCG5,ABCG8,ACAT2,ACLY,AKR1D1,BAAT,CES1,CYP39A1,CYP7A1,ELOVL6,FECH,G6PD,HMGCR,HSD17B2,HSD3B7,KPNB1,LSS,NPC1,NR1H4,NR5A2,POR,RAN,SERPINA6,SOAT2,YWHAH	25
Cancer,Organismal Injury And Abnormalities,Reproductive System Disease	Genital Tumor	Genital Tumor	9.74E-10	-1.363	ABCB1,ABCB4,ABCG2,ABCG5,ACAD8,ACLY,ACOX2,ACSL3,ACSL4,ACSM1,ACSS2,AGPAT2,AHNAK,AKAP11,AKAP9,ALAS1,ALDH1L1,ALG13,ANKRD12,ARHGAP18,ARHGAP21,ARHGAP6,ARIH2,ARL15,ASAP2,ATP1A1,ATP6V1D,ATXN2L,AUH,BAAT,BAP1,BDH1,BIK,BLMH,BRD8,CA3,CAPN2,CBLB,CCT5,CCT6A,CD2AP,CD47,CD99L2,CDA,CDH1,CDH2,CDO1,CES1,CGN,CHD6,CLDND1,CLMN,CMTM8,CNOT4,CNOT6,CNP,COL5A3,CPEB4,CPT1A,CS,CSRNP1,CTSH,CXADR,CYP4F2,CYP7A1,CYTH1,DAZAP1,DCTN1,DDX60,DGKZ,DHX9,DIAPH2,DICER1,DIO1,DIPK2A,DIXDC1,DMD,DOCK4,DPP4,DPP	331

9,DSP,DSTN,DVL1,E
FHD2,EGR1,EIF1AX,
EIF4EBP1,ELF1,ENP
P1,ERBB3,EXOC6,F1
1,F2R,F3,FAM160B1,
FAM172A,FAM89A,F
ECH,FGA,FGFR2,FG
FRL1,FGG,FH,FKBP
4,FLCN,FOXK1,FOX
O3,GDA,GNA12,GOL
GB1,GPD2,GRHPR,
GSDMD,GSK3A,GST
M2,GTF2A2,GYG1,H
ECA,HECTD3,HLF,H
MGCR,HRAS,HSD3B
7,HSPB1,ICK,ID2,IGF
BP2,IL18,ING4,IRF6,I
RS1,ITPA,JAK2,JMJ
D1C,KAT2B,KCMF1,
KDM6A,KEAP1,KIAA
0100,KITLG,KLF9,KT
N1,L2HGDH,LARS2,L
GALS4,LGR5,LIN7C,
LITAF,LMNA,LRIG1,L
RP12,LRRC28,LSM1
4B,LSS,LTBR,MACR
OH2A1,MAGED1,MA
GI3,MBNL2,MCOLN1
,MEF2A,MIA2,MKNK
2,MLLT10,MLX,MMD,
MOCOS,MON2,MPD
Z,MPRIP,MRPL44,M
RPS22,MRPS25,MR
PS27,MYO1B,MYO5
B,MYO6,N4BP2,NCO
A1,NDUFA9,NEK7,N
FKBIA,NHLRC2,NIPB
L,NME1,NOSIP,NPC
1,NR2F6,NR3C2,NR5
A2,NRIP1,NUDT7,NU
P62,NUP88,OAZ1,OS
BPL1A,OTUD6B,OXR
1,PALMD,PAN3,PCD
H1,PCGF5,PDCD2,P
DE2A,PDGFRB,PDLI
M1,PDP2,PDPK1,PE
R1,PER3,PEX6,PGA
P1,PHF20L1,PHKA2,
PIK3CA,PKHD1,PLD
1,PLXNA2,PNRC1,P
OR,PPIF,PPP1R1B,P
PP1R3B,PPP1R9A,P
PP2R1A,PRPF6,PSE
N2,PSMA6,PSMD1,P
SMD2,PTBP1,PTBP2
,PXMP4,RAB11FIP2,
RABEPK,RABGAP1L,
RALGPS2,RB1CC1,R
BBP5,RBBP7,RCAN1
,RCL1,REPIN1,RGS2
,RHOD,RICTOR,RNF
144A,ROCK1,RPIA,S

					BF2,SCAPER,SCARB2,SCYL1,SDC4,SDR42E1,SECISBP2L,SERPINA6,SERPINE2,SESN3,SETDB2,SIRT5,SLC16A5,SLC1A4,SLC20A1,SLC20A2,SLC25A32,SLC2A2,SLC2A5,SLC38A2,SLC39A10,SLC6A12,SLK,SMAP1,SNRNP200,SNX10,SOD2,SPARC,SPOP,SPTBN1,SPTBN2,ST3GAL4,STARD7,STK24,STK40,STOM,STT3B,STX8,STXBP3,SULF2,SYNE1,TBC1D16,TBC1D20,TBC1D24,TEP1,TEX2,TIFA,TIGD2,TK1,TLE1,TMEM126A,TNFAIP2,TNRC6A,TOP1MT,TPI1,TPR,TPRG1L,TRIB3,TRMT1,TSPAN33,TTC1,TUBB,TXNL1,UBAC1,UBE2F,UBR1,UGP2,UGT2B10,UROC1,UROD,USP47,UTRN,UXS1,VPS13A,WNT5B,YPEL3,ZCCHC24,ZFAND2B,ZKSCAN1,ZNF23,ZNF32,ZNF654	
Cancer,Organismal Injury And Abnormalities	Tumorigenesis	Tumorigenesis Of Epithelial Neoplasm	4,60E-13	-1,377	AASDH,ABCB1,ABCB4,ABCG2,ABCG5,ACAD8,ACLY,ACOX2,ACSL3,ACSL4,ACSM1,ACSS2,ACTR6,AFG3L2,AGPAT2,AHNAK,AKAP11,AKAP9,ALAS1,ALDH1L1,ALG13,ANKRD12,ANP32A,ANXA7,ARHGAP21,ARHGAP6,ARIH2,ARL15,ARL6IP4,ASAP2,ASB13,ATG3,ATG4D,ATP1A1,ATXN2L,AUH,BAAT,BAP1,BDH1,BHMT,BID,BRD8,BUD23,C11orf54,C9orf152,C9orf16,CAPN2,CARS1,CBLB,CCDC66,CCT3,CCT5,CCT6A,CCT7,CD2AP,CD47,CD9,CD99L2,CDC14B,CDH1,CDH2,CDO1,CEBPD,CES1,CGN,CHCHD3,CHD6,CHPT1,CHRAC1,CLDND1,CLK2,CLMN,CLOCK,CMTM6,CMTM8,CNOT4,CNOT6,CNP,COL2	394

7A1,COL5A3,CPEB4,
CPT1A,CS,CTDSP1,
CTPS2,CUL4B,CXAD
R,CYLD,CYP4F2,CY
P7A1,DAZAP1,DCTN
1,DDX60,DEK,DGKZ,
DHX9,DIAPH2,DICE
R1,DIO1,DIPK2A,DIX
DC1,DMD,DMTF1,D
OCK4,DPP4,DPP9,D
SP,DSTN,DUSP19,E
2F5,EGR1,EIF1AX,EI
F4EBP1,ELF1,ERBB
3,ESRRA,EXOC6,EX
TL2,F11,F2R,F3,FAM
102A,FAM160B1,FA
M172A,FECH,FGA,F
GB,FGFR2,FGG,FH,
FLCN,FMO1,FMO5,F
OXO3,FTSJ3,G6PD,
GAS2L3,GNA12,GNG
12,GOLGB1,GPD2,G
RHPR,GSK3A,GSS,G
STM2,GTF2F1,GTF3
C2,GYG1,HECA,HEC
TD3,HERC4,HMGCR,
HNRNPC,HRAS,HSP
B1,ICK,ID4,IGFBP2,I
L18,ING4,IRF6,IRS1,I
TPA,JAK2,JMJD1C,K
AT2B,KCMF1,KDM6A
,KEAP1,KIAA0100,KI
TLG,KLF12,KLHL7,K
PNB1,KTN1,KYNU,L2
HGDH,LGR5,LIN7A,L
MNA,LRIG1,LRP12,L
RRC28,LRRC3,LSM1
4B,LTBR,MAGED1,M
AGI3,MAP2K3,MAP3
K7,MBNL2,MCOLN1,
MEF2A,MIA2,MKNK2
,MLLT10,MLX,MOCO
S,MON2,MPDZ,MPP
1,MPRIP,MRNIP,MR
PL44,MRPS22,MRPS
27,MRPS9,MYO1B,M
YO5B,MYO6,N4BP2L
1,NCOA1,NDUFA9,N
EK7,NET1,NFKBIA,N
GEF,NHLRC2,NIPBL,
NME1,NOSIP,NPC1,
NR1H4,NR2F6,NR3C
2,NR5A2,NRIP1,NUD
T1,NUDT19,NUDT7,
NUP62,NUP88,OAZ1,
OSBPL1A,OXR1,PAL
MD,PAN2,PAN3,PAQ
R9,PCDH1,PCGF5,P
DCD2,PDE2A,PDE9A
,PDGFC,PDGFRB,PD
LIM1,PDP2,PDPK1,P
ER1,PER3,PFDN2,P

					GAP1,PHF20L1,PHKA2,PIK3AP1,PIK3CA,PKHD1,PLD1,PLXNA2,PMPCA,PNKD,PNRC1,POR,PPP1R14B,PPP1R1B,PPP1R9A,PPP2R1A,PRDX6,PRPF19,PRPF39,PRPF6,PSMD1,PSMD2,PSMD8,PTBP1,PTBP2,QDPR,RABEPK,RABGAP1L,RAI14,RALGPS2,RASSF3,RB1CC1,RBBP5,RBBP7,RCL1,RHBDD1,RICTOR,ROCK1,RPA1,RPIA,RUFY3,SAA4,SARS1,SBF2,SCAPER,SCARB2,SCYL1,SDC4,SEC63,SECISBP2L,SENP6,SERPINE2,SERPINH1,SESN3,SETDB2,SGTB,SHPRH,SLC1A4,SLC20A1,SLC20A2,SLC2A2,SLC2A9,SLC30A10,SLC35B3,SLC38A2,SLC39A10,SLC39A8,SLC6A12,SLC9A3R1,SMAD7,SMAP1,SNRNP200,SNTB1,SNX10,SNX9,SOAT2,SOD2,SPARC,SPON2,SPOP,SPTBN1,SPTBN2,STARD7,STIP1,STK24,STK40,STOM,STT3B,STX8,STXBP3,SULF2,SYAP1,SYNE1,SYT1,TBC1D16,TBC1D20,TBC1D24,TBRG4,TCP1,TEP1,TEX2,TIGD2,TIMM44,TK1,TLCD4,TLE1,TMEM126A,TMEM41B,TMEM51,TMEM97,TNFAIP2,TNRC6A,TOP1MT,TPI1,TPR,TPRG1L,TRIB3,TTC1,TUBB,TUBB6,TXNL1,UBAC1,UBE2F,UBTF,UGP2,UGT2B10,ULK1,UNG,UROC1,UTP14A,UTRN,UXS1,VPS13A,WDR18,WDR26,WDR81,WIPI2,WNT5B,ZCCHC24,ZFAND2B,ZKSCAN1,ZNF32	
Lipid Metabolism,Molecular Transport,Small Molecule Biochemistry	Quantity	Quantity Of Steroid	1.05E-03	-1.391	ABCB1,ABCB4,ABCG5,ABCG8,ACAT2,AGPAT2,ATP1A1,BHMT,CES1,CLOCK,CYP7A1,DICER1,DIO1,EGR1,FKBP4,FMO5,F	33

					OXO3,GPD2,GSK3A,HMGCR,IL18,IRS1,LGR5,NCOA1,NPC1,NR1H4,NR5A2,POR,PSEN2,SERPINA6,SLC9A3R1,SOAT2,STARD4	
Cancer,Organismal Injury And Abnormalities	Frequency	Frequency Of Tumor	1.38E-12	-1.393	AASDH,ABCB1,ABCB4,ABCG2,ABCG5,ACAD8,ACLY,ACOX2,ACSL3,ACSL4,ACSM1,ACSS2,ACTR6,AFG3L2,AGPAT2,AHNAK,AKAP11,AKAP9,ALAS1,ALDH1L1,ALG13,ANKRD12,ANP32A,ANXA7,ARHGAP21,ARHGAP6,ARIH2,ARL15,ARL6IP4,ASAP2,ASB13,ATG3,ATG4D,ATP1A1,ATXN2L,AUH,BAAT,BAP1,BDH1,BHMT,BID,BRD8,BUD23,C11orf54,C9orf152,C9orf16,CAPN2,CARS1,CBLB,CCDC66,CCT3,CCT5,CCT6A,CCT7,CD2AP,CD47,CD9,CD99L2,CDC14B,CDH1,CDH2,CDO1,CEBPD,CES1,CGN,CHCHD3,CHD6,CHPT1,CHRAC1,CLDND1,CLK2,CLMN,CLOCK,CMTM6,CMTM8,CNOT4,CNOT6,CNP,COL27A1,COL5A3,CPEB4,CPT1A,CS,CTDSP1,CTPS2,CUL4B,CXADR,CYLD,CYP4F2,CYP7A1,DAZAP1,DCTN1,DDX60,DEK,DGKZ,DHX9,DIAPH2,DICER1,DIO1,DIPK2A,DIXDC1,DMD,DMTF1,DOCK4,DPP4,DPP9,DSP,DSTN,DUSP19,E2F5,EGR1,EIF1AX,EIF4EBP1,ELF1,ERBB3,ESRRA,EXOC6,EXTL2,F11,F2R,F3,FAM102A,FAM160B1,FAM172A,FECH,FGA,FGB,FGFR2,FGG,FH,FLCN,FMO1,FMO5,FOXO3,FTSJ3,G6PD,GAS2L3,GNA12,GNG12,GOLGB1,GPD2,GRHPR,GSK3A,GSS,GSTM2,GTF2F1,GTF3C2,GYG1,HECA,HECTD3,HERC4,HMGCR,	397

HNRNPC,HRAS,HSP
B1,ICK,ID2,ID4,IGFB
P2,IL18,ING4,IRF6,IR
S1,ITPA,JAK2,JMJD1
C,KAT2B,KCMF1,KD
M6A,KEAP1,KIAA010
0,KITLG,KLF12,KLHL
7,KPNB1,KTN1,KYN
U,L2HGDH,LGR5,LIN
7A,LMNA,LRIG1,LRP
12,LRRC28,LRRC3,L
SM14B,LTBR,MAGE
D1,MAGI3,MAP2K3,
MAP3K7,MBNL2,MC
OLN1,MEF2A,MIA2,
MKNK2,MLLT10,MLX
,MOCOS,MON2,MPD
Z,MPP1,MPRIP,MRNI
P,MRPL44,MRPS22,
MRPS27,MRPS9,MY
O1B,MYO5B,MYO6,N
4BP2L1,NCOA1,NDU
FA9,NEK7,NET1,NFK
BIA,NGEF,NHLRC2,
NINJ1,NIPBL,NME1,
NOSIP,NPC1,NR1H4,
NR2F6,NR3C2,NR5A
2,NRIP1,NUDT1,NUD
T19,NUDT7,NUP62,N
UP88,OAZ1,OSBPL1
A,OXR1,PALMD,PAN
2,PAN3,PAQR9,PCD
H1,PCGF5,PDCD2,P
DE2A,PDE9A,PDGF
C,PDGFRB,PDLIM1,
PDP2,PDPK1,PER1,
PER3,PFDN2,PGAP1
,PHF20L1,PHKA2,PI
K3AP1,PIK3CA,PKH
D1,PLD1,PLXNA2,P
MPCA,PNKD,PNRC1,
POR,PPP1R12A,PPP
1R14B,PPP1R1B,PP
P1R9A,PPP2R1A,PR
DX6,PRPF19,PRPF3
9,PRPF6,PSMD1,PS
MD2,PSMD8,PTBP1,
PTBP2,QDPR,RABE
PK,RABGAP1L,RAI1
4,RALGPS2,RASSF3,
RB1CC1,RBBP5,RBB
P7,RCL1,RHBDD1,RI
CTOR,ROCK1,RPA1,
RPIA,RUFY3,SAA4,S
ARS1,SBF2,SCAPER
,SCARB2,SCYL1,SD
C4,SEC63,SECISBP2
L,SENP6,SERPINE2,
SERPINH1,SESN3,S
ETDB2,SGTB,SHPR
H,SLC1A4,SLC20A1,
SLC20A2,SLC2A2,SL

					C2A9,SLC30A10,SLC35B3,SLC38A2,SLC39A10,SLC39A8,SLC6A12,SLC9A3R1,SMAD7,SMAP1,SNRNP200,SNTB1,SNX10,SNX9,SOAT2,SOD2,SPARC,SPON2,SPOP,SPTBN1,SPTBN2,STARD7,STIP1,STK24,STK40,STOM,STT3B,STX8,STXBP3,SULF2,SYAP1,SYNE1,SYT1,TBC1D16,TBC1D20,TBC1D24,TBRG4,TCP1,TEP1,TEX2,TIGD2,TIMM44,TK1,TLCD4,TLE1,TMEM126A,TMEM41B,TMEM51,TMEM97,TNFAIP2,TNRC6A,TOP1MT,TPI1,TPR,TPRG1L,TRIB3,TTC1,TUBB,TUBB6,TXNL1,UBAC1,UBE2F,UBTF,UGP2,UGT2B10,ULK1,UNG,UROC1,UTP14A,UTRN,UXS1,VPS13A,WDR18,WDR26,WDR81,WIPI2,WNT5B,ZCCHC24,ZFAND2B,ZKSCAN1,ZNF32	
Cancer,Organismal Injury And Abnormalities	Carcinoma	Carcinoma	2.49E-28	-1.432	AASDH,ABCB1,ABCB4,ABCG2,ABCG5,ABCG8,ABTB2,ACAD8,ACAT2,ACLY,ACOX2,ACSL3,ACSL4,ACSL5,ACSM1,ACSM5,ACSS2,ACTR6,ADH4,AFG3L2,AGPAT2,AHCY,AHNAK,AHSA1,AKAP11,AKAP9,AKR1D1,ALAS1,ALDH1L1,ALG13,AMDHD1,ANKRD12,ANP32A,ANXA7,ARF3,ARGLU1,ARHGAP18,ARHGAP21,ARHGAP6,ARIH2,ARL15,ARL4A,ARL6IP4,ARSG,ASAP2,ASB13,ATG3,ATG4D,ATP1A1,ATP1B1,ATP6V1D,ATXN2L,AUH,BAAT,BAP1,BDH1,BHMT,BHMT2,BID,BIK,BLMH,BRD8,BUD23,C11orf54,C9orf152,C9orf16,CA3,CAMKK2,CAPN2,CARS1,CBLB,CCBE1,CCDC66,CCT3,CCT4,CCT5,CCT6A,CCT7,CD2AP,CD47,	582

CD9,CD99L2,CDA,C
DC14B,CDH1,CDH2,
CDO1,CEBPD,CENP
V,CES1,CGN,CHCH
D3,CHCHD4,CHD6,C
HPT1,CHRAC1,CIB3,
CITED2,CLDND1,CL
K2,CLMN,CLOCK,CM
TM6,CMTM8,CNN3,C
NOT4,CNOT6,CNP,C
OL27A1,COL5A3,CO
RO1B,CPEB4,CPT1A
,CRIP2,CRLS1,CROT
,CS,CSRNP1,CTDSP
1,CTPS2,CTSH,CUL4
B,CXADR,CYB5B,CY
LD,CYP2U1,CYP39A
1,CYP4F2,CYP7A1,C
YTH1,DAZAP1,DCTN
1,DDC,DDX60,DEK,D
GKZ,DHX9,DIAPH2,D
ICER1,DIO1,DIPK2A,
DIXDC1,DMD,DMTF1
,DNAJB1,DNAJC5,D
OCK4,DPP4,DPP9,D
SP,DSTN,DTYMK,DU
SP19,DVL1,E2F5,EC
HDC1,EEPD1,EFHD2
,EGR1,EIF1AX,EIF4E
BP1,ELF1,ENDOG,E
NPP1,ERBB3,ESRRA
,EXOC6,EXOSC2,EX
TL2,F11,F2R,F3,FAD
S2,FAM102A,FAM11
8B,FAM160B1,FAM1
72A,FAM76B,FAM89
A,FBXO31,FBXO9,FE
CH,FGA,FGB,FGFR2
,FGFRL1,FGG,FH,FK
BP4,FLCN,FMO1,FM
O5,FOXK1,FOXO3,F
PGS,FTSJ3,G6PD,G
AS2L3,GCAT,GCLC,
GCNT2,GDA,GNA12,
GNG12,GOLGB1,GP
D2,GPN1,GRHPR,GS
DMD,GSK3A,GSS,G
STM2,GTF2A2,GTF2
F1,GTF3C2,GYG1,H
ECA,HECTD3,HERC
4,HLF,HMG20B,HMG
CR,HNRNPC,HNRNP
D,HOMER2,HOOK1,
HRAS,HSD17B2,HSD
3B7,HSPB1,ICK,ID2,I
D4,IGFBP2,IL18,ING
4,IRF6,IRS1,ITPA,ITP
K1,JAK2,JMJD1C,KA
T2B,KCMF1,KDM6A,
KEAP1,KHK,KIAA010
0,KITLG,KLF12,KLF9,
KLHDC2,KLHL21,KL

HL7,KPNB1,KTN1,KY
NU,L2HGDH,LARS2,
LGALS4,LGR5,LIN7A
,LIN7C,LITAF,LMNA,
LPIN1,LRIG1,LRP12,
LRRC28,LRRC3,LSM
14B,LSS,LTBR,MAC
ROH2A1,MAGED1,M
AGI3,MAP1LC3B,MA
P2K3,MAP3K7,MBNL
2,MCOLN1,MEF2A,M
IA2,MKNK2,MLLT10,
MLX,MMD,MOCOS,M
ON2,MPDZ,MPP1,M
PRIP,MRNIP,MRPL1
2,MRPL20,MRPL24,
MRPL44,MRPS22,M
RPS25,MRPS27,MR
PS9,MT1F,MYO1B,M
YO5B,MYO6,N4BP2,
N4BP2L1,NAGK,NCA
LD,NCOA1,NDUFA12
,NDUFA9,NDUFB9,N
EK7,NET1,NFKBIA,N
GEF,NHLRC2,NINJ1,
NIPBL,NME1,NOSIP,
NPC1,NR1H4,NR2F6
,NR3C2,NR5A2,NRIP
1,NT5C,NUDT1,NUD
T19,NUDT7,NUP62,N
UP88,NUS1,OAZ1,O
SBPL1A,OTUD6B,OX
R1,PALMD,PAN2,PA
N3,PAQR9,PCDH1,P
CGF5,PDCD2,PDE2A
,PDE9A,PDGFC,PDG
FRB,PDLIM1,PDP2,P
DPK1,PDRG1,PER1,
PER3,PES1,PEX26,P
EX6,PFDN2,PFKFB1,
PGAP1,PHF20L1,PH
KA2,PIK3AP1,PIK3C
A,PKHD1,PLD1,PLX
NA2,PMPCA,PNKD,P
NRC1,POR,PPIF,PP
M1A,PPOX,PPP1R12
A,PPP1R14B,PPP1R
1B,PPP1R3B,PPP1R
9A,PPP2R1A,PRDX6,
PRPF19,PRPF39,PR
PF6,PRPS1,PRPS2,P
SEN2,PSMA6,PSMD
1,PSMD11,PSMD2,P
SMD8,PSME2,PSMG
1,PSPC1,PSPH,PTB
P1,PTBP2,PXMP4,Q
DPR,RAB11FIP2,RA
B1B,RABEPK,RABG
AP1L,RAI14,RALGPS
2,RAN,RASA3,RASS
F3,RB1CC1,RBBP5,
RBBP7,RBKS,RCAN

1,RCL1,REPIN1,RGS
2,RHBDD1,RHOD,RI
CTOR,RNF14,RNF14
4A,RNF167,ROCK1,
RPA1,RPIA,RUFY3,S
AA4,SAR1B,SARS1,
SBF2,SCAPER,SCA
RB2,SCYL1,SDC4,S
DR42E1,SEC63,SECI
SBP2L,SENP6,SERP
INA6,SERPINE2,SER
PINH1,SESN3,SETD
B2,SFRP5,SGK2,SG
TB,SH3D19,SHMT1,
SHPRH,SIRT5,SLC1
0A1,SLC16A5,SLC1A
4,SLC20A1,SLC20A2
,SLC25A32,SLC27A2
,SLC2A2,SLC2A5,SL
C2A9,SLC35A3,SLC3
5B1,SLC35B3,SLC38
A2,SLC39A10,SLC39
A8,SLC6A12,SLC9A3
R1,SLK,SMAD7,SMA
P1,SNAPC2,SNRNP2
00,SNTB1,SNX10,SN
X5,SNX9,SOAT2,SO
D2,SPARC,SPG21,S
PON2,SPOP,SPTBN
1,SPTBN2,ST3GAL4,
STARD7,STIP1,STK2
4,STK40,STOM,STT3
B,STX18,STX8,STXB
P3,SULF2,SUMF1,SY
AP1,SYNE1,SYT1,TB
C1D16,TBC1D20,TB
C1D24,TBRG4,TCIM,
TCP1,TEP1,TEX2,TH
AP4,THRSP,TIFA,TI
GD2,TIMM10,TIMM4
4,TK1,TLCD4,TLE1,T
MBIM1,TMEM126A,T
MEM219,TMEM41A,T
MEM41B,TMEM51,T
MEM62,TMEM97,TN
FAIP2,TNFAIP8L1,TN
RC6A,TOP1MT,TPI1,
TPR,TPRG1L,TPRKB
,TRIB3,TRMT1,TSN,T
SPAN33,TSTA3,TTC
1,TTC19,TUBB,TUBB
6,TUBGCP2,TXN2,T
XNL1,UBAC1,UBE2F,
UBE2L3,UBR1,UBTF,
UBXN1,UBXN6,UCK1
,UGP2,UGT2B10,UL
K1,UNG,UPP2,UROC
1,UROD,USP47,UTP
14A,UTRN,UXS1,VP
S13A,WDR18,WDR2
6,WDR81,WIPI2,WNT
5B,YPEL3,YWHAH,Z

					CCHC24,ZFAND2B,ZKSCAN1,ZNF23,ZNF32,ZNF326,ZNF654	
Cancer,Organismal Injury And Abnormalities,Reproductive System Disease	Male Genital Neoplasm	Male Genital Neoplasm	1.13E-06	-1.432	ABCB1,ABCB4,ABCG2,ACAD8,ACLY,ACOX2,ACSL3,ACSL4,ACSM1,ACSS2,AGPAT2,AHNAK,AKAP11,ALDH1L1,ALG13,ANKRD12,ARHGAP18,ARHGAP6,ARIH2,ASAP2,ATP1A1,ATP6V1D,ATXN2L,BAAT,BAP1,BDH1,BLMH,BRD8,CA3,CBLB,CD2AP,CDH1,CDH2,CDO1,CES1,CGN,CHD6,CLDND1,CNOT4,CNOT6,CNP,COL5A3,CPT1A,CSRNP1,CYP7A1,CYTH1,DCTN1,DDX60,DGKZ,DICER1,DMD,DOCK4,DSP,EFHD2,EGR1,EIF1AX,EIF4EBP1,ERBB3,F11,FAM160B1,FAM89A,FGA,FGFR2,FGFRL1,FH,FKBP4,FLCN,FOXK1,FOXO3,GDA,GNA12,GOLGB1,GPD2,GSDMD,GTF2A2,HLF,HMGCR,HRAS,HSD3B7,HSPB1,ID2,IGFBP2,ING4,IRS1,JAK2,JMJD1C,KDM6A,KIAA0100,KITLG,KTN1,LARS2,LGALS4,LGR5,LIN7C,LITAF,LRIG1,LRP12,LRRC28,LSS,LTBR,MACROH2A1,MAGI3,MBNL2,MIA2,MKNK2,MLLT10,MMD,MOCOS,MON2,MPDZ,MPRIP,MRPS25,MYO5B,MYO6,N4BP2,NCOA1,NFKBIA,NIPBL,NME1,NRIP1,NUDT7,NUP88,OAZ1,OTUD6B,PAN3,PCDH1,PDGFRB,PDP2,PDPK1,PER1,PEX6,PIK3CA,PKHD1,PLD1,PLXNA2,POR,PPIF,PPP1R1B,PPP1R3B,PPP1R9A,PPP2R1A,PRPF6,PSEN2,PSMD1,PSMD2,PXMP4,RAB11FIP2,RALGPS2,RCAN1,RCL1,REPIN1,RHOD,RNF144A,ROCK1,SBF2,SCAPER,SCARB2,SCYL1,SDC4,SDR42E	203

					1,SECISBP2L,SERPINA6,SERPINE2,SESN3,SIRT5,SLC16A5,SLC1A4,SLC25A32,SLC39A10,SLC6A12,SLK,SMAP1,SNRNP200,SPARC,SPOP,SPTBN1,SPTBN2,ST3GAL4,STOM,STX8,STXBP3,SULF2,SYNE1,TBC1D16,TBC1D24,TIFA,TK1,TNFAIP2,TNRC6A,TOP1MT,TPR,TRMT1,TSPAN33,UBAC1,UBE2F,UBR1,UROC1,USP47,UTRN,VPS13A,YPEL3,ZKSCAN1,ZNF654	
Cancer,Organismal Injury And Abnormalities	Breast Or Colorectal Cancer	Breast Or Colorectal Cancer	4.25E-06	-1.434	AASDH,ABCB1,ABCG2,ABCG5,ACLY,ACOX2,ACSL5,ACSS2,ACTR6,AFG3L2,AGPAT2,AHCY,AKAP9,ALAS1,ALDH1L1,ALG13,ANKRD12,ANXA7,ARHGAP21,ARL6IP4,ASAP2,ASB13,ATG3,ATG4D,ATP1A1,ATXN2L,BAAT,BAP1,C11orf54,C9orf152,CAPN2,CARS1,CCDC66,CCT3,CCT4,CCT5,CCT7,CD2AP,CD9,CD99L2,CDH1,CDH2,CDO1,CEBPD,CES1,CGN,CHCHD3,CHD6,CHPT1,CHRAC1,CLDND1,CLK2,CLMN,CLOCK,CMTM6,CNOT4,CNP,COL27A1,COL5A3,CPEB4,CPT1A,CS,CTDSP1,CTPS2,CUL4B,CYLD,CYP2U1,CYP4F2,DAZAP1,DCTN1,DDX60,DGKZ,DHX9,DICER1,DIXDC1,DMD,DMTF1,DOCK4,DPP4,DSP,DSTN,E2F5,EGR1,EIF1AX,EIF4EBP1,ELF1,ELOVL6,ERBB3,ESRRA,EXOC6,EXOSC2,F2R,F3,FADS2,FAM160B1,FECH,FGA,FGB,FGFR2,FLCN,FMO1,FMO5,FOXO3,FPGS,FTSJ3,G6PD,GAS2L3,GNA12,GNG12,GSS,GSTM2,GTF2F1,HECA,HECTD3,HMGCR,HNRNPC,HRAS,HSD17B2,HSPB1,ICK,ID2,IGFB	302

P2,IL18,ING4,IRF6,IR
S1,JAK2,JMJD1C,KA
T2B,KDM6A,KEAP1,
KIAA0100,KITLG,KLF
12,KLHL7,KPNB1,KT
N1,LGALS4,LGR5,LI
N7A,LMNA,LRIG1,LR
P12,LRRC3,LTBR,M
ACROH2A1,MAGED1
,MAP1LC3B,MAP2K3
,MAP3K7,MEF2A,MIA
2,MKNK2,MLLT10,ML
X,MON2,MPDZ,MPRI
P,MRNIP,MRPL24,M
RPL34,MRPS9,MT1F
,MYO5B,MYO6,NCO
A1,NDUFB9,NEK7,N
ET1,NGEF,NHLRC2,
NIPBL,NME1,NOSIP,
NPC1,NR3C2,NRIP1,
NT5C,NUDT19,NUDT
7,NUP62,NUS1,OSB
PL1A,PAQR9,PCDH1
,PCGF5,PDE9A,PDG
FRB,PDLIM1,PDP2,P
DPK1,PDRG1,PER1,
PFDN2,PGAP1,PHF2
0L1,PHKA2,PIK3AP1,
PIK3CA,PKHD1,PLD
1,PLXNA2,PNKD,PN
RC1,POR,PPP1R14B
,PPP1R1B,PPP1R9A,
PPP2R1A,PRPF39,P
RPF6,PSMD8,PTBP1
,QDPR,RABGAP1L,R
ASA3,RASSF3,RB1C
C1,RBBP5,RGS2,RIC
TOR,RNF14,ROCK1,
RPA1,RUFY3,SAA4,
SBF2,SCAPER,SCYL
1,SEC63,SECISBP2L
,SENP6,SERPINE2,S
ESN3,SFRP5,SGK2,
SHPRH,SLC1A4,SLC
20A1,SLC20A2,SLC2
5A32,SLC27A2,SLC2
A2,SLC35B3,SLC38A
2,SLC39A10,SLC39A
8,SLC6A12,SLC9A3R
1,SMAD7,SMAP1,SN
RNP200,SNTB1,SNX
9,SOAT2,SOD2,SPA
RC,SPG21,SPOP,SP
TBN1,SPTBN2,STAR
D7,STIP1,STX8,SUL
F2,SYAP1,SYNE1,SY
T1,TBC1D16,TBC1D
24,TBRG4,TCP1,TEP
1,THRSP,TIMM44,TK
1,TMEM126A,TMEM4
1B,TMEM97,TNRC6A
,TOP1MT,TPI1,TPR,T

					RIB3,TTC1,TUBB,TUBB6,UBAC1,UBTF,ULK1,UNG,UROC1,USP47,UTRN,VPS13A,WDR18,WDR26,WDR81,WNT5B,YWHAH,ZCCHC24,ZNF23	
Cancer,Organismal Injury And Abnormalities	Incidence	Incidence Of Tumor	9.38E-14	-1.437	AASDH,ABCB1,ABCB4,ABCG2,ABCG5,ACAD8,ACLY,ACOX2,ACSL3,ACSL4,ACSM1,ACSS2,ACTR6,AFG3L2,AGPAT2,AHNAK,AKAP11,AKAP9,ALAS1,ALDH1L1,ALG13,ANKRD12,ANP32A,ANXA7,ARHGAP21,ARHGAP6,ARIH2,ARL15,ARL6IP4,ASAP2,ASB13,ATG3,ATG4D,ATP1A1,ATXN2L,AUH,BAAT,BAP1,BDH1,BHMT,BID,BIK,BRD8,BUD23,C11orf54,C9orf152,C9orf16,CAPN2,CARS1,CBLB,CCDC66,CCT3,CCT5,CCT6A,CCT7,CD2AP,CD47,CD9,CD99L2,CDA,CDC14B,CDH1,CDH2,CDO1,CEBPD,CES1,CGN,CHCHD3,CHD6,CHPT1,CHRAC1,CLDND1,CLK2,CLMN,CLOCK,CMTM6,CMTM8,CNOT4,CNOT6,CNP,COL27A1,COL5A3,CPEB4,CPT1A,CS,CTDSP1,CTPS2,CTSH,CUL4B,CXADR,CYLD,CYP4F2,CYP7A1,DAZAP1,DCTN1,DDX60,DEK,DGKZ,DHX9,DIAPH2,DICER1,DIO1,DIPK2A,DIXDC1,DMD,DMTF1,DOCK4,DPP4,DPP9,DSP,DSTN,DUSP19,DVL1,E2F5,EGR1,EIF1AX,EIF4EBP1,ELF1,ENPP1,ERBB3,ESRRA,EXOC6,EXTL2,F11,F2R,F3,FAM102A,FAM160B1,FAM172A,FECH,FGA,FGB,FGFR2,FGG,FH,FLCN,FMO1,FMO5,FOXO3,FTSJ3,G6PD,GAS2L3,GNA12,GNG12,GOLGB1,GPD2,GRHPR,GSK3A,GSS,GSTM2,GTF2F1,GTF3C2,GYG1,HEC	411

A,HECTD3,HERC4,H
LF,HMGCR,HNRNPC
,HRAS,HSPB1,ICK,ID
2,ID4,IGFBP2,IL18,IN
G4,IRF6,IRS1,ITPA,J
AK2,JMJD1C,KAT2B,
KCMF1,KDM6A,KEA
P1,KIAA0100,KITLG,
KLF12,KLF9,KLHL7,K
PNB1,KTN1,KYNU,L2
HGDH,LGR5,LIN7A,L
MNA,LRIG1,LRP12,L
RRC28,LRRC3,LSM1
4B,LTBR,MACROH2
A1,MAGED1,MAGI3,
MAP2K3,MAP3K7,M
BNL2,MCOLN1,MEF2
A,MIA2,MKNK2,MLLT
10,MLX,MOCOS,MO
N2,MPDZ,MPP1,MPR
IP,MRNIP,MRPL44,M
RPS22,MRPS27,MR
PS9,MYO1B,MYO5B,
MYO6,N4BP2L1,NCO
A1,NDUFA9,NEK7,N
ET1,NFKBIA,NGEF,N
HLRC2,NINJ1,NIPBL,
NME1,NOSIP,NPC1,
NR1H4,NR2F6,NR3C
2,NR5A2,NRIP1,NUD
T1,NUDT19,NUDT7,
NUP62,NUP88,OAZ1,
OSBPL1A,OXR1,PAL
MD,PAN2,PAN3,PAQ
R9,PCDH1,PCGF5,P
DCD2,PDE2A,PDE9A
,PDGFC,PDGFRB,PD
LIM1,PDP2,PDPK1,P
ER1,PER3,PFDN2,P
GAP1,PHF20L1,PHK
A2,PIK3AP1,PIK3CA,
PKHD1,PLD1,PLXNA
2,PMPCA,PNKD,PNR
C1,POR,PPIF,PPP1R
12A,PPP1R14B,PPP
1R1B,PPP1R9A,PPP
2R1A,PRDX6,PRPF1
9,PRPF39,PRPF6,PS
EN2,PSMA6,PSMD1,
PSMD2,PSMD8,PTB
P1,PTBP2,QDPR,RA
BEPK,RABGAP1L,RA
I14,RALGPS2,RASSF
3,RB1CC1,RBBP5,R
BBP7,RCL1,RGS2,R
HBDD1,RICTOR,RO
CK1,RPA1,RPIA,RUF
Y3,SAA4,SARS1,SBF
2,SCAPER,SCARB2,
SCYL1,SDC4,SEC63,
SECISBP2L,SENP6,
SERPINE2,SERPINH

					1,SESN3,SETDB2,SGTB,SHPRH,SLC1A4,SLC20A1,SLC20A2,SLC2A2,SLC2A5,SLC2A9,SLC30A10,SLC35B3,SLC38A2,SLC39A10,SLC39A8,SLC6A12,SLC9A3R1,SMAD7,SMAP1,SNRNP200,SNTB1,SNX10,SNX9,SOAT2,SOD2,SPARC,SPON2,SPOP,SPTBN1,SPTBN2,STARD7,STIP1,STK24,STK40,STOM,STT3B,STX8,STXBP3,SULF2,SYAP1,SYNE1,SYT1,TBC1D16,TBC1D20,TBC1D24,TBRG4,TCP1,TEP1,TEX2,TIGD2,TIMM44,TK1,TLCD4,TLE1,TMEM126A,TMEM41B,TMEM51,TMEM97,TNFAIP2,TNRC6A,TOP1MT,TPI1,TPR,TPRG1L,TRIB3,TSPAN33,TTC1,TUBB,TUBB6,TXNL1,UBAC1,UBE2F,UBTF,UGP2,UGT2B10,ULK1,UNG,UROC1,UTP14A,UTRN,UXS1,VPS13A,WDR18,WDR26,WDR81,WIPI2,WNT5B,ZCCHC24,ZFAND2B,ZKSCAN1,ZNF32	
Cancer,Organismal Injury And Abnormalities,Reproductive System Disease	Mammary Tumor	Mammary Tumor	7,31E-04	-1,489	ABCB1,ABCG2,ACSS2,AGPAT2,AKAP9,ALDH1L1,ANKRD12,ATP1A1,BAP1,CAPN2,CCT3,CD9,CDH1,CDH2,CEBPD,CES1,CLK2,COL27A1,CPT1A,CYLD,CYP2U1,DAZAP1,DGKZ,DICER1,DIXDC1,DMD,DMTF1,DOCK4,DPP4,DSP,E2F5,EGR1,EIF1AX,EIF4EBP1,ELOVL6,ERBB3,ESRRA,EXOC6,EXOSC2,F2R,FADS2,FGA,FGFR2,FOXO3,FTSJ3,GNA12,GSTM2,HMGCR,HRAS,HSPB1,IGFBP2,IL18,ING4,IRS1,JAK2,JMJD1C,KDM6A,KEAP1,KIAA0100,LGALS4,LMNA,MACROH2A1,MAP1LC3B,MAP2K3,MKNK2,MPDZ,MRPL24,MRPL34,MT1F,MYO5B	130

					,MYO6,NDUFB9,NEK7,NFKBIA,NIPBL,NME1,NPC1,NR3C2,NRIP1,NUS1,PDGFRB,PDPK1,PGAP1,PHF20L1,PHKA2,PIK3CA,PKHD1,PNRC1,POR,PPP1R1B,PPP2R1A,PRPF39,PTBP1,RASA3,RB1CC1,RGS2,RICTOR,RNF14,ROCK1,SBF2,SECISBP2L,SFRP5,SLC20A1,SLC25A32,SLC9A3R1,SNTB1,SOD2,SPARC,SPG21,SPOP,SPTBN2,STARD7,STIP1,SYNE1,TCP1,TEP1,THRSP,TIMM44,TK1,TLE1,TMEM97,TNRC6A,TOP1MT,TPI1,TUBB,TUBB6,USP47,UTRN,WNT5B,YWHAH	
Cancer,Gastrointestinal Disease,Organismal Injury And Abnormalities	Development	Development Of Digestive Organ Tumor	1.16E-05	-1.492	AASDH,ABCB1,ABCB4,ABCG5,ACLY,ACSS2,ACTR6,AFG3L2,AGPAT2,AHNAK,AKAP9,ALDH1L1,ALG13,ANXA7,ARHGAP21,ARL6IP4,ASAP2,ASB13,ATG3,ATG4D,ATXN2L,BAAT,BAP1,BHMT,BRD8,C11orf54,C9orf152,CARS1,CBLB,CCDC66,CCT7,CD2AP,CD99L2,CDH1,CDH2,CDO1,CES1,CGN,CHCHD3,CHD6,CHPT1,CHRAC1,CLDND1,CLK2,CLMN,CLOCK,CMTM6,CNOT4,CNP,COL27A1,COL5A3,CPEB4,CPT1A,CS,CTDSP1,CTPS2,CUL4B,CYLD,CYP4F2,DAZAP1,DCTN1,DDX60,DGKZ,DHX9,DICER1,DMD,DMTF1,DOCK4,DSP,DSTN,EGR1,ELF1,ERBB3,ESRRA,F3,FAM102A,FAM160B1,FGB,FGFR2,FLCN,FMO1,FMO5,G6PD,GAS2L3,GNG12,GPD2,GSS,GTF2F1,HECA,HECTD3,HMGCR,HNRNPC,HRAS,HSPB1,ICK,IGFBP2,IL18,ING4,IRF6,IRS1,JAK2,JMJD1C,KAT2B,KDM6A,KEAP1,KIAA0100,KITLG,KLF12,KLHL7,K	257

					PNB1,KTN1,KYNU,LGR5,LIN7A,LRIG1,LRP12,LRRC3,LTBR,MAGED1,MAP3K7,MBNL2,MEF2A,MIA2,MKNK2,MLLT10,MLX,MON2,MPDZ,MPRIP,MRNIP,MRPS9,MYO5B,MYO6,NCOA1,NEK7,NET1,NGEF,NHLRC2,NIPBL,NOSIP,NR1H4,NR3C2,NRIP1,NUDT1,NUDT19,NUDT7,NUP62,OSBPL1A,PAN2,PAN3,PAQR9,PCDH1,PCGF5,PDE2A,PDE9A,PDGFC,PDGFRB,PDP2,PDPK1,PER1,PER3,PFDN2,PGAP1,PHF20L1,PHKA2,PIK3AP1,PIK3CA,PKHD1,PLXNA2,PMPCA,PNKD,PNRC1,POR,PPP1R12A,PPP1R14B,PPP1R9A,PPP2R1A,PRPF39,PRPF6,PSMD8,PTBP1,QDPR,RABGAP1L,RASSF3,RB1CC1,RBBP5,RICTOR,ROCK1,RPA1,RUFY3,SAA4,SARS1,SBF2,SCAPER,SCYL1,SDC4,SEC63,SECISBP2L,SENP6,SERPINE2,SERPINH1,SESN3,SETDB2,SGTB,SHPRH,SLC1A4,SLC20A1,SLC20A2,SLC2A2,SLC38A2,SLC39A10,SLC39A8,SLC6A12,SMAD7,SMAP1,SNRNP200,SNTB1,SNX9,SOAT2,SPOP,SPTBN1,SPTBN2,STIP1,STX8,SULF2,SYAP1,SYNE1,SYT1,TBC1D16,TBC1D24,TBRG4,TCP1,TEP1,TIMM44,TK1,TMEM126A,TMEM41B,TMEM97,TNRC6A,TOP1MT,TPR,TTC1,TUBB,UBAC1,UBTF,ULK1,UNG,UROC1,UTRN,VPS13A,WDR18,WDR26,WDR81,WIPI2,WNT5B,ZCCHC24,ZNF32	
Cancer,Organismal Injury And Abnormalities	Neoplasia	Lymphatic System Tumor	9.85E-05	-1.493	ABCB1,ABCG2,AHNAK,AKAP11,ANXA7,ASAP2,ATP1B1,BAP1,BID,CBLB,CCT3,CCT7,CD47,CDH1,CDO1,	114

					CEBPD,CHD6,CITED2,CNP,COL27A1,CTSH,CUL4B,DDC,DEK,DGKZ,DICER1,DIO1,DMD,DMTF1,DPP4,DSP,EGR1,EIF4EBP1,ENPP1,ERBB3,EXTL2,F2R,F3,FAM102A,FBXO31,FBXO9,FGFR2,FOXO3,FPGS,GNA12,GPD2,GRHPR,HECA,HMGCR,HRAS,HSPB1,ICK,ID2,IL18,JAK2,JMJD1C,KDM6A,KIAA0100,KITLG,KPNB1,LPIN1,LRIG1,LTBR,MAGED1,MAP2K3,MEF2A,MIA2,MKNK2,MLLT10,MPDZ,MRPL20,MYO5B,NFKBIA,NINJ1,NIPBL,NME1,PALMD,PDE9A,PDGFC,PDGFRB,PDPK1,PER1,PIK3CA,PLXNA2,PNKD,PNRC1,POLE4,POR,PRPF19,PRPS1,PSMD1,PSMD2,RAN,RICTOR,RPA1,SARS1,SERPINH1,SHMT1,SLC25A32,SMAD7,SOD2,SPARC,SPTBN1,STIP1,STK24,SYT1,TBC1D16,TPR,TUBB6,TUT4,UBE2F,UBR1,UNG,VPS13A	
Developmental Disorder	Growth Failure	Growth Failure	3.12E-04	-1.498	AQP11,BAP1,CD2AP,CDH2,CUL4B,CYP7A1,DMTF1,DNAJC5,EGR1,F11,F2R,F3,FGFR2,FGFRL1,FKBP4,GCLC,GPD2,HNRNPD,HSD17B2,HSD3B7,ID2,IRS1,ITPA,KAT2B,KDM6A,LMNA,MACROH2A1,MAP3K7,MCOLN1,NEK7,NFKBIA,NINJ1,PDCD2,PGAP1,POR,PSEN2,SCYL1,SFRP5,SLC20A1,SOD2,SULF2,SYNE1	42
Cancer,Organismal Injury And Abnormalities	Extraadrenal Retroperitoneal Tumor	Extraadrenal Retroperitoneal Tumor	3.97E-06	-1.526	AASDH,ABCB1,ABCB4,ABCG2,ABTB2,ACAT2,ACLY,ACOX2,ACSM5,ADH4,AHNAK,AKAP11,AKAP9,ALAS1,ALG13,ANKRD12,ANP32A,ARHGAP21,ATXN2L,BAP1,BRD8,C9orf152,CAMKK2,CAPN2,CBLB,CCDC	188

					66,CCT3,CD9,CDC14 B,CDH1,CDH2,CHD6 ,COL5A3,CUL4B,CYL D,CYP4F2,DCTN1,D HX9,DIAPH2,DICER1 ,DIO1,DMD,DOCK4,D PP4,DUSP19,E2F5,E CHDC3,EGR1,EIF1A X,ERBB3,ESRRA,EX OC6,EXTL2,F11,F3,F GA,FGFR2,FH,FLCN, FMO1,FOXO3,FTSJ3 ,G6PD,GABARAP,GA S2L3,GCLC,GOLGB1 ,GRHPR,GTF3C2,HE RC4,HMGCR,HNRNP C,HRAS,ID2,IL18,IRF 6,IRS1,JAK2,KDM6A, KEAP1,KIAA0100,KL F12,KLF9,KLHL7,LG R5,LIN7A,LMNA,LRI G1,LSM14B,LSS,MA GI3,MAP2K3,MIA2,M KNK2,MLLT10,MOC OS,MPDZ,MPP1,MR PL24,MRPS27,MYO1 B,MYO5B,N4BP2L1, NAGK,NCOA1,NFKBI A,NGEF,NINJ1,NIPB L,NME1,NPC1,NR5A 2,NRIP1,NUP62,PAN 2,PAQR9,PCDH1,PD E2A,PDGFRB,PHKA2 ,PIK3CA,PKHD1,PLX NA2,PNKD,POR,PPP 1R1B,PPP1R9A,PPP 2R1A,PRPF6,PRPS1, PSMD1,PSMD11,PS MD2,PSMF1,PXMP4, RAB11FIP2,RAI14,R B1CC1,RBBP5,RCL1, RHOD,RPA1,SBF2,S CAPER,SEC63,SENP 6,SERPINE2,SERPIN H1,SETDB2,SHMT1, SHPRH,SLC1A4,SLC 20A2,SLC2A5,SLC2A 9,SLC35B1,SLC39A8 ,SLC9A3R1,SMAD7, SNRNP200,SNX10,S OD2,SPARC,SPTBN 1,ST3GAL4,STARD7, STXBP3,SYNE1,TEP 1,TEX2,TMEM126A,T MEM51,TNRC6A,TO P1MT,TRIB3,TUBB,T UBB6,TUBGCP2,TXN L1,UBE2F,UGP2,UR OD,UTP14A,UTRN,V PS13A,WNT5B,ZFAN D2B,ZNF654	
Cancer,Gastrointesti	Hepatobiliary	Hepatobiliary	1.24E-07	-1.542	ABCB4,ABCG8,ACLY	252

nal Disease,Hepatic System Disease,Organismal Injury And Abnormalities	System Cancer	System Cancer	,ACOX2,ACSS2,ADH4,AHNAK,AKAP11,AKAP9,AKR1D1,AMDHD1,ANKRD12,ANXA7,ARGLU1,ARHGAP21,ARHGAP6,ASAP2,ASB13,ATP1A1,ATP1B1,ATXN2L,BAAT,BAP1,BDH1,BHMT,BHMT2,BUD23,CARS1,CBLB,CCT6A,CD47,CDH1,CDH2,CES1,CHD6,CIB3,CLK2,CLMN,CMTM6,CNOT4,COL27A1,COL5A3,CPEB4,CRIP2,CROT,CS,CTPS2,CUL4B,CXADR,CYP39A1,CYP7A1,CYTH1,DDC,DEK,DIAPH2,DICER1,DIPK2A,DMD,DOCK4,DPP4,DPP9,E2F5,EIF1AX,EIF4EBP1,ENPP1,ERBB3,ESRRA,EXTL2,F3,FAM160B1,FBXO9,FGA,FGB,FGFR2,FGFRL1,FGG,FLCN,FOXK1,FPGS,G6PD,GAS2L3,GCLC,GNA12,GOLGB1,GPD2,GRHPR,GSDMD,GTF3C2,HECA,HLF,HMGCR,HRAS,ID2,ID4,IGFBP2,IL18,ING4,IRS1,JMJD1C,KDM6A,KEAP1,KIAA0100,KLHDC2,KPNB1,KTN1,KYNU,LARS2,LGR5,LIN7A,LMNA,LTBR,MACROH2A1,MAGI3,MAP1LC3B,MAP2K3,MAP3K7,MEF2A,MON2,MPDZ,MPRIP,MRNIP,MRPL24,MRPS22,MYO6,NCALD,NCOA1,NET1,NGEF,NIPBL,NME1,NPC1,NR1H4,NR2F6,NR3C2,NR5A2,NRIP1,NT5C,OSBPL1A,OXR1,PAN2,PAN3,PAQR9,PCDH1,PDE2A,PDGFC,PDGFRB,PDLIM1,PER1,PEX26,PGAP1,PHF20L1,PIK3AP1,PIK3CA,PKHD1,PLD1,PLXNA2,PMPCA,PNRC1,POR,PPM1A,PPOX,PPP1R1B,PPP1R9A,PPP2R1A,PRPF6,PSMD1,PSMD11,PSMD2,PSPH,PTBP2,RABGAP1L,RA

					LGPS2,RAN,RASA3,RB1CC1,RBBP5,RCL1,RICTOR,RNF167,ROCK1,RUFY3,SAA4,SARS1,SCAPER,SDC4,SECISBP2L,SENP6,SERPINA6,SESN3,SFRP5,SGK2,SGTB,SIRT5,SLC10A1,SLC1A4,SLC20A1,SLC20A2,SLC2A2,SLC2A5,SLC35B3,SLC38A2,SLC39A10,SLC39A8,SLC6A12,SLK,SNRNP200,SNTB1,SNX9,SOAT2,SOD2,SPARC,SPTBN1,SPTBN2,STK24,STK40,SULF2,SUMF1,SYNE1,TBC1D16,TCIM,TEP1,TEX2,THAP4,TIGD2,TIMM44,TLE1,TMEM219,TMEM41A,TMEM62,TNFAIP8L1,TNRC6A,TOP1MT,TPI1,TPR,TSN,TSPAN33,TTC1,TUBB,TUBGCP2,TXNL1,UBAC1,UBXN1,UBXN6,UCK1,UGP2,UGT2B10,UPP2,UROC1,USP47,UTRN,YWHAH,ZCCHC24	
Cancer,Gastrointestinal Disease,Hepatic System Disease,Organismal Injury And Abnormalities	Cancer	Liver Cancer	1.68E-07	-1.542	ABCB4,ABCG8,ACLY,ACOX2,ACSS2,ADH4,AHNAK,AKAP9,AKR1D1,AMDHD1,ANKRD12,ANXA7,ARGLU1,ARHGAP21,ARHGAP6,ASAP2,ASB13,ATP1A1,ATP1B1,ATXN2L,BAP1,BDH1,BHMT,BHMT2,BUD23,CARS1,CBLB,CCT6A,CD47,CDH1,CDH2,CES1,CHD6,CIB3,CLK2,CLMN,CMTM6,CNOT4,COL27A1,CPEB4,CRIP2,CROT,CXADR,CYP39A1,CYP7A1,CYTH1,DDC,DIAPH2,DICER1,DIPK2A,DMD,DOCK4,DPP4,DPP9,E2F5,EIF1AX,EIF4EBP1,ENPP1,ERBB3,ESRRA,EXTL2,F3,FAM160B1,FBXO9,FGA,FGB,FGFR2,FGG,FLCN,FOXK1,FPGS,G6PD,GAS2L3,GCLC,GNA12,GOLGB1,GPD2,GRHPR,GSDMD,GTF3C2,HECA,HLF,	240

					HMGCR,HRAS,ID2,ID4,IGFBP2,IL18,ING4,IRS1,JMJD1C,KDM6A,KEAP1,KIAA0100,KLHDC2,KPNB1,KTN1,KYNU,LARS2,LGR5,LMNA,LTBR,MACROH2A1,MAGI3,MAP1LC3B,MAP2K3,MAP3K7,MEF2A,MON2,MPDZ,MPRIP,MRNIP,MRPL24,MRPS22,MYO6,NCALD,NCOA1,NET1,NGEF,NIPBL,NME1,NPC1,NR1H4,NR2F6,NR3C2,NR5A2,NRIP1,NT5C,OSBPL1A,OXR1,PAN2,PAN3,PAQR9,PCDH1,PDE2A,PDGFC,PDGFRB,PDLIM1,PER1,PEX26,PGAP1,PHF20L1,PIK3AP1,PIK3CA,PKHD1,PLD1,PLXNA2,PMPCA,PNRC1,POR,PPM1A,PPOX,PPP1R1B,PPP1R9A,PPP2R1A,PRPF6,PSMD1,PSMD11,PSMD2,PSPH,PTBP2,RABGAP1L,RALGPS2,RAN,RASA3,RB1CC1,RBBP5,RCL1,RICTOR,RNF167,ROCK1,RUFY3,SAA4,SARS1,SCAPER,SDC4,SECISBP2L,SENP6,SESN3,SFRP5,SGK2,SGTB,SIRT5,SLC10A1,SLC1A4,SLC20A2,SLC2A2,SLC2A5,SLC35B3,SLC38A2,SLC39A10,SLC39A8,SLC6A12,SLK,SNRNP200,SNX9,SOAT2,SOD2,SPARC,SPTBN1,SPTBN2,STK24,STK40,SULF2,SUMF1,SYNE1,TBC1D16,TCIM,TEP1,TEX2,THAP4,TIGD2,TIMM44,TLE1,TMEM219,TMEM41A,TMEM62,TNFAIP8L1,TNRC6A,TOP1MT,TPI1,TPR,TSN,TSPAN33,TTC1,TUBB,TUBGCP2,TXNL1,UBAC1,UBXN1,UBXN6,UCK1,UGP2,UGT2B10,UPP2,UROC1,USP47,UTRN,YWHAH,ZCCHC24	
Cell-To-Cell	Adhesion	Adhesion Of	4.44E-04	-1.562	ARHGAP21,CD47,CD	25

Signaling And Interaction		Tumor Cell Lines			9,CDH1,CDH2,CYTH1,DSP,EGR1,ERBB3,F2R,FGA,FGFR2,HRAS,IL18,IRS1,LGALS4,LTBR,MYO6,NET1,NFKBIA,NME1,PLD1,RCAN1,TCIM,UTRN	
Cancer,Organismal Injury And Abnormalities	Non-Melanoma Solid Tumor	Non-Melanoma Solid Tumor	5.36E-28	-1.591	AASDH,ABCB1,ABCB4,ABCG2,ABCG5,ABCG8,ABTB2,ACAD8,ACAT2,ACLY,ACOX2,ACSL3,ACSL4,ACSL5,ACSM1,ACSM5,ACSS2,ACTR6,ADH4,AFG3L2,AGPAT2,AHCY,AHNAK,AHSA1,AKAP11,AKAP9,AKR1D1,ALAS1,ALDH1L1,ALG13,AMDHD1,ANKRD12,ANP32A,ANXA7,ARF3,ARGLU1,ARHGAP18,ARHGAP21,ARHGAP6,ARIH2,ARL15,ARL4A,ARL6IP4,ARSG,ASAP2,ASB13,ATG3,ATG4D,ATP1A1,ATP1B1,ATP6V1D,ATXN2L,AUH,BAAT,BAP1,BDH1,BHMT,BHMT2,BID,BIK,BLMH,BRD8,BUD23,C11orf54,C9orf152,C9orf16,CA3,CAMKK2,CAPN2,CARS1,CBLB,CCBE1,CCDC66,CCT3,CCT4,CCT5,CCT6A,CCT7,CD2AP,CD47,CD9,CD99L2,CDA,CDC14B,CDH1,CDH2,CDO1,CEBPD,CENPV,CES1,CGN,CHCHD3,CHCHD4,CHD6,CHPT1,CHRAC1,CIB3,CITED2,CLDND1,CLK2,CLMN,CLOCK,CMTM6,CMTM8,CNN3,CNOT4,CNOT6,CNP,COL27A1,COL5A3,CORO1B,CPEB4,CPT1A,CRIP2,CRLS1,CROT,CS,CSRNP1,CTDSP1,CTPS2,CTSH,CUL4B,CXADR,CYB5B,CYLD,CYP2U1,CYP39A1,CYP4F2,CYP7A1,CYTH1,DAZAP1,DCTN1,DDC,DDX60,DEK,DGKZ,DHX9,DIAPH2,DICER1,DIO1,DIPK2A,DIXDC1,DMD,DMTF1,DNAJB1,DNAJC5,DOCK4,DPP4,DPP9,D	585

SP,DSTN,DTYMK,DU
SP19,DVL1,E2F5,EC
HDC1,EEPD1,EFHD2
,EGR1,EIF1AX,EIF4E
BP1,ELF1,ENDOG,E
NPP1,ERBB3,ESRRA
,EXOC6,EXOSC2,EX
TL2,F11,F2R,F3,FAD
S2,FAM102A,FAM11
8B,FAM160B1,FAM1
72A,FAM76B,FAM89
A,FBXO31,FBXO9,FE
CH,FGA,FGB,FGFR2
,FGFRL1,FGG,FH,FK
BP4,FLCN,FMO1,FM
O5,FOXK1,FOXO3,F
PGS,FTSJ3,G6PD,G
AS2L3,GCAT,GCLC,
GCNT2,GDA,GNA12,
GNG12,GOLGB1,GP
D2,GPN1,GRHPR,GS
DMD,GSK3A,GSS,G
STM2,GTF2A2,GTF2
F1,GTF3C2,GYG1,H
ECA,HECTD3,HERC
4,HLF,HMG20B,HMG
CR,HNRNPC,HNRNP
D,HOMER2,HOOK1,
HRAS,HSD17B2,HSD
3B7,HSPB1,ICK,ID2,I
D4,IGFBP2,IL18,ING
4,IRF6,IRS1,ITPA,ITP
K1,JAK2,JMJD1C,KA
T2B,KCMF1,KDM6A,
KEAP1,KHK,KIAA010
0,KITLG,KLF12,KLF9,
KLHDC2,KLHL21,KL
HL7,KPNB1,KTN1,KY
NU,L2HGDH,LARS2,
LGALS4,LGR5,LIN7A
,LIN7C,LITAF,LMNA,
LPIN1,LRIG1,LRP12,
LRRC28,LRRC3,LSM
14B,LSS,LTBR,MAC
ROH2A1,MAGED1,M
AGI3,MAP1LC3B,MA
P2K3,MAP3K7,MBNL
2,MCOLN1,MEF2A,M
IA2,MKNK2,MLLT10,
MLX,MMD,MOCOS,M
ON2,MPDZ,MPP1,M
PRIP,MRNIP,MRPL1
2,MRPL20,MRPL24,
MRPL44,MRPS22,M
RPS25,MRPS27,MR
PS9,MT1F,MYO1B,M
YO5B,MYO6,N4BP2,
N4BP2L1,NAGK,NCA
LD,NCOA1,NDUFA12
,NDUFA9,NDUFB9,N
EK7,NET1,NFKBIA,N
GEF,NHLRC2,NINJ1,

NIPBL,NME1,NOSIP,
NPC1,NR1H4,NR2F6
,NR3C2,NR5A2,NRIP
1,NT5C,NUDT1,NUD
T19,NUDT7,NUP62,N
UP88,NUS1,OAZ1,O
SBPL1A,OTUD6B,OX
R1,PALMD,PAN2,PA
N3,PAQR9,PCDH1,P
CGF5,PDCD2,PDE2A
,PDE9A,PDGFC,PDG
FRB,PDLIM1,PDP2,P
DPK1,PDRG1,PER1,
PER3,PES1,PEX26,P
EX6,PFDN2,PFKFB1,
PGAP1,PHF20L1,PH
KA2,PIK3AP1,PIK3C
A,PKHD1,PLD1,PLX
NA2,PMPCA,PNKD,P
NRC1,POLE4,POR,P
PIF,PPM1A,PPOX,PP
P1R12A,PPP1R14B,
PPP1R1B,PPP1R3B,
PPP1R9A,PPP2R1A,
PRDX6,PRPF19,PRP
F39,PRPF6,PRPS1,P
RPS2,PSEN2,PSMA6
,PSMD1,PSMD11,PS
MD2,PSMD8,PSME2,
PSMG1,PSPC1,PSP
H,PTBP1,PTBP2,PX
MP4,QDPR,RAB11FI
P2,RAB1B,RABEPK,
RABGAP1L,RAI14,R
ALGPS2,RAN,RASA3
,RASSF3,RB1CC1,R
BBP5,RBBP7,RBKS,
RCAN1,RCL1,REPIN
1,RGS2,RHBDD1,RH
OD,RICTOR,RNF14,
RNF144A,RNF167,R
OCK1,RPA1,RPIA,R
UFY3,SAA4,SAR1B,S
ARS1,SBF2,SCAPER
,SCARB2,SCYL1,SD
C4,SDR42E1,SEC63,
SECISBP2L,SENP6,
SERPINA6,SERPINE
2,SERPINH1,SESN3,
SETDB2,SFRP5,SGK
2,SGTB,SH3D19,SH
MT1,SHPRH,SIRT5,S
LC10A1,SLC16A5,SL
C1A4,SLC20A1,SLC2
0A2,SLC25A32,SLC2
7A2,SLC2A2,SLC2A5
,SLC2A9,SLC30A10,
SLC35A3,SLC35B1,S
LC35B3,SLC38A2,SL
C39A10,SLC39A8,SL
C6A12,SLC9A3R1,SL
K,SMAD7,SMAP1,SN

					APC2,SNRNP200,SNTB1,SNX10,SNX5,SNX9,SOAT2,SOD2,SPARC,SPG21,SPON2,SPOP,SPTBN1,SPTBN2,ST3GAL4,STARD7,STIP1,STK24,STK40,STOM,STT3B,STX18,STX8,STXBP3,SULF2,SUMF1,SYAP1,SYNE1,SYT1,TBC1D16,TBC1D20,TBC1D24,TBRG4,TCIM,TCP1,TEP1,TEX2,THAP4,THRSP,TIFA,TIGD2,TIMM10,TIMM44,TK1,TLCD4,TLE1,TMBIM1,TMEM126A,TMEM219,TMEM41A,TMEM41B,TMEM51,TMEM62,TMEM97,TNFAIP2,TNFAIP8L1,TNRC6A,TOP1MT,TPI1,TPR,TPRG1L,TPRKB,TRIB3,TRMT1,TSN,TSPAN33,TSTA3,TTC1,TTC19,TUBB,TUBB6,TUBGCP2,TUT4,TXN2,TXNL1,UBAC1,UBE2F,UBE2L3,UBR1,UBTF,UBXN1,UBXN6,UCK1,UGP2,UGT2B10,ULK1,UNG,UPP2,UROC1,UROD,USP47,UTP14A,UTRN,UXS1,VPS13A,WDR18,WDR26,WDR81,WIPI2,WNT5B,YPEL3,YWHAH,ZCCHC24,ZFAND2B,ZKSCAN1,ZNF23,ZNF32,ZNF326,ZNF654	
Cardiovascular System Development And Function,Cell-To-Cell Signaling And Interaction	Binding	Binding Of Vascular Endothelial Cells	3.54E-04	-1.642	ANXA7,CD47,CDH2,F11,F2R,F3,KITLG,LGALS4,LTBR,NFKBIA,RCAN1,RICTOR,SPARC	13
Dermatological Diseases And Conditions,Inflammatory Disease,Inflammatory Response,Organismal Injury And Abnormalities	Dermatitis	Dermatitis	8.53E-04	-1.663	AHCY,AHNAK,AKR1D1,ARIH2,ATP1A1,BLMH,CD47,CDH1,CNN3,DPP4,DSP,FADS2,FKBP4,FOXO3,FPGS,IL18,JAK2,MAP2K3,NAGK,NFKBIA,NR1H4,PER1,POR,PSEN2,PSMD1,PSMD2,SLC25A32,SMAD7,STARD7,TPI1	30
Lipid Metabolism,Small Molecule Biochemistry,Vitami	Metabolism	Metabolism Of Terpenoid	1.58E-05	-1.666	ABCG5,ABCG8,ACAT2,ACLY,ADH4,AKR1D1,BAAT,CES1,CYP39A1,CYP4F2,CYP7	29

n And Mineral Metabolism					A1,ELOVL6,FECH,G6PD,HMGCR,HSD17B2,HSD3B7,KPNB1,LSS,NPC1,NR1H4,NR5A2,NUS1,POR,RAN,SDC4,SERPINA6,SOAT2,YWHAH	
Cancer,Organismal Injury And Abnormalities	Lymphohematopoietic Cancer	Lymphohematopoietic Cancer	1.11E-05	-1.676	ABCB1,ABCB4,ABCG2,ABCG5,ABCG8,ACSL4,AHNAK,AKAP11,ALAS1,ANXA7,ASAP2,ATG4D,ATP1A1,ATP1B1,BAAT,BAP1,BHMT,CAPN2,CBLB,CCT3,CCT7,CD47,CDA,CDH1,CDO1,CEBPD,CHD6,CITED2,CNP,COL27A1,CPT1A,CTPS2,CTSH,CUL4B,DAZAP1,DDC,DDX60,DEK,DGKZ,DHX9,DICER1,DIO1,DIPK2A,DMD,DMTF1,DNAJC5,DPP4,DSP,ECHDC3,EGR1,EIF4EBP1,ENPP1,ERBB3,EXTL2,F2R,F3,FAM102A,FBXO31,FBXO9,FGFR2,FGG,FOXO3,FPGS,GNA12,GPD2,GRHPR,GYG1,HECA,HECTD3,HMG20B,HMGCR,HNRNPD,HOOK1,HRAS,HSPB1,ICK,ID2,ID4,IL18,IRS1,JAK2,JMJD1C,KDM6A,KIAA0100,KITLG,KPNB1,LARS2,LGALS4,LMNA,LPIN1,LRIG1,LRRC3,LTBR,MAGED1,MAP2K3,MEF2A,MIA2,MKNK2,MLLT10,MPDZ,MRPL20,MRPL44,MYO5B,NET1,NFKBIA,NINJ1,NIPBL,NME1,NRIP1,NT5C,NUP88,OSBPL1A,PALMD,PAN3,PAQR9,PDE9A,PDGFC,PDGFRB,PDPK1,PER1,PER3,PES1,PEX6,PIK3AP1,PIK3CA,PKHD1,PLXNA2,PNKD,PNRC1,POLE4,POR,PPM1A,PPP1R14B,PPP1R1B,PPP1R3B,PRPF19,PRPS1,PSEN2,PSMD1,PSMD2,PSME2,RAN,RHOD,RICTOR,RPA1,SARS1,SCARB2,SERPINH1,SGK2,SHMT1,SHPRH,SLC25A32,SM	176

					AD7,SOAT2,SOD2,SPARC,SPTBN1,STIP1,STK24,STK40,SYNE1,SYT1,TBC1D16,TCIM,TEP1,TPI1,TPR,TUBB6,TUT4,UBE2F,UBR1,UBXN1,UGT2B10,UNG,UROC1,VPS13A	
Carbohydrate Metabolism	Uptake	Uptake Of Carbohydrate	7.87E-04	-1.68	ABCB4,COL5A3,CPT1A,CYLD,DPP4,EGR1,ERBB3,ESRRA,FGFR2,G6PD,GSK3A,IRS1,NCOA1,NR1H4,NRIP1,PDPK1,PIK3CA,PLD1,PPM1A,SLC10A1,SLC2A2,SLC2A5,TRIB3	23
Cancer,Gastrointestinal Disease,Organismal Injury And Abnormalities	Colon Tumor	Colon Tumor	2.01E-05	-1.698	AASDH,ABCB1,ABCG5,ACLY,ACTR6,AFG3L2,AGPAT2,AHCY,AKAP9,ALDH1L1,ALG13,ARHGAP21,ARL6IP4,ASAP2,ASB13,ATG3,ATG4D,ATXN2L,BAAT,BAP1,C11orf54,C9orf152,CARS1,CCDC66,CCT4,CCT5,CCT7,CD2AP,CD99L2,CDH1,CDH2,CDO1,CES1,CGN,CHCHD3,CHD6,CHPT1,CHRAC1,CLDND1,CLK2,CLMN,CLOCK,CMTM6,CNOT4,CNP,COL27A1,COL5A3,CPEB4,CPT1A,CS,CTDSP1,CTPS2,CUL4B,CYLD,CYP4F2,DCTN1,DDX60,DHX9,DICER1,DMD,DMTF1,DOCK4,DSTN,EGR1,ELF1,ERBB3,ESRRA,F3,FAM160B1,FGA,FGB,FGFR2,FLCN,FMO1,FMO5,G6PD,GAS2L3,GNA12,GNG12,GSS,HECA,HECTD3,HMGCR,HNRNPC,HSD17B2,HSPB1,ICK,IGFBP2,IL18,IRF6,IRS1,JAK2,JMJD1C,KDM6A,KEAP1,KIAA0100,KITLG,KLF12,KLHL7,KPNB1,KTN1,LGR5,LIN7A,LRIG1,LRP12,LRRC3,MACROH2A1,MAGED1,MAP3K7,MIA2,MKNK2,MLLT10,MLX,MON2,MPRIP,MRNIP,MRPS9,MT1F,MYO5B,MYO6,NCOA1,NEK	230

					7,NET1,NGEF,NHLRC2,NIPBL,NOSIP,NR1H4,NR3C2,NUDT19,NUDT7,NUP62,OAZ1,OSBPL1A,PAQR9,PCDH1,PCGF5,PDE9A,PDGFRB,PDP2,PDPK1,PER1,PFDN2,PGAP1,PHF20L1,PHKA2,PIK3AP1,PIK3CA,PKHD1,PLD1,PLXNA2,PNKD,PNRC1,PPP1R14B,PPP1R1B,PPP2R1A,PRPF39,PRPF6,PSMD8,PTBP1,QDPR,RABGAP1L,RASSF3,RB1CC1,RBBP5,RICTOR,ROCK1,RPA1,RUFY3,SAA4,SBF2,SCAPER,SCYL1,SEC63,SECISBP2L,SENP6,SESN3,SGK2,SHPRH,SLC1A4,SLC20A1,SLC20A2,SLC27A2,SLC2A2,SLC35B3,SLC38A2,SLC39A10,SLC39A8,SLC6A12,SMAD7,SMAP1,SNRNP200,SNX9,SOAT2,SPOP,SPTBN1,SPTBN2,STIP1,STX8,SULF2,SYAP1,SYNE1,SYT1,TBC1D16,TBC1D24,TBRG4,TCP1,TEP1,TIMM44,TK1,TMEM126A,TMEM41B,TMEM97,TNRC6A,TOP1MT,TPR,TTC1,TUBB,UBAC1,UBTF,ULK1,UNG,UROC1,UTRN,VPS13A,WDR18,WDR26,WDR81,WNT5B,ZCCHC24	
Lipid Metabolism,Small Molecule Biochemistry,Vitamin And Mineral Metabolism	Synthesis	Synthesis Of Steroid	1.13E-09	-1.709	ABCG5,ABCG8,ACAT2,ACLY,ACOX2,AKR1D1,ATP1A1,BAAT,CES1,CYP39A1,CYP7A1,ELOVL6,ESRRA,G6PD,HMGCR,HSD17B2,HSD3B7,IGFBP2,IL18,JAK2,KITLG,KPNB1,LSS,NPC1,NR1H4,NR5A2,OSBPL1A,POR,RAN,SERPINE2,SLC27A2,SLC9A3R1,SOAT2,STARD4	34
Organismal Survival	Morbidity Or Mortality	Morbidity Or Mortality	8.62E-11	-1.712	ABCB1,ABCB4,ACLY,ADH4,AGPAT2,AKAP9,ANXA7,AQP11,ARIH2,ATG3,ATP1A1,ATP1B1,BAP1,BID,BIK,BLMH,CAPN2,CBL	171

					B,CCBE1,CD2AP,CD47,CDA,CDH1,CDH2,CDO1,CEBPD,CITED2,CNN3,CNP,CPT1A,CSRNP1,CUL4B,CXADR,CYLD,CYP7A1,DCTN1,DHX9,DICER1,DMD,DMTF1,DNAJB1,DNAJC5,DSP,DVL1,E2F5,ENDOG,ERBB3,F11,F2R,F3,FADS2,FECH,FGA,FGFR2,FGFRL1,FGG,FH,FKBP4,FLCN,FOXO3,G6PD,GABARAP,GAS2L3,GCLC,GNA12,GPD2,GSS,HECTD3,HMGCR,HNRNPC,HNRNPD,HRAS,HSD17B2,HSD3B7,HSPB1,ID2,IGFBP2,IL18,ING4,ITPA,JAK2,KAT2B,KDM6A,KEAP1,KITLG,KLF9,LGR5,LIN7A,LIN7C,LMNA,LTBR,MACROH2A1,MAP1LC3B,MAP2K3,MAP3K7,MBNL2,MCOLN1,MEF2A,NCOA1,NEK7,NFKBIA,NINJ1,NIPBL,NPC1,NR1H4,NR3C2,NR5A2,NUDT1,NUP62,NUS1,OGA,PDCD2,PDGFC,PDGFRB,PDLIM1,PDPK1,PES1,PGAP1,PIK3CA,POR,PPIF,PPP1R12A,PPP1R9A,PPP2R1A,PRPF19,PSEN2,PSMG1,PTBP1,PTBP2,QDPR,RASA3,RB1CC1,RCAN1,RICTOR,RNF14,RUFY3,SCARB2,SCYL1,SDC4,SEC63,SERPINA6,SERPINH1,SFRP5,SLC20A1,SLC2A2,SLC2A9,SLC30A10,SLC39A10,SLC9A3R1,SMAD7,SOD2,SPOP,SPTBN1,STIP1,STK40,STXBP3,SULF2,SYNE1,SYT1,TK1,TNFAIP8L1,TSTA3,UBE2L3,UBR1,UBTF,ULK1,UNG,UROD,UTRN,UXS1,WNT5B	
Cancer,Organismal Injury And Abnormalities	Cancer	Cancer Of Secretory Structure	4.67E-26	-1.715	AASDH,ABCB1,ABCB4,ABCG2,ABCG5,ABCG8,ABTB2,ACAD8,ACAT2,ACLY,ACOX2,ACSL3,ACSL4,ACSL5,ACSM1,ACSM5,A	516

CSS2,ACTR6,ADH4,
AFG3L2,AGPAT2,AH
CY,AHNAK,AKAP11,
AKAP9,AKR1D1,ALA
S1,ALDH1L1,ALG13,
AMDHD1,ANKRD12,
ANP32A,ANXA7,ARG
LU1,ARHGAP18,ARH
GAP21,ARHGAP6,A
RIH2,ARL15,ARL4A,
ARL6IP4,ASAP2,ASB
13,ATG4D,ATP1A1,A
TP1B1,ATP6V1D,AT
XN2L,AUH,BAAT,BA
P1,BDH1,BHMT,BHM
T2,BID,BIK,BLMH,BR
D8,C11orf54,C9orf15
2,C9orf16,CA3,CAMK
K2,CAPN2,CARS1,C
BLB,CCBE1,CCDC66
,CCT4,CCT5,CCT6A,
CCT7,CD2AP,CD47,
CD99L2,CDA,CDC14
B,CDH1,CDH2,CDO1
,CEBPD,CENPV,CES
1,CGN,CHD6,CHPT1,
CITED2,CLDND1,CL
K2,CLMN,CLOCK,CM
TM6,CMTM8,CNN3,C
NOT4,CNOT6,CNP,C
OL27A1,COL5A3,CO
RO1B,CPT1A,CRIP2,
CRLS1,CROT,CSRN
P1,CTDSP1,CTPS2,
CTSH,CUL4B,CXAD
R,CYB5B,CYLD,CYP
2U1,CYP39A1,CYP4
F2,CYP7A1,CYTH1,D
AZAP1,DCTN1,DDC,
DDX60,DGKZ,DHX9,
DIAPH2,DICER1,DIO
1,DIPK2A,DIXDC1,D
MD,DMTF1,DNAJB1,
DOCK4,DPP4,DPP9,
DSP,DSTN,DTYMK,D
USP19,DVL1,E2F5,E
FHD2,EGR1,EIF1AX,
EIF4EBP1,ELF1,END
OG,ENPP1,ERBB3,E
SRRA,EXOC6,EXOS
C2,F11,F3,FADS2,FA
M102A,FAM118B,FA
M160B1,FAM76B,FA
M89A,FBXO31,FBXO
9,FECH,FGA,FGB,FG
FR2,FGFRL1,FGG,F
H,FKBP4,FLCN,FMO
1,FOXK1,FOXO3,FP
GS,FTSJ3,G6PD,GA
S2L3,GCAT,GCLC,G
CNT2,GDA,GNA12,G
OLGB1,GPD2,GPN1,

GRHPR,GSDMD,GS
K3A,GSS,GSTM2,GT
F2A2,GTF2F1,GTF3
C2,GYG1,HECA,HEC
TD3,HERC4,HLF,HM
G20B,HMGCR,HNRN
PC,HNRNPD,HOME
R2,HOOK1,HRAS,HS
D17B2,HSD3B7,HSP
B1,ICK,ID2,ID4,IGFB
P2,ING4,IRF6,IRS1,I
TPK1,JAK2,JMJD1C,
KAT2B,KCMF1,KDM6
A,KEAP1,KHK,KIAA0
100,KITLG,KLF12,KL
F9,KLHDC2,KLHL21,
KLHL7,KTN1,KYNU,L
2HGDH,LARS2,LGAL
S4,LGR5,LIN7A,LIN7
C,LITAF,LMNA,LPIN1
,LRIG1,LRP12,LRRC
28,LRRC3,LSM14B,L
SS,LTBR,MAGED1,M
AGI3,MAP3K7,MBNL
2,MCOLN1,MEF2A,M
IA2,MKNK2,MLLT10,
MLX,MMD,MOCOS,M
ON2,MPDZ,MPP1,M
PRIP,MRPL12,MRPL
20,MRPS22,MRPS25
,MRPS27,MRPS9,MT
1F,MYO1B,MYO5B,M
YO6,N4BP2,NAGK,N
COA1,NDUFA12,ND
UFA9,NEK7,NET1,NF
KBIA,NGEF,NHLRC2,
NINJ1,NIPBL,NME1,
NOSIP,NPC1,NR1H4,
NR2F6,NR5A2,NRIP1
,NT5C,NUDT19,NUD
T7,NUP62,NUP88,NU
S1,OAZ1,OSBPL1A,
OTUD6B,OXR1,PAL
MD,PAN2,PAN3,PAQ
R9,PCDH1,PCGF5,P
DCD2,PDE2A,PDE9A
,PDGFRB,PDLIM1,P
DP2,PDPK1,PDRG1,
PER1,PER3,PES1,P
EX26,PEX6,PFKFB1,
PGAP1,PHF20L1,PH
KA2,PIK3AP1,PIK3C
A,PKHD1,PLD1,PLX
NA2,PMPCA,PNKD,P
NRC1,POR,PPIF,PP
OX,PPP1R12A,PPP1
R1B,PPP1R3B,PPP1
R9A,PPP2R1A,PRDX
6,PRPF19,PRPF39,P
RPF6,PRPS1,PRPS2
,PSEN2,PSMA6,PSM
D1,PSMD11,PSMD2,

PSME2,PSMF1,PSM
G1,PSPC1,PSPH,PT
BP1,PTBP2,PXMP4,
QDPR,RAB11FIP2,R
AB1B,RABGAP1L,RA
I14,RALGPS2,RASA3
,RASSF3,RB1CC1,R
BBP5,RBBP7,RBKS,
RCAN1,RCL1,REPIN
1,RHBDD1,RHOD,RI
CTOR,RNF14,RNF14
4A,ROCK1,RPA1,RPI
A,RUFY3,SAA4,SBF2
,SCAPER,SCARB2,S
CYL1,SDC4,SDR42E
1,SEC63,SECISBP2L
,SENP6,SERPINA6,S
ERPINE2,SERPINH1,
SESN3,SETDB2,SFR
P5,SGK2,SGTB,SH3
D19,SHMT1,SHPRH,
SIRT5,SLC10A1,SLC
16A5,SLC1A4,SLC20
A2,SLC25A32,SLC27
A2,SLC2A5,SLC2A9,
SLC35A3,SLC35B1,S
LC35B3,SLC39A10,S
LC39A8,SLC6A12,SL
C9A3R1,SLK,SMAD7
,SMAP1,SNAPC2,SN
RNP200,SNTB1,SNX
5,SNX9,SOAT2,SOD
2,SPARC,SPON2,SP
OP,SPTBN1,SPTBN2
,ST3GAL4,STARD7,S
TIP1,STK40,STOM,S
TT3B,STX18,STX8,S
TXBP3,SULF2,SUMF
1,SYAP1,SYNE1,SYT
1,TBC1D16,TBC1D20
,TBC1D24,TBRG4,TC
P1,TEP1,TEX2,THAP
4,TIFA,TIMM10,TIMM
44,TK1,TLCD4,TLE1,
TMEM126A,TMEM41
A,TMEM51,TMEM62,
TNFAIP2,TNFAIP8L1,
TNRC6A,TOP1MT,TP
R,TPRG1L,TPRKB,T
RIB3,TRMT1,TSPAN
33,TSTA3,TTC1,TTC
19,TUBB,TUBB6,TUB
GCP2,TXN2,TXNL1,
UBAC1,UBE2F,UBE2
L3,UBR1,UBTF,UBX
N6,UCK1,UGP2,ULK
1,UNG,UPP2,UROC1
,UROD,USP47,UTP1
4A,UTRN,VPS13A,W
DR18,WDR26,WDR8
1,WIPI2,WNT5B,YPE
L3,ZCCHC24,ZFAND

					2B,ZKSCAN1,ZNF23,ZNF326,ZNF654	
Cancer,Hematological Disease,Organismal Injury And Abnormalities	Hematologic Cancer	Hematologic Cancer	1.18E-05	-1.722	ABCB1,ABCB4,ABCG2,ABCG5,ABCG8,ACSL4,AHNAK,AKAP11,ALAS1,ANXA7,ASAP2,ATG4D,ATP1A1,ATP1B1,BAAT,BAP1,BHMT,CAPN2,CBLB,CCT3,CCT7,CD47,CDA,CDH1,CDO1,CEBPD,CHD6,CITED2,CNP,COL27A1,CPT1A,CTPS2,CTSH,CUL4B,DAZAP1,DDC,DDX60,DEK,DGKZ,DHX9,DICER1,DIO1,DIPK2A,DMD,DMTF1,DNAJC5,DPP4,DSP,ECHDC3,EGR1,EIF4EBP1,ENPP1,ERBB3,EXTL2,F2R,F3,FAM102A,FBXO31,FBXO9,FGFR2,FGG,FOXO3,FPGS,GNA12,GPD2,GRHPR,GYG1,HECA,HECTD3,HMG20B,HMGCR,HNRNPD,HOOK1,HRAS,HSPB1,ICK,ID2,ID4,IL18,IRS1,JAK2,JMJD1C,KDM6A,KIAA0100,KITLG,KPNB1,LARS2,LGALS4,LMNA,LPIN1,LRIG1,LRRC3,LTBR,MAGED1,MAP2K3,MEF2A,MIA2,MKNK2,MLLT10,MPDZ,MRPL20,MRPL44,MYO5B,NET1,NFKBIA,NINJ1,NIPBL,NME1,NRIP1,NT5C,NUP88,OSBPL1A,PALMD,PAN3,PAQR9,PDE9A,PDGFC,PDGFRB,PDPK1,PER1,PER3,PES1,PEX6,PIK3AP1,PIK3CA,PKHD1,PLXNA2,PNKD,PNRC1,POLE4,POR,PPM1A,PPP1R14B,PPP1R1B,PPP1R3B,PRPF19,PRPS1,PSEN2,PSMD1,PSMD2,PSME2,RAN,RHOD,RICTOR,RPA1,SARS1,SCARB2,SERPINH1,SGK2,SHMT1,SHPRH,SLC25A32,SMAD7,SOAT2,SOD2,SPARC,SPTBN1,STIP1,STK24,STK40,SYNE1,SYT1,TCIM,TEP1,TPI1,TPR,TUBB6,TU	175

					T4,UBE2F,UBR1,UBXN1,UGT2B10,UNG,UROC1,VPS13A	
Cancer,Organismal Injury And Abnormalities,Reproductive System Disease	Breast Or Gynecological Cancer	Breast Or Gynecological Cancer	3.24E-06	-1.731	ABCB1,ABCB4,ABCG2,ABCG5,ACAD8,ACOX2,ACSL3,ACSL4,ACSM1,ACSS2,AGPAT2,AHNAK,AKAP11,AKAP9,ALAS1,ALDH1L1,ALG13,ANKRD12,ARHGAP21,ARHGAP6,ARIH2,ARL15,ATP1A1,AUH,BAP1,BDH1,BIK,BRD8,CAPN2,CCT3,CCT5,CCT6A,CD2AP,CD47,CD9,CD99L2,CDA,CDH1,CDH2,CDO1,CEBPD,CES1,CGN,CLK2,CLMN,CMTM8,CNOT4,CNOT6,COL27A1,CPEB4,CPT1A,CS,CTSH,CXADR,CYLD,CYP2U1,CYP4F2,CYP7A1,DAZAP1,DCTN1,DDX60,DGKZ,DHX9,DIAPH2,DICER1,DIO1,DIPK2A,DIXDC1,DMD,DOCK4,DPP4,DPP9,DSP,DSTN,E2F5,EGR1,EIF1AX,EIF4EBP1,ELF1,ELOVL6,ENPP1,ERBB3,ESRRA,EXOC6,EXOSC2,F11,F2R,F3,FADS2,FAM160B1,FAM172A,FECH,FGA,FGFR2,FGG,FOXO3,FTSJ3,GNA12,GOLGB1,GRHPR,GSK3A,GSTM2,GYG1,HECA,HECTD3,HLF,HMGCR,HRAS,HSPB1,ICK,IGFBP2,IL18,ING4,IRF6,IRS1,ITPA,JAK2,JMJD1C,KAT2B,KCMF1,KDM6A,KEAP1,KIAA0100,KLF9,KTN1,L2HGDH,LGALS4,LGR5,LMNA,LRIG1,LSM14B,MACROH2A1,MAGED1,MAGI3,MAP1LC3B,MAP2K3,MCOLN1,MEF2A,MKNK2,MON2,MPDZ,MPRIP,MRPL24,MRPL34,MRPL44,MRPS22,MRPS27,MT1F,MYO1B,MYO5B,MYO6,NCOA1,NDUFA9,NDUFB9,NEK7,NHLRC2,NIPBL,NME1,NOSIP,NPC1,NR2F6,NR3C2,	274

					NR5A2,NRIP1,NUP62,NUS1,OSBPL1A,OXR1,PALMD,PAN3,PCDH1,PCGF5,PDCD2,PDE2A,PDGFRB,PDLIM1,PDP2,PER3,PGAP1,PHF20L1,PHKA2,PIK3CA,PKHD1,PLD1,PLXNA2,PNRC1,POR,PPIF,PPP1R1B,PPP1R9A,PPP2R1A,PRPF39,PRPF6,PSMD2,PTBP1,PTBP2,RABEPK,RABGAP1L,RALGPS2,RASA3,RB1CC1,RBBP5,RBBP7,RGS2,RICTOR,RNF14,ROCK1,RPIA,SBF2,SCAPER,SCARB2,SECISBP2L,SESN3,SETDB2,SFRP5,SLC20A1,SLC20A2,SLC25A32,SLC2A2,SLC38A2,SLC39A10,SLC9A3R1,SNRNP200,SNTB1,SNX10,SOD2,SPARC,SPG21,SPOP,SPTBN1,SPTBN2,STARD7,STK24,STK40,STOM,STT3B,STX8,SYNE1,TBC1D20,TCP1,TEP1,TEX2,THRSP,TIGD2,TIMM44,TK1,TLE1,TMEM126A,TMEM97,TNFAIP2,TNRC6A,TOP1MT,TPI1,TPR,TPRG1L,TRIB3,TTC1,TUBB,TUBB6,TXNL1,UGP2,UGT2B10,UROD,USP47,UTRN,UXS1,VPS13A,WNT5B,YWHAH,ZCCHC24,ZFAND2B,ZKSCAN1,ZNF23,ZNF32	
Cancer,Organismal Injury And Abnormalities	Lymphoid Cancer	Lymphoid Cancer	8.05E-05	-1.757	ABCB1,ABCG2,AHNAK,AKAP11,ANXA7,ASAP2,ATP1B1,BAP1,CBLB,CCT3,CCT7,CD47,CDH1,CDO1,CEBPD,CHD6,CITED2,CNP,COL27A1,CTSH,CUL4B,DDC,DEK,DGKZ,DICER1,DIO1,DMD,DMTF1,DPP4,DSP,EGR1,EIF4EBP1,ENPP1,ERBB3,EXTL2,F2R,F3,FAM102A,FBXO31,FBXO9,FGFR2,FOXO3,FPGS,GNA12,GPD2,GRHPR,HECA,HMGCR,HRAS,HSPB1,ICK,ID2,IL18,JAK2	113

					,JMJD1C,KDM6A,KIAA0100,KITLG,KPNB1,LPIN1,LRIG1,LTBR,MAGED1,MAP2K3,MEF2A,MIA2,MKNK2,MLLT10,MPDZ,MRPL20,MYO5B,NFKBIA,NINJ1,NIPBL,NME1,PALMD,PDE9A,PDGFC,PDGFRB,PDPK1,PER1,PIK3CA,PLXNA2,PNKD,PNRC1,POLE4,POR,PRPF19,PRPS1,PSMD1,PSMD2,RAN,RICTOR,RPA1,SARS1,SERPINH1,SHMT1,SLC25A32,SMAD7,SOD2,SPARC,SPTBN1,STIP1,STK24,SYT1,TBC1D16,TPR,TUBB6,TUT4,UBE2F,UBR1,UNG,VPS13A	
Cancer,Organismal Injury And Abnormalities	Breast Or Pancreatic Cancer	Breast Or Pancreatic Cancer	7.45E-04	-1.762	AASDH,ABCB1,ABCB4,ABCG2,ABTB2,ACAT2,ACLY,ACSM5,ACSS2,ADH4,AGPAT2,AHNAK,AKAP11,AKAP9,ALDH1L1,ALG13,ANKRD12,ANP32A,ATP1A1,AUH,BAP1,BDH1,BRD8,C9orf152,CAMKK2,CAPN2,CBLB,CCDC66,CCT3,CCT5,CCT6A,CD47,CD9,CDH1,CDH2,CDO1,CEBPD,CES1,CHD6,CLK2,CMTM8,CNOT4,CNOT6,COL27A1,COL5A3,CPEB4,CPT1A,CS,CUL4B,CXADR,CYLD,CYP2U1,DAZAP1,DCTN1,DGKZ,DHX9,DIAPH2,DICER1,DIXDC1,DMD,DOCK4,DPP4,DSP,DSTN,E2F5,EGR1,EIF1AX,EIF4EBP1,ELOVL6,ERBB3,ESRRA,EXOC6,EXOSC2,F11,F2R,F3,FADS2,FGA,FGFR2,FOXO3,FTSJ3,GAS2L3,GCLC,GNA12,GOLGB1,GRHPR,GSTM2,GYG1,HERC4,HMGCR,HRAS,HSPB1,IGFBP2,IL18,ING4,IRF6,IRS1,ITPA,JAK2,JMJD1C,KAT2B,KDM6A,KEAP1,KIAA0100,KLF12,KLF9,LGALS4,LGR5,LMNA,LRIG1,LSM14B,LSS,MACR	232

					OH2A1,MAGI3,MAP1LC3B,MAP2K3,MEF2A,MIA2,MKNK2,MPDZ,MRPL24,MRPL34,MRPS27,MT1F,MYO1B,MYO5B,MYO6,NAGK,NDUFB9,NEK7,NGEF,NINJ1,NIPBL,NME1,NPC1,NR3C2,NR5A2,NRIP1,NUS1,OSBPL1A,PAN2,PAQR9,PCDH1,PDGFRB,PGAP1,PHF20L1,PHKA2,PIK3CA,PKHD1,PLXNA2,PNRC1,POR,PPP1R1B,PPP1R9A,PPP2R1A,PRPF39,PRPF6,PRPS1,PSMD1,PSMD11,PSMF1,PTBP1,PXMP4,RABEPK,RABGAP1L,RASA3,RB1CC1,RBBP5,RGS2,RICTOR,RNF14,ROCK1,RPA1,SBF2,SCAPER,SEC63,SECISBP2L,SERPINE2,SERPINH1,SETDB2,SFRP5,SHPRH,SLC20A1,SLC20A2,SLC25A32,SLC2A5,SLC2A9,SLC9A3R1,SNRNP200,SNTB1,SOD2,SPARC,SPG21,SPOP,SPTBN1,SPTBN2,ST3GAL4,STARD7,STK40,STOM,STT3B,STX8,SYNE1,TCP1,TEP1,TEX2,THRSP,TIMM44,TK1,TMEM126A,TMEM97,TNRC6A,TOP1MT,TPI1,TPR,TRIB3,TTC1,TUBB,TUBB6,TUBGCP2,TXNL1,UGP2,UROD,USP47,UTRN,UXS1,VPS13A,WNT5B,YWHAH,ZFAND2B,ZNF23,ZNF654	
Cancer,Organismal Injury And Abnormalities	Genitourinary Tumor	Genitourinary Tumor	1.88E-12	-1.806	ABCB1,ABCB4,ABCG2,ABCG5,ACAD8,ACLY,ACOX2,ACSL3,ACSL4,ACSM1,ACSS2,AFG3L2,AGPAT2,AHNAK,AKAP11,AKAP9,ALAS1,ALDH1L1,ALG13,ANKRD12,ARHGAP18,ARHGAP21,ARHGAP6,ARIH2,ARL15,ASAP2,ATP1A1,ATP1B1,ATP6V1D,ATXN2L,AUH,BAAT,BAP1,BDH1,BIK,BLMH,BRD8,CA3,CAPN2,C	398

ARS1,CBLB,CCT3,C
CT5,CCT6A,CD2AP,
CD47,CD9,CD99L2,C
DA,CDC14B,CDH1,C
DH2,CDO1,CEBPD,C
ES1,CGN,CHD6,CLD
ND1,CLK2,CLMN,CM
TM8,CNOT4,CNOT6,
CNP,COL27A1,COL5
A3,CPEB4,CPT1A,C
ROT,CS,CSRNP1,CT
SH,CXADR,CYLD,CY
P2U1,CYP4F2,CYP7
A1,CYTH1,DAZAP1,
DCTN1,DDC,DDX60,
DGKZ,DHX9,DIAPH2,
DICER1,DIO1,DIPK2
A,DIXDC1,DMD,DMT
F1,DOCK4,DPP4,DP
P9,DSP,DSTN,DUSP
19,DVL1,E2F5,EFHD
2,EGR1,EIF1AX,EIF4
EBP1,ELF1,ELOVL6,
ENPP1,ERBB3,ESRR
A,EXOC6,EXOSC2,E
XTL2,F11,F2R,F3,FA
DS2,FAM160B1,FAM
172A,FAM89A,FECH,
FGA,FGFR2,FGFRL1
,FGG,FH,FKBP4,FLC
N,FOXK1,FOXO3,FT
SJ3,G6PD,GABARAP
,GDA,GNA12,GOLGB
1,GPD2,GRHPR,GSD
MD,GSK3A,GSTM2,
GTF2A2,GTF3C2,GY
G1,HECA,HECTD3,H
LF,HMGCR,HRAS,H
SD3B7,HSPB1,ICK,I
D2,IGFBP2,IL18,ING
4,IRF6,IRS1,ITPA,JA
K2,JMJD1C,KAT2B,K
CMF1,KDM6A,KEAP
1,KIAA0100,KITLG,K
LF9,KLHDC2,KLHL7,
KTN1,KYNU,L2HGD
H,LARS2,LGALS4,LG
R5,LIN7C,LITAF,LMN
A,LPIN1,LRIG1,LRP1
2,LRRC28,LSM14B,L
SS,LTBR,MACROH2
A1,MAGED1,MAGI3,
MAP1LC3B,MAP2K3,
MBNL2,MCOLN1,ME
F2A,MIA2,MKNK2,ML
LT10,MLX,MMD,MOC
OS,MON2,MPDZ,MP
P1,MPRIP,MRPL24,
MRPL34,MRPL44,MR
PS22,MRPS25,MRPS
27,MT1F,MYO1B,MY
O5B,MYO6,N4BP2,N

4BP2L1,NAGK,NCOA
1,NDUFA9,NDUFB9,
NEK7,NFKBIA,NGEF,
NHLRC2,NINJ1,NIPB
L,NME1,NOSIP,NPC
1,NR2F6,NR3C2,NR5
A2,NRIP1,NUDT7,NU
P62,NUP88,NUS1,O
AZ1,OSBPL1A,OTUD
6B,OXR1,PALMD,PA
N3,PCDH1,PCGF5,P
DCD2,PDE2A,PDGF
RB,PDLIM1,PDP2,PD
PK1,PDRG1,PER1,P
ER3,PEX6,PGAP1,P
HF20L1,PHKA2,PIK3
CA,PKHD1,PLD1,PL
XNA2,PNKD,PNRC1,
POR,PPIF,PPP1R1B,
PPP1R3B,PPP1R9A,
PPP2R1A,PRPF39,P
RPF6,PSEN2,PSMA6
,PSMD1,PSMD2,PTB
P1,PTBP2,PXMP4,R
AB11FIP2,RABEPK,R
ABGAP1L,RAI14,RAL
GPS2,RASA3,RB1CC
1,RBBP5,RBBP7,RC
AN1,RCL1,REPIN1,R
GS2,RHOD,RICTOR,
RNF14,RNF144A,RO
CK1,RPIA,SBF2,SCA
PER,SCARB2,SCYL1
,SDC4,SDR42E1,SE
C63,SECISBP2L,SEN
P6,SERPINA6,SERPI
NE2,SESN3,SETDB2
,SFRP5,SHPRH,SIRT
5,SLC16A5,SLC1A4,
SLC20A1,SLC20A2,S
LC25A32,SLC2A2,SL
C2A5,SLC2A9,SLC38
A2,SLC39A10,SLC39
A8,SLC6A12,SLC9A3
R1,SLK,SMAD7,SMA
P1,SNRNP200,SNTB
1,SNX10,SOD2,SPA
RC,SPG21,SPOP,SP
TBN1,SPTBN2,ST3G
AL4,STARD7,STIP1,
STK24,STK40,STOM,
STT3B,STX8,STXBP
3,SULF2,SYNE1,TBC
1D16,TBC1D20,TBC1
D24,TCP1,TEP1,TEX
2,THRSP,TIFA,TIGD2
,TIMM44,TK1,TLE1,T
MEM126A,TMEM51,T
MEM97,TNFAIP2,TN
RC6A,TOP1MT,TPI1,
TPR,TPRG1L,TRIB3,
TRMT1,TSPAN33,TT

					C1,TUBB,TUBB6,TXNL1,UBAC1,UBE2F,UBR1,UGP2,UGT2B10,UROC1,UROD,USP47,UTP14A,UTRN,UXS1,VPS13A,WIPI2,WNT5B,YPEL3,YWHAH,ZCCHC24,ZFAND2B,ZKSCAN1,ZNF23,ZNF32,ZNF654	
Cancer,Hematological Disease,Immunological Disease,Organismal Injury And Abnormalities	Lymphocytic Cancer	Lymphocytic Cancer	7.99E-05	-1.815	ABCB1,ABCG2,AHNAK,AKAP11,ANXA7,ASAP2,ATP1B1,BAP1,CBLB,CCT3,CCT7,CD47,CDH1,CDO1,CEBPD,CHD6,CITED2,CNP,COL27A1,CTSH,CUL4B,DDC,DEK,DGKZ,DICER1,DIO1,DMD,DMTF1,DPP4,DSP,EGR1,EIF4EBP1,ENPP1,ERBB3,EXTL2,F2R,F3,FAM102A,FBXO31,FBXO9,FGFR2,FOXO3,FPGS,GNA12,GPD2,GRHPR,HECA,HMGCR,HRAS,HSPB1,ICK,ID2,IL18,JAK2,JMJD1C,KDM6A,KIAA0100,KITLG,KPNB1,LPIN1,LRIG1,LTBR,MAGED1,MAP2K3,MEF2A,MIA2,MKNK2,MLLT10,MPDZ,MRPL20,MYO5B,NFKBIA,NINJ1,NIPBL,NME1,PALMD,PDE9A,PDGFC,PDGFRB,PDPK1,PER1,PIK3CA,PLXNA2,PNKD,PNRC1,POLE4,POR,PRPF19,PRPS1,PSMD1,PSMD2,RAN,RICTOR,RPA1,SARS1,SERPINH1,SHMT1,SLC25A32,SMAD7,SOD2,SPARC,SPTBN1,STIP1,STK24,SYT1,TPR,TUBB6,TUT4,UBE2F,UBR1,UNG,VPS13A	112
Cancer,Hematological Disease,Organismal Injury And Abnormalities	Lymphocytic Neoplasm	Lymphocytic Neoplasm	8,46E-05	-1,815	ABCB1,ABCG2,AHNAK,AKAP11,ANXA7,ASAP2,ATP1B1,BAP1,CBLB,CCT3,CCT7,CD47,CDH1,CDO1,CEBPD,CHD6,CITED2,CNP,COL27A1,CTSH,CUL4B,DDC,DEK,DGKZ,DICER1,DIO1,DMD,DMTF1,DPP4,DSP,EGR1,EIF4EBP1,ENPP1,ERBB3,EXTL2,F	112

					2R,F3,FAM102A,FBXO31,FBXO9,FGFR2,FOXO3,FPGS,GNA12,GPD2,GRHPR,HECA,HMGCR,HRAS,HSPB1,ICK,ID2,IL18,JAK2,JMJD1C,KDM6A,KIAA0100,KITLG,KPNB1,LPIN1,LRIG1,LTBR,MAGED1,MAP2K3,MEF2A,MIA2,MKNK2,MLLT10,MPDZ,MRPL20,MYO5B,NFKBIA,NINJ1,NIPBL,NME1,PALMD,PDE9A,PDGFC,PDGFRB,PDPK1,PER1,PIK3CA,PLXNA2,PNKD,PNRC1,POLE4,POR,PRPF19,PRPS1,PSMD1,PSMD2,RAN,RICTOR,RPA1,SARS1,SERPINH1,SHMT1,SLC25A32,SMAD7,SOD2,SPARC,SPTBN1,STIP1,STK24,SYT1,TPR,TUBB6,TUT4,UBE2F,UBR1,UNG,VPS13A	
Organismal Survival	Organismal Death	Organismal Death	5.50E-11	-1.818	ABCB1,ABCB4,ACLY,ADH4,AGPAT2,AKAP9,ANXA7,AQP11,ARIH2,ATG3,ATP1A1,ATP1B1,BAP1,BID,BIK,BLMH,CAPN2,CBLB,CCBE1,CD2AP,CD47,CDA,CDH1,CDH2,CDO1,CEBPD,CITED2,CNN3,CNP,CPT1A,CSRNP1,CUL4B,CXADR,CYLD,CYP7A1,DCTN1,DHX9,DICER1,DMD,DMTF1,DNAJB1,DNAJC5,DSP,DVL1,E2F5,ENDOG,ERBB3,F11,F2R,F3,FADS2,FECH,FGA,FGFR2,FGFRL1,FGG,FH,FKBP4,FLCN,FOXO3,G6PD,GABARAP,GAS2L3,GCLC,GNA12,GPD2,GSS,HMGCR,HNRNPC,HNRNPD,HRAS,HSD17B2,HSD3B7,HSPB1,ID2,IGFBP2,IL18,ING4,ITPA,JAK2,KAT2B,KDM6A,KEAP1,KITLG,KLF9,LGR5,LIN7A,LIN7C,LMNA,LTBR,MACROH2A1,MAP1LC3B,MAP2K3,MAP3K7,MBNL2,MCOLN1,MEF2A,NCOA1,N	170

					EK7,NFKBIA,NINJ1,NIPBL,NPC1,NR1H4,NR3C2,NR5A2,NUDT1,NUP62,NUS1,OGA,PDCD2,PDGFC,PDGFRB,PDLIM1,PDPK1,PES1,PGAP1,PIK3CA,POR,PPIF,PPP1R12A,PPP1R9A,PPP2R1A,PRPF19,PSEN2,PSMG1,PTBP1,PTBP2,QDPR,RASA3,RB1CC1,RCAN1,RICTOR,RNF14,RUFY3,SCARB2,SCYL1,SDC4,SEC63,SERPINA6,SERPINH1,SFRP5,SLC20A1,SLC2A2,SLC2A9,SLC30A10,SLC39A10,SLC9A3R1,SMAD7,SOD2,SPOP,SPTBN1,STIP1,STK40,STXBP3,SULF2,SYNE1,SYT1,TK1,TNFAIP8L1,TSTA3,UBE2L3,UBR1,UBTF,ULK1,UNG,UROD,UTRN,UXS1,WNT5B	
Carbohydrate Metabolism,Cellular Function And Maintenance,Molecular Transport,Small Molecule Biochemistry	Transport	Transport Of D-Glucose	5.20E-07	-1.867	DGKZ,ENPP1,ERBB3,GSK3A,IRS1,MAP2K3,MEF2A,OGA,PDGFRB,PDPK1,PIK3CA,PPP1R3B,REPIN1,ROCK1,SLC2A2,SLC2A5,SLC2A9,STXBP3,TRIB3	19
Cancer,Gastrointestinal Disease,Organismal Injury And Abnormalities	Hepatobiliary Carcinoma	Hepatobiliary Carcinoma	8.30E-06	-1.885	ABCB4,ABCG8,ACLY,ACOX2,ACSS2,AHNAK,AKAP11,AKAP9,AKR1D1,AMDHD1,ANKRD12,ANXA7,ARGLU1,ARHGAP21,ARHGAP6,ASAP2,ASB13,ATP1A1,ATP1B1,ATXN2L,BAAT,BAP1,BDH1,BHMT,BUD23,CARS1,CBLB,CCT6A,CD47,CDH1,CDH2,CHD6,CIB3,CLK2,CLMN,CMTM6,CNOT4,COL27A1,COL5A3,CPEB4,CRIP2,CROT,CS,CTPS2,CUL4B,CXADR,CYP39A1,CYP7A1,CYTH1,DDC,DEK,DIAPH2,DICER1,DIPK2A,DMD,DOCK4,DPP4,DPP9,E2F5,ENPP1,ERBB3,ESRRA,EXTL2,F3,FAM160B1,FBXO9,FGA,FGB,FGFR2,FGFRL1,FGG,FLCN,F	235

OXK1,G6PD,GAS2L3
,GCLC,GNA12,GOLG
B1,GPD2,GRHPR,GS
DMD,GTF3C2,HECA,
HLF,HMGCR,HRAS,I
D4,IL18,IRS1,JMJD1
C,KDM6A,KEAP1,KIA
A0100,KLHDC2,KPN
B1,KTN1,KYNU,LAR
S2,LGR5,LIN7A,LMN
A,LTBR,MACROH2A
1,MAGI3,MAP1LC3B,
MAP2K3,MAP3K7,M
EF2A,MON2,MPDZ,M
PRIP,MRNIP,MRPL2
4,MRPS22,MYO6,NC
ALD,NET1,NGEF,NIP
BL,NME1,NPC1,NR1
H4,NR2F6,NR3C2,N
RIP1,NT5C,OSBPL1
A,OXR1,PAN2,PAN3,
PAQR9,PCDH1,PDE
2A,PDGFC,PDGFRB,
PER1,PEX26,PGAP1,
PHF20L1,PIK3AP1,PI
K3CA,PKHD1,PLD1,
PLXNA2,PMPCA,PO
R,PPM1A,PPOX,PPP
1R1B,PPP1R9A,PPP
2R1A,PRPF6,PSMD1
,PSMD11,PSMD2,PS
PH,PTBP2,RABGAP1
L,RALGPS2,RASA3,
RB1CC1,RBBP5,RCL
1,RICTOR,RNF167,R
OCK1,RUFY3,SAA4,
SARS1,SCAPER,SD
C4,SECISBP2L,SEN
P6,SERPINA6,SESN
3,SFRP5,SGK2,SGT
B,SIRT5,SLC10A1,SL
C1A4,SLC20A1,SLC2
0A2,SLC2A2,SLC2A5
,SLC35B3,SLC38A2,
SLC39A10,SLC39A8,
SLC6A12,SLK,SNRN
P200,SNTB1,SNX9,S
OAT2,SPTBN1,SPTB
N2,STK24,STK40,SU
LF2,SUMF1,SYNE1,T
BC1D16,TCIM,TEP1,
TEX2,THAP4,TIGD2,
TIMM44,TLE1,TMEM
219,TMEM41A,TMEM
62,TNFAIP8L1,TNRC
6A,TOP1MT,TPI1,TP
R,TSN,TSPAN33,TT
C1,TUBB,TUBGCP2,
TXNL1,UBAC1,UBXN
1,UBXN6,UCK1,UGP
2,UGT2B10,UPP2,UR
OC1,USP47,YWHAH,

					ZCCHC24	
Cancer,Gastrointestinal Disease,Hepatic System Disease,Organismal Injury And Abnormalities	Liver Carcinoma	Liver Carcinoma	1.04E-05	-1.885	ABCB4,ABCG8,ACLY,ACOX2,ACSS2,AHNAK,AKAP9,AKR1D1,AMDHD1,ANKRD12,ANXA7,ARGLU1,ARHGAP21,ARHGAP6,ASAP2,ASB13,ATP1A1,ATP1B1,ATXN2L,BAP1,BDH1,BHMT,BUD23,CARS1,CBLB,CCT6A,CD47,CDH1,CDH2,CHD6,CIB3,CLK2,CLMN,CMTM6,CNOT4,COL27A1,CPEB4,CRIP2,CROT,CXADR,CYP39A1,CYP7A1,CYTH1,DDC,DIAPH2,DICER1,DIPK2A,DMD,DOCK4,DPP4,DPP9,E2F5,ENPP1,ERBB3,ESRRA,EXTL2,F3,FAM160B1,FBXO9,FGA,FGB,FGFR2,FGG,FLCN,FOXK1,G6PD,GAS2L3,GCLC,GNA12,GOLGB1,GPD2,GRHPR,GSDMD,GTF3C2,HECA,HLF,HMGCR,HRAS,ID4,IL18,IRS1,JMJD1C,KDM6A,KEAP1,KIAA0100,KLHDC2,KPNB1,KTN1,KYNU,LARS2,LGR5,LMNA,LTBR,MACROH2A1,MAGI3,MAP1LC3B,MAP2K3,MAP3K7,MEF2A,MON2,MPDZ,MPRIP,MRNIP,MRPL24,MRPS22,MYO6,NCALD,NET1,NGEF,NIPBL,NME1,NPC1,NR1H4,NR2F6,NR3C2,NRIP1,NT5C,OSBPL1A,OXR1,PAN2,PAN3,PAQR9,PCDH1,PDE2A,PDGFC,PDGFRB,PER1,PEX26,PGAP1,PHF20L1,PIK3AP1,PIK3CA,PKHD1,PLD1,PLXNA2,PMPCA,POR,PPM1A,PPOX,PPP1R1B,PPP1R9A,PPP2R1A,PRPF6,PSMD1,PSMD11,PSMD2,PSPH,PTBP2,RABGAP1L,RALGPS2,RASA3,RB1CC1,RBBP5,RCL1,RICTOR,RNF167,ROCK1,RUFY3,SAA4,SARS1,SCAPER,SDC4,SECISBP2L,SEN	223

					P6,SESN3,SFRP5,SGK2,SGTB,SIRT5,SLC10A1,SLC1A4,SLC20A2,SLC2A2,SLC2A5,SLC35B3,SLC38A2,SLC39A10,SLC39A8,SLC6A12,SLK,SNRNP200,SNX9,SOAT2,SPTBN1,SPTBN2,STK24,STK40,SULF2,SUMF1,SYNE1,TBC1D16,TCIM,TEP1,TEX2,THAP4,TIGD2,TIMM44,TLE1,TMEM219,TMEM41A,TMEM62,TNFAIP8L1,TNRC6A,TOP1MT,TPI1,TPR,TSN,TSPAN33,TTC1,TUBB,TUBGCP2,TXNL1,UBAC1,UBXN1,UBXN6,UCK1,UGP2,UGT2B10,UPP2,UROC1,USP47,YWHAH,ZCCHC24	
Cancer,Gastrointestinal Disease,Hepatic System Disease,Organismal Injury And Abnormalities	Hepatocellular Carcinoma	Hepatocellular Carcinoma	5.12E-05	-1.885	ABCB4,ACOX2,ACSS2,AKR1D1,ANXA7,ATP1A1,ATP1B1,BHMT,BUD23,CDH1,CYP39A1,E2F5,ERBB3,F3,FAM160B1,FGA,FGB,FGFR2,FGG,GRHPR,HMGCR,HRAS,IL18,IRS1,KDM6A,KEAP1,LGR5,LTBR,MACROH2A1,MYO6,NME1,NR1H4,NRIP1,OXR1,PDGFC,PDGFRB,PIK3CA,POR,PRPF6,SAA4,SCAPER,SENP6,SFRP5,SIRT5,SLC10A1,SLC1A4,SOAT2,SPTBN1,SULF2,SYNE1,TCIM,TIGD2,TLE1,TMEM41A,TMEM62,TNFAIP8L1,TOP1MT,TPI1,TUBB,UROC1	60
Lipid Metabolism,Small Molecule Biochemistry	Synthesis	Synthesis Of Lipid	1.95E-09	-1.892	ABCB1,ABCG5,ABCG8,ACAT2,ACLY,ACOX2,ACSL3,ACSL4,ACSL5,ACSM1,ACSS2,ADH4,AGPAT2,AKR1D1,ALG13,ARF3,ATP1A1,BAAT,BHMT,CAMKK2,CD9,CES1,CHPT1,CRLS1,CYP39A1,CYP4F2,CYP7A1,DGKZ,DPP4,EGR1,EIF4EBP1,ELOVL6,ESRRA,F2R,FADS2,FOXO3,G6PD,HMGCR,HOMER2,HSD17B2,HSD3B7,IGFBP2,IL1	71

					8,JAK2,KITLG,KPNB1,LPIN1,LSS,NFKBIA,NPC1,NR1H4,NR5A2,NUS1,OSBPL1A,PDGFRB,PGAP1,PIK3CA,PLD1,POR,PRPF19,RAN,RGS2,SERPINE2,SLC27A2,SLC9A3R1,SOAT2,ST3GAL4,STARD4,STARD7,THRSP,TRIB3	
Cancer,Organismal Injury And Abnormalities	Development	Development Of Carcinoma	1.39E-11	-1.893	AASDH,ABCB1,ABCB4,ABCG5,ACAD8,ACLY,ACOX2,ACSL3,ACSL4,ACSM1,ACSS2,ACTR6,AFG3L2,AGPAT2,AHNAK,AKAP11,AKAP9,ALAS1,ALDH1L1,ALG13,ANKRD12,ANP32A,ANXA7,ARHGAP21,ARHGAP6,ARIH2,ARL15,ARL6IP4,ASAP2,ASB13,ATG3,ATG4D,ATXN2L,AUH,BAAT,BAP1,BDH1,BHMT,BRD8,BUD23,C11orf54,C9orf152,CAPN2,CARS1,CBLB,CCDC66,CCT3,CCT5,CCT6A,CCT7,CD2AP,CD47,CD9,CD99L2,CDC14B,CDH1,CDH2,CDO1,CES1,CGN,CHCHD3,CHD6,CHPT1,CHRAC1,CLDND1,CLK2,CLMN,CLOCK,CMTM6,CMTM8,CNOT4,CNOT6,CNP,COL27A1,COL5A3,CPEB4,CPT1A,CS,CTDSP1,CTPS2,CUL4B,CXADR,CYLD,CYP4F2,CYP7A1,DAZAP1,DCTN1,DDX60,DEK,DGKZ,DHX9,DIAPH2,DICER1,DIO1,DIPK2A,DIXDC1,DMD,DMTF1,DOCK4,DPP4,DPP9,DSP,DSTN,DUSP19,E2F5,EGR1,EIF1AX,ELF1,ERBB3,ESRRA,EXOC6,EXTL2,F11,F2R,F3,FAM102A,FAM160B1,FAM172A,FECH,FGA,FGB,FGFR2,FGG,FH,FLCN,FMO1,FMO5,FOXO3,FTSJ3,G6PD,GAS2L3,GNA12,GNG12,GOLGB1,GPD2,GRHPR,GSK3A,GSS,GSTM2,GTF2F1,GTF3C2,GYG1,H	384

ECA,HECTD3,HERC
4,HMGCR,HNRNPC,
HRAS,HSPB1,ICK,IG
FBP2,IL18,ING4,IRF6
,IRS1,ITPA,JAK2,JMJ
D1C,KAT2B,KCMF1,
KDM6A,KEAP1,KIAA
0100,KITLG,KLF12,K
LHL7,KPNB1,KTN1,K
YNU,L2HGDH,LGR5,
LIN7A,LMNA,LRIG1,L
RP12,LRRC28,LRRC
3,LSM14B,LTBR,MA
GED1,MAGI3,MAP2K
3,MAP3K7,MBNL2,M
COLN1,MEF2A,MIA2,
MKNK2,MLLT10,MLX
,MOCOS,MON2,MPD
Z,MPP1,MPRIP,MRNI
P,MRPL44,MRPS22,
MRPS27,MRPS9,MY
O1B,MYO5B,MYO6,N
4BP2L1,NCOA1,NDU
FA9,NEK7,NET1,NFK
BIA,NGEF,NHLRC2,
NIPBL,NME1,NOSIP,
NPC1,NR1H4,NR2F6
,NR3C2,NRIP1,NUDT
1,NUDT19,NUDT7,N
UP62,NUP88,OAZ1,
OSBPL1A,OXR1,PAL
MD,PAN2,PAN3,PAQ
R9,PCDH1,PCGF5,P
DCD2,PDE2A,PDE9A
,PDGFC,PDGFRB,PD
LIM1,PDP2,PDPK1,P
ER1,PER3,PFDN2,P
GAP1,PHF20L1,PHK
A2,PIK3AP1,PIK3CA,
PKHD1,PLD1,PLXNA
2,PMPCA,PNKD,PNR
C1,POR,PPP1R14B,
PPP1R1B,PPP1R9A,
PPP2R1A,PRPF19,P
RPF39,PRPF6,PSMD
1,PSMD2,PSMD8,PT
BP1,PTBP2,QDPR,R
ABEPK,RABGAP1L,R
AI14,RALGPS2,RAS
SF3,RB1CC1,RBBP5,
RBBP7,RCL1,RHBD
D1,RICTOR,ROCK1,
RPA1,RPIA,RUFY3,S
AA4,SARS1,SBF2,SC
APER,SCARB2,SCYL
1,SDC4,SEC63,SECI
SBP2L,SENP6,SERP
INE2,SERPINH1,SES
N3,SETDB2,SGTB,S
HPRH,SLC1A4,SLC2
0A1,SLC20A2,SLC2A
2,SLC2A9,SLC35B3,

					SLC38A2,SLC39A10,SLC39A8,SLC6A12,SLC9A3R1,SMAD7,SMAP1,SNRNP200,SNTB1,SNX10,SNX9,SOAT2,SOD2,SPARC,SPON2,SPOP,SPTBN1,SPTBN2,STARD7,STIP1,STK24,STK40,STOM,STT3B,STX8,STXBP3,SULF2,SYAP1,SYNE1,SYT1,TBC1D16,TBC1D20,TBC1D24,TBRG4,TCP1,TEP1,TEX2,TIGD2,TIMM44,TK1,TLCD4,TLE1,TMEM126A,TMEM41B,TMEM51,TMEM97,TNFAIP2,TNRC6A,TOP1MT,TPI1,TPR,TPRG1L,TRIB3,TTC1,TUBB,TUBB6,TXNL1,UBAC1,UBE2F,UBTF,UGP2,UGT2B10,ULK1,UNG,UROC1,UTP14A,UTRN,UXS1,VPS13A,WDR18,WDR26,WDR81,WIPI2,WNT5B,ZCCHC24,ZFAND2B,ZKSCAN1,ZNF32	
Lipid Metabolism,Small Molecule Biochemistry,Vitamin And Mineral Metabolism	Synthesis	Synthesis Of Terpenoid	2.90E-10	-1.915	ABCG5,ABCG8,ACAT2,ACLY,ACOX2,ADH4,AKR1D1,ALG13,ATP1A1,BAAT,CES1,CYP39A1,CYP7A1,ELOVL6,ESRRA,G6PD,HMGCR,HSD17B2,HSD3B7,IGFBP2,IL18,JAK2,KITLG,KPNB1,LSS,NPC1,NR1H4,NR5A2,NUS1,OSBPL1A,POR,RAN,SERPINE2,SLC27A2,SLC9A3R1,SOAT2,STARD4	37
Cancer,Gastrointestinal Disease,Hepatic System Disease,Organismal Injury And Abnormalities	Liver Tumor	Liver Tumor	2.32E-06	-1.915	ABCB4,ABCG8,ACLY,ACOX2,ACSS2,ADH4,AHNAK,AKAP11,AKAP9,AKR1D1,ALG13,AMDHD1,ANKRD12,ANXA7,ARGLU1,ARHGAP21,ARHGAP6,ASAP2,ASB13,ATP1A1,ATP1B1,ATXN2L,BAP1,BDH1,BHMT,BHMT2,BID,BUD23,CARS1,CBLB,CCT6A,CD47,CDC14B,CDH1,CDH2,CES1,CHD6,CIB3,CLK2,CLMN,CMTM6,CNOT4,COL27A1,CPEB4,CRIP2,CROT,CXADR,CYP39A1,C	266

YP7A1,CYTH1,DCTN
1,DDC,DHX9,DIAPH2
,DICER1,DIPK2A,DM
D,DOCK4,DPP4,DPP
9,E2F5,EIF1AX,EIF4
EBP1,ENPP1,ERBB3
,ESRRA,EXTL2,F3,F
AM160B1,FBXO9,FG
A,FGB,FGFR2,FGG,F
LCN,FOXK1,FPGS,G
6PD,GABARAP,GAS
2L3,GCLC,GNA12,G
OLGB1,GPD2,GRHP
R,GSDMD,GTF3C2,H
ECA,HLF,HMGCR,H
NRNPC,HOMER2,HR
AS,HSD17B2,ID2,ID4
,IGFBP2,IL18,ING4,I
RS1,JMJD1C,KDM6A
,KEAP1,KIAA0100,KL
HDC2,KPNB1,KTN1,
KYNU,LARS2,LGR5,
LMNA,LTBR,MACRO
H2A1,MAGI3,MAP1L
C3B,MAP2K3,MAP3K
7,MEF2A,MON2,MPD
Z,MPRIP,MRNIP,MR
PL24,MRPS22,MYO6
,NCALD,NCOA1,NET
1,NGEF,NIPBL,NME1
,NPC1,NR1H4,NR2F
6,NR3C2,NR5A2,NRI
P1,NT5C,NUDT1,NU
P62,OSBPL1A,OXR1,
PAN2,PAN3,PAQR9,
PCDH1,PDE2A,PDG
FC,PDGFRB,PDLIM1
,PDPK1,PER1,PEX26
,PGAP1,PHF20L1,PI
K3AP1,PIK3CA,PKH
D1,PLD1,PLXNA2,P
MPCA,PNRC1,POR,
PPM1A,PPOX,PPP1
R12A,PPP1R1B,PPP
1R9A,PPP2R1A,PRP
F6,PSMD1,PSMD11,
PSMD2,PSPC1,PSP
H,PTBP2,RABGAP1L
,RALGPS2,RAN,RAS
A3,RB1CC1,RBBP5,
RBBP7,RCL1,RHBD
D1,RICTOR,RNF167,
ROCK1,RPIA,RUFY3,
SAA4,SARS1,SCAPE
R,SCYL1,SDC4,SEC
63,SECISBP2L,SENP
6,SERPINE2,SESN3,
SFRP5,SGK2,SGTB,
SIRT5,SLC10A1,SLC
1A4,SLC20A2,SLC2A
2,SLC2A5,SLC35B3,
SLC38A2,SLC39A10,

					SLC39A8,SLC6A12,SLK,SNRNP200,SNX9,SOAT2,SOD2,SPARC,SPTBN1,SPTBN2,STK24,STK40,STXBP3,SULF2,SUMF1,SYNE1,TBC1D16,TCIM,TEP1,TEX2,THAP4,TIGD2,TIMM44,TLCD4,TLE1,TMEM219,TMEM41A,TMEM62,TNFAIP8L1,TNRC6A,TOP1MT,TPI1,TPR,TSN,TSPAN33,TTC1,TUBB,TUBGCP2,TXNL1,UBAC1,UBE2F,UBXN1,UBXN6,UCK1,UGP2,UGT2B10,UPP2,UROC1,USP47,UTRN,WIPI2,WNT5B,YWHAH,ZCCHC24	
Cancer,Organismal Injury And Abnormalities	Abdominal Carcinoma	Abdominal Carcinoma	2.84E-24	-1.93	AASDH,ABCB1,ABCB4,ABCG2,ABCG5,ABCG8,ABTB2,ACAD8,ACAT2,ACLY,ACOX2,ACSL3,ACSL4,ACSM1,ACSM5,ACSS2,ACTR6,ADH4,AFG3L2,AGPAT2,AHCY,AHNAK,AHSA1,AKAP11,AKAP9,AKR1D1,ALAS1,ALDH1L1,ALG13,AMDHD1,ANKRD12,ANP32A,ANXA7,ARF3,ARGLU1,ARHGAP18,ARHGAP21,ARHGAP6,ARIH2,ARL15,ARL6IP4,ARSG,ASAP2,ASB13,ATG3,ATG4D,ATP1A1,ATP1B1,ATP6V1D,ATXN2L,AUH,BAAT,BAP1,BDH1,BHMT,BHMT2,BID,BIK,BLMH,BRD8,BUD23,C11orf54,C9orf152,CA3,CAMKK2,CAPN2,CARS1,CBLB,CCBE1,CCDC66,CCT3,CCT4,CCT5,CCT6A,CCT7,CD2AP,CD47,CD9,CD99L2,CDA,CDC14B,CDH1,CDH2,CDO1,CEBPD,CENPV,CES1,CGN,CHCHD3,CHCHD4,CHD6,CHPT1,CHRAC1,CIB3,CITED2,CLDND1,CLK2,CLMN,CLOCK,CMTM6,CMTM8,CNOT4,CNOT6,CNP,COL27A1,COL5A3,CORO1B,CPEB4,CPT1A,CRIP2,CROT,	540

CS,CSRNP1,CTDSP
1,CTPS2,CTSH,CUL4
B,CXADR,CYLD,CYP
2U1,CYP39A1,CYP4
F2,CYP7A1,CYTH1,D
AZAP1,DCTN1,DDC,
DDX60,DEK,DGKZ,D
HX9,DIAPH2,DICER1
,DIO1,DIPK2A,DIXDC
1,DMD,DMTF1,DNAJ
C5,DOCK4,DPP4,DP
P9,DSP,DSTN,DUSP
19,DVL1,E2F5,ECHD
C1,EEPD1,EFHD2,E
GR1,EIF1AX,ELF1,E
NPP1,ERBB3,ESRRA
,EXOC6,EXOSC2,EX
TL2,F11,F2R,F3,FAD
S2,FAM102A,FAM16
0B1,FAM172A,FAM8
9A,FBXO9,FECH,FG
A,FGB,FGFR2,FGFR
L1,FGG,FH,FKBP4,F
LCN,FMO1,FMO5,FO
XK1,FOXO3,FPGS,F
TSJ3,G6PD,GAS2L3,
GCAT,GCLC,GCNT2,
GDA,GNA12,GNG12,
GOLGB1,GPD2,GPN
1,GRHPR,GSDMD,G
SK3A,GSS,GSTM2,G
TF2A2,GTF2F1,GTF3
C2,GYG1,HECA,HEC
TD3,HERC4,HLF,HM
G20B,HMGCR,HNRN
PC,HNRNPD,HOME
R2,HOOK1,HRAS,HS
D17B2,HSD3B7,HSP
B1,ICK,ID2,ID4,IGFB
P2,IL18,ING4,IRF6,IR
S1,ITPA,ITPK1,JAK2,
JMJD1C,KAT2B,KCM
F1,KDM6A,KEAP1,KI
AA0100,KITLG,KLF1
2,KLF9,KLHDC2,KLH
L21,KLHL7,KPNB1,K
TN1,KYNU,L2HGDH,
LARS2,LGR5,LIN7A,
LIN7C,LITAF,LMNA,L
PIN1,LRIG1,LRP12,L
RRC28,LRRC3,LSM1
4B,LSS,LTBR,MACR
OH2A1,MAGED1,MA
GI3,MAP1LC3B,MAP
2K3,MAP3K7,MBNL2,
MCOLN1,MEF2A,MIA
2,MKNK2,MLLT10,ML
X,MMD,MOCOS,MO
N2,MPDZ,MPP1,MPR
IP,MRNIP,MRPL12,M
RPL20,MRPL24,MRP
L44,MRPS22,MRPS2

5,MRPS27,MRPS9,M
YO1B,MYO5B,MYO6,
N4BP2,N4BP2L1,NA
GK,NCALD,NCOA1,N
DUFA12,NDUFA9,NE
K7,NET1,NFKBIA,NG
EF,NHLRC2,NINJ1,NI
PBL,NME1,NOSIP,N
PC1,NR1H4,NR2F6,
NR3C2,NR5A2,NRIP
1,NT5C,NUDT1,NUD
T19,NUDT7,NUP62,N
UP88,OSBPL1A,OTU
D6B,OXR1,PALMD,P
AN2,PAN3,PAQR9,P
CDH1,PCGF5,PDCD
2,PDE2A,PDE9A,PD
GFC,PDGFRB,PDLIM
1,PDP2,PDPK1,PDR
G1,PER1,PER3,PEX
26,PEX6,PFDN2,PGA
P1,PHF20L1,PHKA2,
PIK3AP1,PIK3CA,PK
HD1,PLD1,PLXNA2,P
MPCA,PNKD,PNRC1,
POR,PPIF,PPM1A,P
POX,PPP1R12A,PPP
1R14B,PPP1R1B,PP
P1R3B,PPP1R9A,PP
P2R1A,PRPF39,PRP
F6,PRPS1,PSEN2,PS
MD1,PSMD11,PSMD
2,PSMD8,PSPC1,PS
PH,PTBP1,PTBP2,PX
MP4,QDPR,RAB11FI
P2,RABEPK,RABGA
P1L,RAI14,RALGPS2
,RAN,RASA3,RASSF
3,RB1CC1,RBBP5,R
BBP7,RCAN1,RCL1,
REPIN1,RGS2,RHBD
D1,RHOD,RICTOR,R
NF167,ROCK1,RPA1,
RPIA,RUFY3,SAA4,S
AR1B,SARS1,SBF2,S
CAPER,SCARB2,SC
YL1,SDC4,SDR42E1,
SEC63,SECISBP2L,S
ENP6,SERPINA6,SE
RPINE2,SERPINH1,S
ESN3,SETDB2,SFRP
5,SGK2,SGTB,SH3D
19,SHMT1,SHPRH,SI
RT5,SLC10A1,SLC1A
4,SLC20A1,SLC20A2
,SLC25A32,SLC27A2
,SLC2A2,SLC2A5,SL
C2A9,SLC35B3,SLC3
8A2,SLC39A10,SLC3
9A8,SLC6A12,SLC9A
3R1,SLK,SMAD7,SM
AP1,SNAPC2,SNRN

					P200,SNTB1,SNX10,SNX9,SOAT2,SOD2,SPARC,SPG21,SPOP,SPTBN1,SPTBN2,ST3GAL4,STARD7,STIP1,STK24,STK40,STOM,STT3B,STX8,STXBP3,SULF2,SUMF1,SYAP1,SYNE1,SYT1,TBC1D16,TBC1D20,TBC1D24,TBRG4,TCIM,TCP1,TEP1,TEX2,THAP4,THRSP,TIGD2,TIMM44,TK1,TLE1,TMBIM1,TMEM126A,TMEM219,TMEM41A,TMEM41B,TMEM51,TMEM62,TMEM97,TNFAIP2,TNFAIP8L1,TNRC6A,TOP1MT,TPI1,TPR,TPRG1L,TPRKB,TRIB3,TRMT1,TSN,TSPAN33,TTC1,TTC19,TUBB,TUBB6,TUBGCP2,TXNL1,UBAC1,UBE2F,UBE2L3,UBR1,UBTF,UBXN1,UBXN6,UCK1,UGP2,UGT2B10,ULK1,UNG,UPP2,UROC1,UROD,USP47,UTP14A,UTRN,UXS1,VPS13A,WDR18,WDR26,WDR81,WIPI2,WNT5B,YPEL3,YWHAH,ZCCHC24,ZFAND2B,ZKSCAN1,ZNF23,ZNF32,ZNF326,ZNF654	
Cancer,Gastrointestinal Disease,Organismal Injury And Abnormalities	Digestive Organ Tumor	Digestive Organ Tumor	6.58E-16	-1.933	AASDH,ABCB1,ABCB4,ABCG2,ABCG5,ABCG8,ABTB2,ACAD8,ACAT2,ACLY,ACOX2,ACSL4,ACSL5,ACSM5,ACSS2,ACTR6,ADH4,AFG3L2,AGPAT2,AHCY,AHNAK,AHSA1,AKAP11,AKAP9,AKR1D1,ALAS1,ALDH1L1,ALG13,AMDHD1,ANKRD12,ANP32A,ANXA7,ARF3,ARGLU1,ARHGAP18,ARHGAP21,ARHGAP6,ARIH2,ARL6IP4,ARSG,ASAP2,ASB13,ATG3,ATG4D,ATP1A1,ATP1B1,ATP6V1D,ATXN2L,BAAT,BAP1,BDH1,BHMT,BHMT2,BID,BLMH,BRD8,BUD23,C11orf54,C9orf152,C9orf16,CAMKK2,CAPN2,	517

CARS1,CBLB,CCBE1
,CCDC66,CCT4,CCT
5,CCT6A,CCT7,CD2
AP,CD47,CD9,CD99L
2,CDC14B,CDH1,CD
H2,CDO1,CEBPD,CE
NPV,CES1,CGN,CHC
HD3,CHCHD4,CHD6,
CHPT1,CHRAC1,CIB
3,CITED2,CLDND1,C
LK2,CLMN,CLOCK,C
MTM6,CNOT4,CNP,C
OL27A1,COL5A3,CO
RO1B,CPEB4,CPT1A
,CRIP2,CROT,CS,CT
DSP1,CTPS2,CTSH,
CUL4B,CXADR,CYL
D,CYP2U1,CYP39A1,
CYP4F2,CYP7A1,CY
TH1,DAZAP1,DCTN1
,DDC,DDX60,DEK,D
GKZ,DHX9,DIAPH2,D
ICER1,DIO1,DIPK2A,
DIXDC1,DMD,DMTF1
,DNAJC5,DOCK4,DP
P4,DPP9,DSP,DSTN,
DUSP19,DVL1,E2F5,
ECHDC1,ECHDC3,E
EPD1,EGR1,EIF1AX,
EIF4EBP1,ELF1,ENP
P1,ERBB3,ESRRA,E
XOC6,EXOSC2,EXTL
2,F11,F2R,F3,FADS2
,FAM102A,FAM160B
1,FAM172A,FBXO9,F
ECH,FGA,FGB,FGFR
2,FGFRL1,FGG,FKB
P4,FLCN,FMO1,FMO
5,FOXK1,FOXO3,FP
GS,FTSJ3,G6PD,GA
BARAP,GAS2L3,GCA
T,GCLC,GCNT2,GDA
,GNA12,GNG12,GOL
GB1,GPD2,GPN1,GR
HPR,GSDMD,GSK3A
,GSS,GTF2F1,GTF3
C2,HECA,HECTD3,H
ERC4,HLF,HMG20B,
HMGCR,HNRNPC,H
NRNPD,HOMER2,HO
OK1,HRAS,HSD17B2
,HSD3B7,HSPB1,ICK
,ID2,ID4,IGFBP2,IL18
,ING4,IRF6,IRS1,ITP
K1,JAK2,JMJD1C,KA
T2B,KCMF1,KDM6A,
KEAP1,KIAA0100,KIT
LG,KLF12,KLF9,KLH
DC2,KLHL21,KLHL7,
KPNB1,KTN1,KYNU,
LARS2,LGALS4,LGR
5,LIN7A,LIN7C,LMNA

,LRIG1,LRP12,LRRC
28,LRRC3,LSM14B,L
SS,LTBR,MACROH2
A1,MAGED1,MAGI3,
MAP1LC3B,MAP2K3,
MAP3K7,MBNL2,MC
OLN1,MEF2A,MIA2,
MKNK2,MLLT10,MLX
,MOCOS,MON2,MPD
Z,MPRIP,MRNIP,MR
PL12,MRPL20,MRPL
24,MRPS22,MRPS25
,MRPS27,MRPS9,MT
1F,MYO1B,MYO5B,M
YO6,N4BP2,NAGK,N
CALD,NCOA1,NDUF
A12,NDUFA9,NEK7,
NET1,NFKBIA,NGEF,
NHLRC2,NINJ1,NIPB
L,NME1,NOSIP,NPC
1,NR1H4,NR2F6,NR3
C2,NR5A2,NRIP1,NT
5C,NUDT1,NUDT19,
NUDT7,NUP62,NUP8
8,OAZ1,OSBPL1A,O
XR1,PALMD,PAN2,P
AN3,PAQR9,PCDH1,
PCGF5,PDE2A,PDE9
A,PDGFC,PDGFRB,P
DLIM1,PDP2,PDPK1,
PDRG1,PER1,PER3,
PES1,PEX26,PEX6,P
FDN2,PGAP1,PHF20
L1,PHKA2,PIK3AP1,
PIK3CA,PKHD1,PLD
1,PLXNA2,PMPCA,P
NKD,PNRC1,POR,PP
M1A,PPOX,PPP1R12
A,PPP1R14B,PPP1R
1B,PPP1R3B,PPP1R
9A,PPP2R1A,PRDX6,
PRPF39,PRPF6,PRP
S1,PSMD1,PSMD11,
PSMD2,PSMD8,PSM
F1,PSPC1,PSPH,PT
BP1,PTBP2,PXMP4,
QDPR,RAB11FIP2,R
ABGAP1L,RAI14,RAL
GPS2,RAN,RASA3,R
ASSF3,RB1CC1,RBB
P5,RBBP7,RCL1,RE
PIN1,RGS2,RHBDD1,
RHOD,RICTOR,RNF
167,ROCK1,RPA1,R
PIA,RUFY3,SAA4,SA
R1B,SARS1,SBF2,S
CAPER,SCARB2,SC
YL1,SDC4,SEC63,SE
CISBP2L,SENP6,SE
RPINA6,SERPINE2,S
ERPINH1,SESN3,SE
TDB2,SFRP5,SGK2,

					SGTB,SH3D19,SHMT1,SHPRH,SIRT5,SLC10A1,SLC1A4,SLC20A1,SLC20A2,SLC27A2,SLC2A2,SLC2A5,SLC2A9,SLC30A10,SLC35B1,SLC35B3,SLC38A2,SLC39A10,SLC39A8,SLC6A12,SLC9A3R1,SLK,SMAD7,SMAP1,SNAPC2,SNRNP200,SNTB1,SNX10,SNX9,SOAT2,SOD2,SPARC,SPG21,SPON2,SPOP,SPTBN1,SPTBN2,ST3GAL4,STARD7,STIP1,STK24,STK40,STOM,STT3B,STX8,STXBP3,SULF2,SUMF1,SYAP1,SYNE1,SYT1,TBC1D16,TBC1D20,TBC1D24,TBRG4,TCIM,TCP1,TEP1,TEX2,THAP4,THRSP,TIGD2,TIMM44,TK1,TLCD4,TLE1,TMBIM1,TMEM126A,TMEM219,TMEM41A,TMEM41B,TMEM62,TMEM97,TNFAIP8L1,TNRC6A,TOP1MT,TPI1,TPR,TPRKB,TRIB3,TRMT1,TSN,TSPAN33,TTC1,TTC19,TUBB,TUBB6,TUBGCP2,TXNL1,UBAC1,UBE2F,UBE2L3,UBR1,UBTF,UBXN1,UBXN6,UCK1,UGP2,UGT2B10,ULK1,UNG,UPP2,UROC1,UROD,USP47,UTP14A,UTRN,UXS1,VPS13A,WDR18,WDR26,WDR81,WIPI2,WNT5B,YWHAH,ZCCHC24,ZFAND2B,ZNF23,ZNF32,ZNF326,ZNF654	
Dermatological Diseases And Conditions,Organismal Injury And Abnormalities	Keratosis	Keratosis	2.57E-04	-1.939	CDH1,CYP7A1,DSP,ENPP1,FGFR2,G6PD,HRAS,ITPA,KEAP1,LRIG1,NFKBIA,NR1H4,PIK3CA,PSEN2	14
Cancer,Gastrointestinal Disease,Organismal Injury And Abnormalities	Colon Cancer	Colon Cancer	1.83E-05	-1.982	AASDH,ABCB1,ABCG5,ACLY,ACTR6,AFG3L2,AGPAT2,AHCY,AKAP9,ALDH1L1,ALG13,ARHGAP21,ARL6IP4,ASAP2,ASB13,ATG3,ATG4D,ATXN2L,BAAT,BAP1,C11orf54,C9orf152,CARS1,CC	228

DC66,CCT4,CCT5,C
CT7,CD2AP,CD99L2,
CDH1,CDH2,CDO1,C
ES1,CGN,CHCHD3,C
HD6,CHPT1,CHRAC
1,CLDND1,CLK2,CL
MN,CLOCK,CMTM6,
CNOT4,CNP,COL27A
1,COL5A3,CPEB4,CP
T1A,CS,CTDSP1,CT
PS2,CUL4B,CYLD,C
YP4F2,DCTN1,DDX6
0,DHX9,DICER1,DM
D,DMTF1,DOCK4,DS
TN,EGR1,ELF1,ERB
B3,ESRRA,F3,FAM16
0B1,FGA,FGB,FGFR
2,FLCN,FMO1,FMO5,
G6PD,GAS2L3,GNA1
2,GNG12,GSS,HECA
,HECTD3,HMGCR,H
NRNPC,HSD17B2,H
SPB1,ICK,IGFBP2,IL
18,IRF6,IRS1,JAK2,J
MJD1C,KDM6A,KEA
P1,KIAA0100,KITLG,
KLF12,KLHL7,KPNB1
,KTN1,LGR5,LIN7A,L
RIG1,LRP12,LRRC3,
MACROH2A1,MAGE
D1,MAP3K7,MIA2,MK
NK2,MLLT10,MLX,M
ON2,MPRIP,MRNIP,
MRPS9,MT1F,MYO5
B,MYO6,NCOA1,NEK
7,NET1,NGEF,NHLR
C2,NIPBL,NOSIP,NR
3C2,NUDT19,NUDT7,
NUP62,OSBPL1A,PA
QR9,PCDH1,PCGF5,
PDE9A,PDGFRB,PD
P2,PDPK1,PER1,PF
DN2,PGAP1,PHF20L
1,PHKA2,PIK3AP1,PI
K3CA,PKHD1,PLD1,
PLXNA2,PNKD,PNR
C1,PPP1R14B,PPP1
R1B,PPP2R1A,PRPF
39,PRPF6,PSMD8,PT
BP1,QDPR,RABGAP
1L,RASSF3,RB1CC1,
RBBP5,RICTOR,ROC
K1,RPA1,RUFY3,SA
A4,SBF2,SCAPER,S
CYL1,SEC63,SECISB
P2L,SENP6,SESN3,S
GK2,SHPRH,SLC1A4
,SLC20A1,SLC20A2,
SLC27A2,SLC2A2,SL
C35B3,SLC38A2,SLC
39A10,SLC39A8,SLC
6A12,SMAD7,SMAP1

					,SNRNP200,SNX9,SOAT2,SPOP,SPTBN1,SPTBN2,STIP1,STX8,SULF2,SYAP1,SYNE1,SYT1,TBC1D16,TBC1D24,TBRG4,TCP1,TEP1,TIMM44,TK1,TMEM126A,TMEM41B,TMEM97,TNRC6A,TOP1MT,TPR,TTC1,TUBB,UBAC1,UBTF,ULK1,UNG,UROC1,UTRN,VPS13A,WDR18,WDR26,WDR81,WNT5B,ZCCHC24	
Cancer,Organismal Injury And Abnormalities	Anogenital Cancer	Anogenital Cancer	9.09E-10	-1.996	ABCB1,ABCB4,ABCG2,ABCG5,ACAD8,ACLY,ACOX2,ACSL3,ACSL4,ACSM1,ACSS2,AGPAT2,AHNAK,AKAP11,AKAP9,ALAS1,ALDH1L1,ALG13,ANKRD12,ARHGAP18,ARHGAP21,ARHGAP6,ARIH2,ARL15,ASAP2,ATP1A1,ATP6V1D,ATXN2L,AUH,BAAT,BAP1,BDH1,BIK,BLMH,BRD8,CA3,CAPN2,CBLB,CCT3,CCT5,CCT6A,CD2AP,CD47,CD9,CD99L2,CDA,CDH1,CDH2,CDO1,CEBPD,CES1,CGN,CHD6,CLDND1,CLK2,CLMN,CMTM8,CNOT4,CNOT6,CNP,COL27A1,COL5A3,CPEB4,CPT1A,CS,CSRNP1,CTSH,CXADR,CYLD,CYP2U1,CYP4F2,CYP7A1,CYTH1,DAZAP1,DCTN1,DDX60,DGKZ,DHX9,DIAPH2,DICER1,DIO1,DIPK2A,DIXDC1,DMD,DOCK4,DPP4,DPP9,DSP,DSTN,E2F5,EFHD2,EGR1,EIF1AX,EIF4EBP1,ELF1,ELOVL6,ENPP1,ERBB3,ESRRA,EXOC6,EXOSC2,F11,F2R,F3,FADS2,FAM160B1,FAM172A,FAM89A,FECH,FGA,FGFR2,FGFRL1,FGG,FH,FKBP4,FLCN,FOXK1,FOXO3,FTSJ3,GDA,GNA12,GOLGB1,GPD2,GRHPR,GSDMD,GSK3A,GSTM2,GTF2A2,GYG1,HECA,HECTD	358

3,HLF,HMGCR,HRAS
,HSD3B7,HSPB1,ICK
,ID2,IGFBP2,IL18,IN
G4,IRF6,IRS1,ITPA,J
AK2,JMJD1C,KAT2B,
KCMF1,KDM6A,KEA
P1,KIAA0100,KLF9,K
TN1,L2HGDH,LARS2
,LGALS4,LGR5,LIN7
C,LITAF,LMNA,LRIG
1,LRP12,LRRC28,LS
M14B,LSS,MACROH
2A1,MAGED1,MAGI3
,MAP1LC3B,MAP2K3
,MBNL2,MCOLN1,ME
F2A,MIA2,MKNK2,ML
LT10,MMD,MOCOS,
MON2,MPDZ,MPRIP,
MRPL24,MRPL34,MR
PL44,MRPS22,MRPS
25,MRPS27,MT1F,M
YO1B,MYO5B,MYO6,
N4BP2,NCOA1,NDU
FA9,NDUFB9,NEK7,
NFKBIA,NHLRC2,NIP
BL,NME1,NOSIP,NP
C1,NR2F6,NR3C2,N
R5A2,NRIP1,NUDT7,
NUP62,NUP88,NUS1
,OAZ1,OSBPL1A,OT
UD6B,OXR1,PALMD,
PAN3,PCDH1,PCGF5
,PDCD2,PDE2A,PDG
FRB,PDLIM1,PDP2,P
DPK1,PER1,PER3,P
EX6,PGAP1,PHF20L
1,PHKA2,PIK3CA,PK
HD1,PLD1,PLXNA2,P
NRC1,POR,PPIF,PP
P1R1B,PPP1R3B,PP
P1R9A,PPP2R1A,PR
PF39,PRPF6,PSEN2,
PSMD1,PSMD2,PTB
P1,PTBP2,PXMP4,R
AB11FIP2,RABEPK,R
ABGAP1L,RALGPS2,
RASA3,RB1CC1,RBB
P5,RBBP7,RCAN1,R
CL1,REPIN1,RGS2,R
HOD,RICTOR,RNF14
,RNF144A,ROCK1,R
PIA,SBF2,SCAPER,S
CARB2,SCYL1,SDC4
,SDR42E1,SECISBP2
L,SERPINA6,SERPIN
E2,SESN3,SETDB2,S
FRP5,SIRT5,SLC16A
5,SLC1A4,SLC20A1,
SLC20A2,SLC25A32,
SLC2A2,SLC38A2,SL
C39A10,SLC6A12,SL
C9A3R1,SLK,SMAP1,

					SNRNP200,SNTB1,SNX10,SOD2,SPARC,SPG21,SPOP,SPTBN1,SPTBN2,ST3GAL4,STARD7,STK24,STK40,STOM,STT3B,STX8,STXBP3,SULF2,SYNE1,TBC1D16,TBC1D20,TBC1D24,TCP1,TEP1,TEX2,THRSP,TIFA,TIGD2,TIMM44,TK1,TLE1,TMEM126A,TMEM97,TNFAIP2,TNRC6A,TOP1MT,TPI1,TPR,TPRG1L,TRIB3,TRMT1,TSPAN33,TTC1,TUBB,TUBB6,TXNL1,UBAC1,UBE2F,UBR1,UGP2,UGT2B10,UROC1,UROD,USP47,UTRN,UXS1,VPS13A,WNT5B,YPEL3,YWHAH,ZCCHC24,ZFAND2B,ZKSCAN1,ZNF23,ZNF32,ZNF654	
Cancer,Organismal Injury And Abnormalities	Genitourinary Carcinoma	Genitourinary Carcinoma	9.18E-11	-2	ABCB1,ABCB4,ABCG2,ABCG5,ACAD8,ACLY,ACOX2,ACSL3,ACSL4,ACSM1,ACSS2,AFG3L2,AGPAT2,AHNAK,AKAP11,AKAP9,ALAS1,ALDH1L1,ALG13,ANKRD12,ARHGAP18,ARHGAP21,ARHGAP6,ARIH2,ARL15,ASAP2,ATP1A1,ATP6V1D,ATXN2L,AUH,BAAT,BAP1,BDH1,BIK,BRD8,CA3,CAPN2,CARS1,CBLB,CCT3,CCT5,CCT6A,CD2AP,CD47,CD9,CDA,CDC14B,CDH1,CDH2,CDO1,CES1,CGN,CHD6,CLDND1,CLK2,CLMN,CMTM8,CNOT4,CNOT6,CNP,COL5A3,CPEB4,CPT1A,CROT,CS,CSRNP1,CTSH,CXADR,CYP4F2,CYP7A1,CYTH1,DAZAP1,DCTN1,DDC,DDX60,DGKZ,DHX9,DIAPH2,DICER1,DIO1,DIPK2A,DMD,DOCK4,DPP4,DPP9,DSP,DSTN,DUSP19,EFHD2,EIF1AX,ELF1,ENPP1,ERBB3,ESRRA,EXOC6,EXTL2,F11,F2R,F3,FAM160B1,FAM172A,FAM89A,FECH,FGA,FG	346

FR2,FGFRL1,FGG,F
H,FLCN,FOXK1,FOX
O3,FTSJ3,G6PD,GD
A,GNA12,GOLGB1,G
PD2,GRHPR,GSDMD
,GSK3A,GSTM2,GTF
2A2,GTF3C2,GYG1,
HECA,HECTD3,HLF,
HMGCR,HRAS,HSD3
B7,ICK,ID2,IGFBP2,I
L18,ING4,IRF6,IRS1,I
TPA,JAK2,JMJD1C,K
AT2B,KCMF1,KDM6A
,KEAP1,KIAA0100,KL
HDC2,KLHL7,KTN1,K
YNU,L2HGDH,LARS2
,LGR5,LIN7C,LITAF,L
MNA,LPIN1,LRIG1,L
RP12,LRRC28,LSM1
4B,LSS,MAGED1,MA
GI3,MAP1LC3B,MAP
2K3,MBNL2,MCOLN1
,MEF2A,MIA2,MLLT1
0,MMD,MOCOS,MON
2,MPDZ,MPP1,MPRI
P,MRPL44,MRPS22,
MRPS25,MRPS27,M
T1F,MYO1B,MYO5B,
MYO6,N4BP2,N4BP2
L1,NAGK,NCOA1,ND
UFA9,NEK7,NFKBIA,
NGEF,NHLRC2,NIPB
L,NOSIP,NPC1,NR2F
6,NR5A2,NRIP1,NUD
T7,NUP62,NUP88,OS
BPL1A,OTUD6B,OXR
1,PALMD,PAN3,PCD
H1,PCGF5,PDCD2,P
DE2A,PDGFRB,PDLI
M1,PDP2,PDPK1,PD
RG1,PER1,PER3,PE
X6,PGAP1,PHF20L1,
PHKA2,PIK3CA,PKH
D1,PLD1,PLXNA2,PN
KD,PNRC1,POR,PPI
F,PPP1R1B,PPP1R3
B,PPP1R9A,PPP2R1
A,PRPF6,PSEN2,PS
MD1,PSMD2,PTBP1,
PTBP2,PXMP4,RAB1
1FIP2,RABEPK,RAB
GAP1L,RAI14,RALG
PS2,RB1CC1,RBBP5
,RBBP7,RCAN1,RCL
1,REPIN1,RGS2,RH
OD,RICTOR,ROCK1,
RPIA,SBF2,SCAPER,
SCARB2,SCYL1,SDC
4,SDR42E1,SEC63,S
ECISBP2L,SENP6,S
ERPINA6,SERPINE2,
SESN3,SETDB2,SHP

					RH,SLC1A4,SLC20A1,SLC20A2,SLC25A32,SLC2A2,SLC2A9,SLC38A2,SLC39A10,SLC39A8,SLC6A12,SLC9A3R1,SLK,SMAD7,SMAP1,SNRNP200,SNTB1,SNX10,SOD2,SPARC,SPOP,SPTBN1,SPTBN2,ST3GAL4,STARD7,STK24,STK40,STOM,STT3B,STX8,STXBP3,SULF2,SYNE1,TBC1D16,TBC1D20,TBC1D24,TEP1,TEX2,TIFA,TIGD2,TK1,TLE1,TMEM126A,TMEM51,TNFAIP2,TNRC6A,TOP1MT,TPI1,TPR,TPRG1L,TRIB3,TRMT1,TSPAN33,TTC1,TUBB,TUBB6,TXNL1,UBAC1,UBE2F,UBR1,UGP2,UGT2B10,UROC1,USP47,UTP14A,UTRN,UXS1,VPS13A,WIPI2,YPEL3,ZCCHC24,ZFAND2B,ZKSCAN1,ZNF23,ZNF32,ZNF654	
Lipid Metabolism,Molecular Transport,Small Molecule Biochemistry	Transport	Transport Of Oleic Acid	8.02E-06	-2	ACSL3,ACSL4,ACSL5,NPC1	4
Lipid Metabolism,Molecular Transport,Small Molecule Biochemistry	Transport	Transport Of Long Chain Fatty Acid	1.05E-03	-2	ACSL3,ACSL4,ACSL5,CROT,NPC1	5
Cancer,Organismal Injury And Abnormalities	Malignant Solid Tumor	Malignant Solid Tumor	8.12E-30	-2.004	AASDH,ABCB1,ABCB4,ABCG2,ABCG5,ABCG8,ABTB2,ACAD8,ACAT2,ACLY,ACOX2,ACSL3,ACSL4,ACSL5,ACSM1,ACSM5,ACSS2,ACTR6,ADH4,AFG3L2,AGPAT2,AHCY,AHNAK,AHSA1,AKAP11,AKAP9,AKR1D1,ALAS1,ALDH1L1,ALG13,AMDHD1,ANKRD12,ANP32A,ANXA7,ARF3,ARGLU1,ARHGAP18,ARHGAP21,ARHGAP6,ARIH2,ARL15,ARL4A,ARL6IP4,ARSG,ASAP2,ASB13,ATG3,ATG4D,ATP1A1,ATP1B1,ATP6V1D,ATXN2L,AUH,BAAT,BAP1,BDH1,BHMT,	597

BHMT2,BID,BIK,BLM
H,BRD8,BUD23,C11o
rf54,C9orf152,C9orf1
6,CA3,CAMKK2,CAP
N2,CARS1,CBLB,CC
BE1,CCDC66,CCT3,
CCT4,CCT5,CCT6A,
CCT7,CD2AP,CD47,
CD9,CD99L2,CDA,C
DC14B,CDH1,CDH2,
CDO1,CEBPD,CENP
V,CES1,CGN,CHCH
D3,CHCHD4,CHD6,C
HPT1,CHRAC1,CIB3,
CITED2,CLDND1,CL
K2,CLMN,CLOCK,CL
TA,CMTM6,CMTM8,
CNN3,CNOT4,CNOT
6,CNP,COL27A1,CO
L5A3,CORO1B,CPEB
4,CPT1A,CRIP2,CRL
S1,CROT,CS,CSRNP
1,CTDSP1,CTPS2,CT
SH,CUL4B,CXADR,C
YB5B,CYLD,CYP2U1
,CYP39A1,CYP4F2,C
YP7A1,CYTH1,DAZA
P1,DCTN1,DDC,DDX
60,DEK,DGKZ,DHX9,
DIAPH2,DICER1,DIO
1,DIPK2A,DIXDC1,D
MD,DMTF1,DNAJB1,
DNAJC5,DOCK4,DP
P4,DPP9,DSP,DSTN,
DTYMK,DUSP19,DVL
1,E2F5,ECHDC1,EC
HDC3,EEPD1,EFHD2
,EGR1,EIF1AX,EIF4E
BP1,ELF1,ELOVL6,E
NDOG,ENPP1,ERBB
3,ESRRA,EXOC6,EX
OSC2,EXTL2,F11,F2
R,F3,FADS2,FAM102
A,FAM118B,FAM160
B1,FAM172A,FAM76
B,FAM89A,FBXO31,F
BXO9,FECH,FGA,FG
B,FGFR2,FGFRL1,F
GG,FH,FKBP4,FLCN,
FMO1,FMO5,FOXK1,
FOXO3,FPGS,FTSJ3,
G6PD,GABARAP,GA
S2L3,GCAT,GCLC,G
CNT2,GDA,GNA12,G
NG12,GOLGB1,GPD
2,GPN1,GRHPR,GSD
MD,GSK3A,GSS,GST
M2,GTF2A2,GTF2F1,
GTF3C2,GYG1,HEC
A,HECTD3,HERC4,H
LF,HMG20B,HMGCR,
HNRNPC,HNRNPD,H

OMER2,HOOK1,HRA
S,HSD17B2,HSD3B7,
HSPB1,ICK,ID2,ID4,I
GFBP2,IL18,ING4,IR
F6,IRS1,ITPA,ITPK1,
JAK2,JMJD1C,KAT2
B,KCMF1,KDM6A,KE
AP1,KHK,KIAA0100,
KITLG,KLF12,KLF9,K
LHDC2,KLHL21,KLH
L7,KPNB1,KTN1,KYN
U,L2HGDH,LARS2,L
GALS4,LGR5,LIN7A,
LIN7C,LITAF,LMNA,L
PIN1,LRIG1,LRP12,L
RRC28,LRRC3,LSM1
4B,LSS,LTBR,MACR
OH2A1,MAGED1,MA
GI3,MAP1LC3B,MAP
2K3,MAP3K7,MBNL2,
MCOLN1,MEF2A,MIA
2,MKNK2,MLLT10,ML
X,MMD,MOCOS,MO
N2,MPDZ,MPP1,MPR
IP,MRNIP,MRPL12,M
RPL20,MRPL24,MRP
L34,MRPL44,MRPS2
2,MRPS25,MRPS27,
MRPS9,MT1F,MYO1
B,MYO5B,MYO6,N4B
P2,N4BP2L1,NAGK,N
CALD,NCOA1,NDUF
A12,NDUFA9,NDUFB
9,NEK7,NET1,NFKBI
A,NGEF,NHLRC2,NI
CN1,NINJ1,NIPBL,N
ME1,NOP10,NOSIP,
NPC1,NR1H4,NR2F6
,NR3C2,NR5A2,NRIP
1,NT5C,NUDT1,NUD
T19,NUDT7,NUP62,N
UP88,NUS1,OAZ1,O
SBPL1A,OTUD6B,OX
R1,PALMD,PAN2,PA
N3,PAQR9,PCDH1,P
CGF5,PDCD2,PDE2A
,PDE9A,PDGFC,PDG
FRB,PDLIM1,PDP2,P
DPK1,PDRG1,PER1,
PER3,PES1,PEX26,P
EX6,PFDN2,PFKFB1,
PGAP1,PHF20L1,PH
KA2,PIK3AP1,PIK3C
A,PKHD1,PLD1,PLX
NA2,PMPCA,PNKD,P
NRC1,POLE4,POR,P
PIF,PPM1A,PPOX,PP
P1R12A,PPP1R14B,
PPP1R1B,PPP1R3B,
PPP1R9A,PPP2R1A,
PRDX6,PRPF19,PRP
F39,PRPF6,PRPS1,P

RPS2,PSEN2,PSMA6
,PSMD1,PSMD11,PS
MD2,PSMD8,PSME2,
PSMF1,PSMG1,PSP
C1,PSPH,PTBP1,PT
BP2,PXMP4,QDPR,R
AB11FIP2,RAB1B,RA
BEPK,RABGAP1L,RA
I14,RALGPS2,RAN,R
ASA3,RASSF3,RB1C
C1,RBBP5,RBBP7,R
BKS,RCAN1,RCL1,R
EPIN1,RGS2,RHBDD
1,RHOD,RICTOR,RN
F14,RNF144A,RNF16
7,ROCK1,RPA1,RPIA
,RUFY3,SAA4,SAR1
B,SARS1,SBF2,SCA
PER,SCARB2,SCGN,
SCOC,SCYL1,SDC4,
SDR42E1,SEC63,SE
CISBP2L,SENP6,SE
RPINA6,SERPINE2,S
ERPINH1,SESN3,SE
TDB2,SFRP5,SGK2,
SGTB,SH3BGRL2,SH
3D19,SHMT1,SHPRH
,SIRT5,SLC10A1,SLC
16A5,SLC1A4,SLC20
A1,SLC20A2,SLC25A
32,SLC27A2,SLC2A2
,SLC2A5,SLC2A9,SL
C30A10,SLC35A3,SL
C35B1,SLC35B3,SLC
38A2,SLC39A10,SLC
39A8,SLC6A12,SLC9
A3R1,SLK,SMAD7,S
MAP1,SNAPC2,SNR
NP200,SNTB1,SNX1
0,SNX5,SNX9,SOAT2
,SOD2,SPARC,SPG2
1,SPON2,SPOP,SPT
BN1,SPTBN2,ST3GA
L4,STARD4,STARD7,
STIP1,STK24,STK40,
STOM,STT3B,STX18,
STX8,STXBP3,SULF
2,SUMF1,SYAP1,SY
NE1,SYT1,TBC1D16,
TBC1D20,TBC1D24,
TBRG4,TCIM,TCP1,T
EP1,TEX2,THAP4,TH
RSP,TIFA,TIGD2,TIM
M10,TIMM44,TK1,TL
CD4,TLE1,TMBIM1,T
MEM126A,TMEM219,
TMEM41A,TMEM41B
,TMEM51,TMEM62,T
MEM97,TNFAIP2,TN
FAIP8L1,TNRC6A,TO
P1MT,TPI1,TPR,TPR
G1L,TPRKB,TRIB3,T

					RMT1,TSN,TSPAN33,TSTA3,TTC1,TTC19,TUBB,TUBB6,TUBGCP2,TUT4,TXN2,TXNL1,UBAC1,UBE2F,UBE2L3,UBR1,UBTF,UBXN1,UBXN6,UCK1,UGP2,UGT2B10,ULK1,UNG,UPP2,UROC1,UROD,USP47,UTP14A,UTRN,UXS1,VPS13A,WDR18,WDR26,WDR81,WIPI2,WNT5B,YPEL3,YWHAH,ZCCHC24,ZFAND2B,ZKSCAN1,ZNF23,ZNF32,ZNF326,ZNF654	
Cancer,Gastrointestinal Disease,Organismal Injury And Abnormalities	Digestive System Cancer	Digestive System Cancer	8.01E-15	-2.009	AASDH,ABCB1,ABCB4,ABCG2,ABCG5,ABCG8,ABTB2,ACAD8,ACAT2,ACLY,ACOX2,ACSL4,ACSL5,ACSM5,ACSS2,ACTR6,ADH4,AFG3L2,AGPAT2,AHCY,AHNAK,AHSA1,AKAP11,AKAP9,AKR1D1,ALAS1,ALDH1L1,ALG13,AMDHD1,ANKRD12,ANP32A,ANXA7,ARF3,ARGLU1,ARHGAP18,ARHGAP21,ARHGAP6,ARIH2,ARL6IP4,ARSG,ASAP2,ASB13,ATG3,ATG4D,ATP1A1,ATP1B1,ATP6V1D,ATXN2L,BAAT,BAP1,BDH1,BHMT,BHMT2,BID,BLMH,BRD8,BUD23,C1orf54,C9orf152,CAMKK2,CAPN2,CARS1,CBLB,CCBE1,CCDC66,CCT4,CCT5,CCT6A,CCT7,CD2AP,CD47,CD9,CD99L2,CDH1,CDH2,CDO1,CEBPD,CENPV,CES1,CGN,CHCHD3,CHCHD4,CHD6,CHPT1,CHRAC1,CIB3,CITED2,CLDND1,CLK2,CLMN,CLOCK,CMTM6,CNOT4,CNP,COL27A1,COL5A3,CORO1B,CPEB4,CPT1A,CRIP2,CROT,CS,CTDSP1,CTPS2,CTSH,CUL4B,CXADR,CYLD,CYP2U1,CYP39A1,CYP4F2,CYP7A1,CYTH1,DAZAP1,DCTN1,DDC,DDX60,DEK,DGKZ,DHX9,DIA	506

PH2,DICER1,DIO1,DI
PK2A,DIXDC1,DMD,
DMTF1,DNAJC5,DO
CK4,DPP4,DPP9,DS
P,DSTN,DUSP19,DV
L1,E2F5,ECHDC1,EE
PD1,EGR1,EIF1AX,EI
F4EBP1,ELF1,ENPP
1,ERBB3,ESRRA,EX
OC6,EXOSC2,EXTL2
,F11,F2R,F3,FADS2,
FAM102A,FAM160B1
,FAM172A,FBXO9,FE
CH,FGA,FGB,FGFR2
,FGFRL1,FGG,FKBP
4,FLCN,FMO1,FMO5,
FOXK1,FOXO3,FPG
S,FTSJ3,G6PD,GAS2
L3,GCAT,GCLC,GCN
T2,GDA,GNA12,GNG
12,GOLGB1,GPD2,G
PN1,GRHPR,GSDMD
,GSK3A,GSS,GTF2F
1,GTF3C2,HECA,HE
CTD3,HERC4,HLF,H
MG20B,HMGCR,HNR
NPC,HNRNPD,HOM
ER2,HOOK1,HRAS,H
SD17B2,HSD3B7,HS
PB1,ICK,ID2,ID4,IGF
BP2,IL18,ING4,IRF6,I
RS1,ITPK1,JAK2,JMJ
D1C,KAT2B,KCMF1,
KDM6A,KEAP1,KIAA
0100,KITLG,KLF12,K
LF9,KLHDC2,KLHL21
,KLHL7,KPNB1,KTN1
,KYNU,LARS2,LGAL
S4,LGR5,LIN7A,LIN7
C,LMNA,LRIG1,LRP1
2,LRRC28,LRRC3,LS
M14B,LSS,LTBR,MA
CROH2A1,MAGED1,
MAGI3,MAP1LC3B,M
AP2K3,MAP3K7,MBN
L2,MCOLN1,MEF2A,
MIA2,MKNK2,MLLT1
0,MLX,MOCOS,MON
2,MPDZ,MPRIP,MRN
IP,MRPL12,MRPL20,
MRPL24,MRPS22,M
RPS25,MRPS27,MR
PS9,MT1F,MYO1B,M
YO5B,MYO6,N4BP2,
NAGK,NCALD,NCOA
1,NDUFA12,NDUFA9
,NEK7,NET1,NFKBIA,
NGEF,NHLRC2,NINJ
1,NIPBL,NME1,NOSI
P,NPC1,NR1H4,NR2
F6,NR3C2,NR5A2,N
RIP1,NT5C,NUDT1,N

UDT19,NUDT7,NUP6
2,NUP88,OAZ1,OSB
PL1A,OXR1,PALMD,
PAN2,PAN3,PAQR9,
PCDH1,PCGF5,PDE2
A,PDE9A,PDGFC,PD
GFRB,PDLIM1,PDP2,
PDPK1,PDRG1,PER1
,PER3,PES1,PEX26,
PEX6,PFDN2,PGAP1
,PHF20L1,PHKA2,PI
K3AP1,PIK3CA,PKH
D1,PLD1,PLXNA2,P
MPCA,PNKD,PNRC1,
POR,PPM1A,PPOX,P
PP1R12A,PPP1R14B
,PPP1R1B,PPP1R3B,
PPP1R9A,PPP2R1A,
PRPF39,PRPF6,PRP
S1,PSMD1,PSMD11,
PSMD2,PSMD8,PSM
F1,PSPC1,PSPH,PT
BP1,PTBP2,PXMP4,
QDPR,RAB11FIP2,R
ABGAP1L,RAI14,RAL
GPS2,RAN,RASA3,R
ASSF3,RB1CC1,RBB
P5,RBBP7,RCL1,RE
PIN1,RGS2,RHBDD1,
RICTOR,RNF167,RO
CK1,RPA1,RUFY3,S
AA4,SAR1B,SARS1,
SBF2,SCAPER,SCA
RB2,SCYL1,SDC4,S
EC63,SECISBP2L,SE
NP6,SERPINA6,SER
PINE2,SERPINH1,SE
SN3,SETDB2,SFRP5,
SGK2,SGTB,SH3D19
,SHMT1,SHPRH,SIR
T5,SLC10A1,SLC1A4
,SLC20A1,SLC20A2,
SLC27A2,SLC2A2,SL
C2A5,SLC2A9,SLC35
B3,SLC38A2,SLC39A
10,SLC39A8,SLC6A1
2,SLC9A3R1,SLK,SM
AD7,SMAP1,SNAPC2
,SNRNP200,SNTB1,S
NX10,SNX9,SOAT2,S
OD2,SPARC,SPG21,
SPON2,SPOP,SPTB
N1,SPTBN2,ST3GAL
4,STARD7,STIP1,ST
K24,STK40,STOM,ST
T3B,STX8,STXBP3,S
ULF2,SUMF1,SYAP1,
SYNE1,SYT1,TBC1D
16,TBC1D20,TBC1D2
4,TBRG4,TCIM,TCP1
,TEP1,TEX2,THAP4,
THRSP,TIGD2,TIMM

					44,TK1,TLE1,TMBIM1,TMEM126A,TMEM219,TMEM41A,TMEM41B,TMEM62,TMEM97,TNFAIP8L1,TNRC6A,TOP1MT,TPI1,TPR,TPRKB,TRIB3,TRMT1,TSN,TSPAN33,TTC1,TTC19,TUBB,TUBB6,TUBGCP2,TXNL1,UBAC1,UBE2L3,UBR1,UBTF,UBXN1,UBXN6,UCK1,UGP2,UGT2B10,ULK1,UNG,UPP2,UROC1,UROD,USP47,UTP14A,UTRN,UXS1,VPS13A,WDR18,WDR26,WDR81,WIPI2,WNT5B,YWHAH,ZCCHC24,ZFAND2B,ZNF23,ZNF32,ZNF326,ZNF654	
Cancer,Organismal Injury And Abnormalities	Urogenital Cancer	Urogenital Cancer	6.45E-12	-2.015	ABCB1,ABCB4,ABCG2,ABCG5,ACAD8,ACLY,ACOX2,ACSL3,ACSL4,ACSM1,ACSS2,AFG3L2,AGPAT2,AHNAK,AKAP11,AKAP9,ALAS1,ALDH1L1,ALG13,ANKRD12,ARHGAP18,ARHGAP21,ARHGAP6,ARIH2,ARL15,ASAP2,ATP1A1,ATP1B1,ATP6V1D,ATXN2L,AUH,BAAT,BAP1,BDH1,BIK,BLMH,BRD8,CA3,CAPN2,CARS1,CBLB,CCT3,CCT5,CCT6A,CD2AP,CD47,CD9,CD99L2,CDA,CDC14B,CDH1,CDH2,CDO1,CEBPD,CES1,CGN,CHD6,CLDND1,CLK2,CLMN,CMTM8,CNOT4,CNOT6,CNP,COL27A1,COL5A3,CPEB4,CPT1A,CROT,CS,CSRNP1,CTSH,CXADR,CYLD,CYP2U1,CYP4F2,CYP7A1,CYTH1,DAZAP1,DCTN1,DDC,DDX60,DGKZ,DHX9,DIAPH2,DICER1,DIO1,DIPK2A,DIXDC1,DMD,DOCK4,DPP4,DPP9,DSP,DSTN,DUSP19,E2F5,EFHD2,EGR1,EIF1AX,EIF4EBP1,ELF1,ELOVL6,ENPP1,ERBB3,ESRRA,EXOC6,EXOSC2,EXTL2,F11,F2R,	390

F3,FADS2,FAM160B
1,FAM172A,FAM89A,
FECH,FGA,FGFR2,F
GFRL1,FGG,FH,FKB
P4,FLCN,FOXK1,FO
XO3,FTSJ3,G6PD,G
ABARAP,GDA,GNA1
2,GOLGB1,GPD2,GR
HPR,GSDMD,GSK3A
,GSTM2,GTF2A2,GT
F3C2,GYG1,HECA,H
ECTD3,HLF,HMGCR,
HRAS,HSD3B7,HSP
B1,ICK,ID2,IGFBP2,I
L18,ING4,IRF6,IRS1,I
TPA,JAK2,JMJD1C,K
AT2B,KCMF1,KDM6A
,KEAP1,KIAA0100,KL
F9,KLHDC2,KLHL7,K
TN1,KYNU,L2HGDH,
LARS2,LGALS4,LGR
5,LIN7C,LITAF,LMNA
,LPIN1,LRIG1,LRP12,
LRRC28,LSM14B,LS
S,MACROH2A1,MAG
ED1,MAGI3,MAP1LC
3B,MAP2K3,MBNL2,
MCOLN1,MEF2A,MIA
2,MKNK2,MLLT10,M
MD,MOCOS,MON2,M
PDZ,MPP1,MPRIP,M
RPL24,MRPL34,MRP
L44,MRPS22,MRPS2
5,MRPS27,MT1F,MY
O1B,MYO5B,MYO6,N
4BP2,N4BP2L1,NAG
K,NCOA1,NDUFA9,N
DUFB9,NEK7,NFKBI
A,NGEF,NHLRC2,NI
NJ1,NIPBL,NME1,NO
SIP,NPC1,NR2F6,NR
3C2,NR5A2,NRIP1,N
UDT7,NUP62,NUP88,
NUS1,OAZ1,OSBPL1
A,OTUD6B,OXR1,PA
LMD,PAN3,PCDH1,P
CGF5,PDCD2,PDE2A
,PDGFRB,PDLIM1,P
DP2,PDPK1,PDRG1,
PER1,PER3,PEX6,P
GAP1,PHF20L1,PHK
A2,PIK3CA,PKHD1,P
LD1,PLXNA2,PNKD,
PNRC1,POR,PPIF,P
PP1R1B,PPP1R3B,P
PP1R9A,PPP2R1A,P
RPF39,PRPF6,PSEN
2,PSMD1,PSMD2,PT
BP1,PTBP2,PXMP4,
RAB11FIP2,RABEPK,
RABGAP1L,RAI14,R
ALGPS2,RASA3,RB1

					CC1,RBBP5,RBBP7,RCAN1,RCL1,REPIN1,RGS2,RHOD,RICTOR,RNF14,RNF144A,ROCK1,RPIA,SBF2,SCAPER,SCARB2,SCYL1,SDC4,SDR42E1,SEC63,SECISBP2L,SENP6,SERPINA6,SERPINE2,SESN3,SETDB2,SFRP5,SHPRH,SIRT5,SLC16A5,SLC1A4,SLC20A1,SLC20A2,SLC25A32,SLC2A2,SLC2A9,SLC38A2,SLC39A10,SLC39A8,SLC6A12,SLC9A3R1,SLK,SMAD7,SMAP1,SNRNP200,SNTB1,SNX10,SOD2,SPARC,SPG21,SPOP,SPTBN1,SPTBN2,ST3GAL4,STARD7,STK24,STK40,STOM,STT3B,STX8,STXBP3,SULF2,SYNE1,TBC1D16,TBC1D20,TBC1D24,TCP1,TEP1,TEX2,THRSP,TIFA,TIGD2,TIMM44,TK1,TLE1,TMEM126A,TMEM51,TMEM97,TNFAIP2,TNRC6A,TOP1MT,TPI1,TPR,TPRG1L,TRIB3,TRMT1,TSPAN33,TTC1,TUBB,TUBB6,TXNL1,UBAC1,UBE2F,UBR1,UGP2,UGT2B10,UROC1,UROD,USP47,UTP14A,UTRN,UXS1,VPS13A,WIPI2,WNT5B,YPEL3,YWHAH,ZCCHC24,ZFAND2B,ZKSCAN1,ZNF23,ZNF32,ZNF654	
Cancer,Organismal Injury And Abnormalities	Malignant Genitourinary Solid Tumor	Malignant Genitourinary Solid Tumor	1.07E-11	-2.015	ABCB1,ABCB4,ABCG2,ABCG5,ACAD8,ACLY,ACOX2,ACSL3,ACSL4,ACSM1,ACSS2,AFG3L2,AGPAT2,AHNAK,AKAP11,AKAP9,ALAS1,ALDH1L1,ALG13,ANKRD12,ARHGAP18,ARHGAP21,ARHGAP6,ARIH2,ARL15,ASAP2,ATP1A1,ATP1B1,ATP6V1D,ATXN2L,AUH,BAAT,BAP1,BDH1,BIK,BRD8,CA3,CAPN2,CARS1,CBLB,CCT3,CCT5,CCT6A,CD2AP,CD47,	389

CD9,CD99L2,CDA,C
DC14B,CDH1,CDH2,
CDO1,CEBPD,CES1,
CGN,CHD6,CLDND1,
CLK2,CLMN,CMTM8,
CNOT4,CNOT6,CNP,
COL27A1,COL5A3,C
PEB4,CPT1A,CROT,
CS,CSRNP1,CTSH,C
XADR,CYLD,CYP2U1
,CYP4F2,CYP7A1,CY
TH1,DAZAP1,DCTN1
,DDC,DDX60,DGKZ,
DHX9,DIAPH2,DICE
R1,DIO1,DIPK2A,DIX
DC1,DMD,DOCK4,D
PP4,DPP9,DSP,DST
N,DUSP19,E2F5,EFH
D2,EGR1,EIF1AX,EIF
4EBP1,ELF1,ELOVL6
,ENPP1,ERBB3,ESR
RA,EXOC6,EXOSC2,
EXTL2,F11,F2R,F3,F
ADS2,FAM160B1,FA
M172A,FAM89A,FEC
H,FGA,FGFR2,FGFR
L1,FGG,FH,FKBP4,F
LCN,FOXK1,FOXO3,
FTSJ3,G6PD,GABAR
AP,GDA,GNA12,GOL
GB1,GPD2,GRHPR,
GSDMD,GSK3A,GST
M2,GTF2A2,GTF3C2,
GYG1,HECA,HECTD
3,HLF,HMGCR,HRAS
,HSD3B7,HSPB1,ICK
,ID2,IGFBP2,IL18,IN
G4,IRF6,IRS1,ITPA,J
AK2,JMJD1C,KAT2B,
KCMF1,KDM6A,KEA
P1,KIAA0100,KLF9,K
LHDC2,KLHL7,KTN1,
KYNU,L2HGDH,LAR
S2,LGALS4,LGR5,LI
N7C,LITAF,LMNA,LPI
N1,LRIG1,LRP12,LR
RC28,LSM14B,LSS,
MACROH2A1,MAGE
D1,MAGI3,MAP1LC3
B,MAP2K3,MBNL2,M
COLN1,MEF2A,MIA2,
MKNK2,MLLT10,MM
D,MOCOS,MON2,MP
DZ,MPP1,MPRIP,MR
PL24,MRPL34,MRPL
44,MRPS22,MRPS25
,MRPS27,MT1F,MYO
1B,MYO5B,MYO6,N4
BP2,N4BP2L1,NAGK,
NCOA1,NDUFA9,ND
UFB9,NEK7,NFKBIA,
NGEF,NHLRC2,NINJ

1,NIPBL,NME1,NOSI
P,NPC1,NR2F6,NR3
C2,NR5A2,NRIP1,NU
DT7,NUP62,NUP88,N
US1,OAZ1,OSBPL1A
,OTUD6B,OXR1,PAL
MD,PAN3,PCDH1,PC
GF5,PDCD2,PDE2A,
PDGFRB,PDLIM1,PD
P2,PDPK1,PDRG1,P
ER1,PER3,PEX6,PG
AP1,PHF20L1,PHKA
2,PIK3CA,PKHD1,PL
D1,PLXNA2,PNKD,P
NRC1,POR,PPIF,PP
P1R1B,PPP1R3B,PP
P1R9A,PPP2R1A,PR
PF39,PRPF6,PSEN2,
PSMD1,PSMD2,PTB
P1,PTBP2,PXMP4,R
AB11FIP2,RABEPK,R
ABGAP1L,RAI14,RAL
GPS2,RASA3,RB1CC
1,RBBP5,RBBP7,RC
AN1,RCL1,REPIN1,R
GS2,RHOD,RICTOR,
RNF14,RNF144A,RO
CK1,RPIA,SBF2,SCA
PER,SCARB2,SCYL1
,SDC4,SDR42E1,SE
C63,SECISBP2L,SEN
P6,SERPINA6,SERPI
NE2,SESN3,SETDB2
,SFRP5,SHPRH,SIRT
5,SLC16A5,SLC1A4,
SLC20A1,SLC20A2,S
LC25A32,SLC2A2,SL
C2A9,SLC38A2,SLC3
9A10,SLC39A8,SLC6
A12,SLC9A3R1,SLK,
SMAD7,SMAP1,SNR
NP200,SNTB1,SNX1
0,SOD2,SPARC,SPG
21,SPOP,SPTBN1,S
PTBN2,ST3GAL4,ST
ARD7,STK24,STK40,
STOM,STT3B,STX8,
STXBP3,SULF2,SYN
E1,TBC1D16,TBC1D
20,TBC1D24,TCP1,T
EP1,TEX2,THRSP,TI
FA,TIGD2,TIMM44,T
K1,TLE1,TMEM126A,
TMEM51,TMEM97,T
NFAIP2,TNRC6A,TO
P1MT,TPI1,TPR,TPR
G1L,TRIB3,TRMT1,T
SPAN33,TTC1,TUBB,
TUBB6,TXNL1,UBAC
1,UBE2F,UBR1,UGP
2,UGT2B10,UROC1,
UROD,USP47,UTP14

					A,UTRN,UXS1,VPS13A,WIPI2,WNT5B,YPEL3,YWHAH,ZCCHC24,ZFAND2B,ZKSCAN1,ZNF23,ZNF32,ZNF654	
Cancer,Organismal Injury And Abnormalities	Abdominal Neoplasm	Abdominal Neoplasm	1.91E-24	-2.159	AASDH,ABCB1,ABCB4,ABCG2,ABCG5,ABCG8,ABTB2,ACAD8,ACAT2,ACLY,ACOX2,ACSL3,ACSL4,ACSL5,ACSM1,ACSM5,ACSS2,ACTR6,ADH4,AFG3L2,AGPAT2,AHCY,AHNAK,AHSA1,AKAP11,AKAP9,AKR1D1,ALAS1,ALDH1L1,ALG13,AMDHD1,ANKRD12,ANP32A,ANXA7,ARF3,ARGLU1,ARHGAP18,ARHGAP21,ARHGAP6,ARIH2,ARL15,ARL6IP4,ARSG,ASAP2,ASB13,ATG3,ATG4D,ATP1A1,ATP1B1,ATP6V1D,ATXN2L,AUH,BAAT,BAP1,BDH1,BHMT,BHMT2,BID,BIK,BLMH,BRD8,BUD23,C11orf54,C9orf152,C9orf16,CA3,CAMKK2,CAPN2,CARS1,CBLB,CCBE1,CCDC66,CCT3,CCT4,CCT5,CCT6A,CCT7,CD2AP,CD47,CD9,CD99L2,CDA,CDC14B,CDH1,CDH2,CDO1,CEBPD,CENPV,CES1,CGN,CHCHD3,CHCHD4,CHD6,CHPT1,CHRAC1,CIB3,CITED2,CLDND1,CLK2,CLMN,CLOCK,CMTM6,CMTM8,CNOT4,CNOT6,CNP,COL27A1,COL5A3,CORO1B,CPEB4,CPT1A,CRIP2,CROT,CS,CSRNP1,CTDSP1,CTPS2,CTSH,CUL4B,CXADR,CYLD,CYP2U1,CYP39A1,CYP4F2,CYP7A1,CYTH1,DAZAP1,DCTN1,DDC,DDX60,DEK,DGKZ,DHX9,DIAPH2,DICER1,DIO1,DIPK2A,DIXDC1,DMD,DMTF1,DNAJC5,DOCK4,DPP4,DPP9,DSP,DSTN,DUSP19,DVL1,E2F5,ECHDC1,ECHDC3,EEPD1,	558

EFHD2,EGR1,EIF1A
X,EIF4EBP1,ELF1,E
NPP1,ERBB3,ESRRA
,EXOC6,EXOSC2,EX
TL2,F11,F2R,F3,FAD
S2,FAM102A,FAM16
0B1,FAM172A,FAM8
9A,FBXO9,FECH,FG
A,FGB,FGFR2,FGFR
L1,FGG,FH,FKBP4,F
LCN,FMO1,FMO5,FO
XK1,FOXO3,FPGS,F
TSJ3,G6PD,GABARA
P,GAS2L3,GCAT,GC
LC,GCNT2,GDA,GNA
12,GNG12,GOLGB1,
GPD2,GPN1,GRHPR,
GSDMD,GSK3A,GSS
,GSTM2,GTF2A2,GT
F2F1,GTF3C2,GYG1,
HECA,HECTD3,HER
C4,HLF,HMG20B,HM
GCR,HNRNPC,HNR
NPD,HOMER2,HOOK
1,HRAS,HSD17B2,H
SD3B7,HSPB1,ICK,I
D2,ID4,IGFBP2,IL18,I
NG4,IRF6,IRS1,ITPA,
ITPK1,JAK2,JMJD1C,
KAT2B,KCMF1,KDM6
A,KEAP1,KIAA0100,K
ITLG,KLF12,KLF9,KL
HDC2,KLHL21,KLHL
7,KPNB1,KTN1,KYN
U,L2HGDH,LARS2,L
GALS4,LGR5,LIN7A,
LIN7C,LITAF,LMNA,L
PIN1,LRIG1,LRP12,L
RRC28,LRRC3,LSM1
4B,LSS,LTBR,MACR
OH2A1,MAGED1,MA
GI3,MAP1LC3B,MAP
2K3,MAP3K7,MBNL2,
MCOLN1,MEF2A,MIA
2,MKNK2,MLLT10,ML
X,MMD,MOCOS,MO
N2,MPDZ,MPP1,MPR
IP,MRNIP,MRPL12,M
RPL20,MRPL24,MRP
L44,MRPS22,MRPS2
5,MRPS27,MRPS9,M
T1F,MYO1B,MYO5B,
MYO6,N4BP2,N4BP2
L1,NAGK,NCALD,NC
OA1,NDUFA12,NDUF
A9,NEK7,NET1,NFKB
IA,NGEF,NHLRC2,NI
NJ1,NIPBL,NME1,NO
SIP,NPC1,NR1H4,NR
2F6,NR3C2,NR5A2,N
RIP1,NT5C,NUDT1,N
UDT19,NUDT7,NUP6

2,NUP88,OAZ1,OSB
PL1A,OTUD6B,OXR1
,PALMD,PAN2,PAN3,
PAQR9,PCDH1,PCG
F5,PDCD2,PDE2A,P
DE9A,PDGFC,PDGF
RB,PDLIM1,PDP2,PD
PK1,PDRG1,PER1,P
ER3,PEX26,PEX6,PF
DN2,PGAP1,PHF20L
1,PHKA2,PIK3AP1,PI
K3CA,PKHD1,PLD1,
PLXNA2,PMPCA,PN
KD,PNRC1,POLE4,P
OR,PPIF,PPM1A,PP
OX,PPP1R12A,PPP1
R14B,PPP1R1B,PPP
1R3B,PPP1R9A,PPP
2R1A,PRDX6,PRPF3
9,PRPF6,PRPS1,PSE
N2,PSMA6,PSMD1,P
SMD11,PSMD2,PSM
D8,PSMF1,PSPC1,P
SPH,PTBP1,PTBP2,P
XMP4,QDPR,RAB11
FIP2,RABEPK,RABG
AP1L,RAI14,RALGPS
2,RAN,RASA3,RASS
F3,RB1CC1,RBBP5,
RBBP7,RCAN1,RCL1
,REPIN1,RGS2,RHB
DD1,RHOD,RICTOR,
RNF144A,RNF167,R
OCK1,RPA1,RPIA,R
UFY3,SAA4,SAR1B,S
ARS1,SBF2,SCAPER
,SCARB2,SCYL1,SD
C4,SDR42E1,SEC63,
SECISBP2L,SENP6,
SERPINA6,SERPINE
2,SERPINH1,SESN3,
SETDB2,SFRP5,SGK
2,SGTB,SH3D19,SH
MT1,SHPRH,SIRT5,S
LC10A1,SLC16A5,SL
C1A4,SLC20A1,SLC2
0A2,SLC25A32,SLC2
7A2,SLC2A2,SLC2A5
,SLC2A9,SLC30A10,
SLC35B1,SLC35B3,S
LC38A2,SLC39A10,S
LC39A8,SLC6A12,SL
C9A3R1,SLK,SMAD7
,SMAP1,SNAPC2,SN
RNP200,SNTB1,SNX
10,SNX9,SOAT2,SO
D2,SPARC,SPG21,S
PON2,SPOP,SPTBN
1,SPTBN2,ST3GAL4,
STARD7,STIP1,STK2
4,STK40,STOM,STT3
B,STX8,STXBP3,SUL

					F2,SUMF1,SYAP1,SYNE1,SYT1,TBC1D16,TBC1D20,TBC1D24,TBRG4,TCIM,TCP1,TEP1,TEX2,THAP4,THRSP,TIGD2,TIMM44,TK1,TLCD4,TLE1,TMBIM1,TMEM126A,TMEM219,TMEM41A,TMEM41B,TMEM51,TMEM62,TMEM97,TNFAIP2,TNFAIP8L1,TNRC6A,TOP1MT,TPI1,TPR,TPRG1L,TPRKB,TRIB3,TRMT1,TSN,TSPAN33,TTC1,TTC19,TUBB,TUBB6,TUBGCP2,TXNL1,UBAC1,UBE2F,UBE2L3,UBR1,UBTF,UBXN1,UBXN6,UCK1,UGP2,UGT2B10,ULK1,UNG,UPP2,UROC1,UROD,USP47,UTP14A,UTRN,UXS1,VPS13A,WDR18,WDR26,WDR81,WIPI2,WNT5B,YPEL3,YWHAH,ZCCHC24,ZFAND2B,ZKSCAN1,ZNF23,ZNF32,ZNF326,ZNF654	
Molecular Transport	Transport	Transport Of Molecule	3.02E-12	-2.164	ABCB1,ABCB4,ABCG2,ABCG5,ABCG8,ACAT2,ACLY,ACSL3,ACSL4,ACSL5,AFG3L2,ANP32A,AQP11,ARF3,ATP1A1,ATP1B1,ATP6V1D,BET1,CA3,CBLB,CD2AP,CDH1,CES1,CHCHD4,CITED2,CMTM6,CNP,CPT1A,CROT,CYP7A1,CYTH1,DCTN1,DDC,DGKZ,DNAJC5,DVL1,EGR1,ENPP1,ERBB3,F2R,FECH,FGA,FGB,FGG,FH,FKBP4,FOXO3,GABARAP,GSK3A,HMGCR,HNRNPD,HOOK1,HRAS,HSD17B2,IRS1,JAK2,KPNB1,LIN7A,LIN7C,LMNA,LSS,MAP2K3,MEF2A,MLX,MYO5B,MYO6,NFKBIA,NPC1,NR1H4,NR3C2,NR5A2,NUP62,NUP88,OGA,OSBPL1A,PDGFRB,PDPK1,PER1,PEX26,PEX6,PGAP1,PIK3CA,PMPCA,PNKD,PPP1R3B,PRDX6,PSEN2,R	129

					AN,REPIN1,RHBDD1,RICTOR,ROCK1,SCARB2,SERPINA6,SGK2,SLC10A1,SLC1A4,SLC20A1,SLC20A2,SLC27A2,SLC2A2,SLC2A5,SLC2A9,SLC30A10,SLC35A3,SLC38A2,SLC38A3,SLC39A10,SLC39A8,SLC6A12,SLC9A3R1,SNTB1,SNX10,SNX9,SOAT2,SOD2,SPTBN2,STARD4,STX18,STX7,STX8,STXBP3,SYT1,TIMM44,TPR,TRIB3,UROD,VPS11,YWHAH	
Cancer,Organismal Injury And Abnormalities	Abdominal Cancer	Abdominal Cancer	1,60E-23	-2,169	AASDH,ABCB1,ABCB4,ABCG2,ABCG5,ABCG8,ABTB2,ACAD8,ACAT2,ACLY,ACOX2,ACSL3,ACSL4,ACSL5,ACSM1,ACSM5,ACSS2,ACTR6,ADH4,AFG3L2,AGPAT2,AHCY,AHNAK,AHSA1,AKAP11,AKAP9,AKR1D1,ALAS1,ALDH1L1,ALG13,AMDHD1,ANKRD12,ANP32A,ANXA7,ARF3,ARGLU1,ARHGAP18,ARHGAP21,ARHGAP6,ARIH2,ARL15,ARL6IP4,ARSG,ASAP2,ASB13,ATG3,ATG4D,ATP1A1,ATP1B1,ATP6V1D,ATXN2L,AUH,BAAT,BAP1,BDH1,BHMT,BHMT2,BID,BIK,BLMH,BRD8,BUD23,C11orf54,C9orf152,CA3,CAMKK2,CAPN2,CARS1,CBLB,CCBE1,CCDC66,CCT3,CCT4,CCT5,CCT6A,CCT7,CD2AP,CD47,CD9,CD99L2,CDA,CDC14B,CDH1,CDH2,CDO1,CEBPD,CENPV,CES1,CGN,CHCHD3,CHCHD4,CHD6,CHPT1,CHRAC1,CIB3,CITED2,CLDND1,CLK2,CLMN,CLOCK,CMTM6,CMTM8,CNOT4,CNOT6,CNP,COL27A1,COL5A3,CORO1B,CPEB4,CPT1A,CRIP2,CROT,CS,CSRNP1,CTDSP1,CTPS2,CTSH,CUL4B,CXADR,CYLD,CYP2U1,CYP39	551

A1,CYP4F2,CYP7A1,
CYTH1,DAZAP1,DCT
N1,DDC,DDX60,DEK,
DGKZ,DHX9,DIAPH2,
DICER1,DIO1,DIPK2
A,DIXDC1,DMD,DMT
F1,DNAJC5,DOCK4,
DPP4,DPP9,DSP,DS
TN,DUSP19,DVL1,E2
F5,ECHDC1,EEPD1,
EFHD2,EGR1,EIF1A
X,EIF4EBP1,ELF1,E
NPP1,ERBB3,ESRRA
,EXOC6,EXOSC2,EX
TL2,F11,F2R,F3,FAD
S2,FAM102A,FAM16
0B1,FAM172A,FAM8
9A,FBXO9,FECH,FG
A,FGB,FGFR2,FGFR
L1,FGG,FH,FKBP4,F
LCN,FMO1,FMO5,FO
XK1,FOXO3,FPGS,F
TSJ3,G6PD,GABARA
P,GAS2L3,GCAT,GC
LC,GCNT2,GDA,GNA
12,GNG12,GOLGB1,
GPD2,GPN1,GRHPR,
GSDMD,GSK3A,GSS
,GSTM2,GTF2A2,GT
F2F1,GTF3C2,GYG1,
HECA,HECTD3,HER
C4,HLF,HMG20B,HM
GCR,HNRNPC,HNR
NPD,HOMER2,HOOK
1,HRAS,HSD17B2,H
SD3B7,HSPB1,ICK,I
D2,ID4,IGFBP2,IL18,I
NG4,IRF6,IRS1,ITPA,
ITPK1,JAK2,JMJD1C,
KAT2B,KCMF1,KDM6
A,KEAP1,KIAA0100,K
ITLG,KLF12,KLF9,KL
HDC2,KLHL21,KLHL
7,KPNB1,KTN1,KYN
U,L2HGDH,LARS2,L
GALS4,LGR5,LIN7A,
LIN7C,LITAF,LMNA,L
PIN1,LRIG1,LRP12,L
RRC28,LRRC3,LSM1
4B,LSS,LTBR,MACR
OH2A1,MAGED1,MA
GI3,MAP1LC3B,MAP
2K3,MAP3K7,MBNL2,
MCOLN1,MEF2A,MIA
2,MKNK2,MLLT10,ML
X,MMD,MOCOS,MO
N2,MPDZ,MPP1,MPR
IP,MRNIP,MRPL12,M
RPL20,MRPL24,MRP
L44,MRPS22,MRPS2
5,MRPS27,MRPS9,M
T1F,MYO1B,MYO5B,

MYO6,N4BP2,N4BP2
L1,NAGK,NCALD,NC
OA1,NDUFA12,NDUF
A9,NEK7,NET1,NFKB
IA,NGEF,NHLRC2,NI
NJ1,NIPBL,NME1,NO
SIP,NPC1,NR1H4,NR
2F6,NR3C2,NR5A2,N
RIP1,NT5C,NUDT1,N
UDT19,NUDT7,NUP6
2,NUP88,OAZ1,OSB
PL1A,OTUD6B,OXR1
,PALMD,PAN2,PAN3,
PAQR9,PCDH1,PCG
F5,PDCD2,PDE2A,P
DE9A,PDGFC,PDGF
RB,PDLIM1,PDP2,PD
PK1,PDRG1,PER1,P
ER3,PEX26,PEX6,PF
DN2,PGAP1,PHF20L
1,PHKA2,PIK3AP1,PI
K3CA,PKHD1,PLD1,
PLXNA2,PMPCA,PN
KD,PNRC1,POLE4,P
OR,PPIF,PPM1A,PP
OX,PPP1R12A,PPP1
R14B,PPP1R1B,PPP
1R3B,PPP1R9A,PPP
2R1A,PRPF39,PRPF
6,PRPS1,PSEN2,PS
MD1,PSMD11,PSMD
2,PSMD8,PSMF1,PS
PC1,PSPH,PTBP1,P
TBP2,PXMP4,QDPR,
RAB11FIP2,RABEPK,
RABGAP1L,RAI14,R
ALGPS2,RAN,RASA3
,RASSF3,RB1CC1,R
BBP5,RBBP7,RCAN1
,RCL1,REPIN1,RGS2
,RHBDD1,RHOD,RIC
TOR,RNF144A,RNF1
67,ROCK1,RPA1,RPI
A,RUFY3,SAA4,SAR
1B,SARS1,SBF2,SCA
PER,SCARB2,SCYL1
,SDC4,SDR42E1,SE
C63,SECISBP2L,SEN
P6,SERPINA6,SERPI
NE2,SERPINH1,SES
N3,SETDB2,SFRP5,S
GK2,SGTB,SH3D19,
SHMT1,SHPRH,SIRT
5,SLC10A1,SLC16A5
,SLC1A4,SLC20A1,S
LC20A2,SLC25A32,S
LC27A2,SLC2A2,SLC
2A5,SLC2A9,SLC35B
3,SLC38A2,SLC39A1
0,SLC39A8,SLC6A12
,SLC9A3R1,SLK,SMA
D7,SMAP1,SNAPC2,

					SNRNP200,SNTB1,SNX10,SNX9,SOAT2,SOD2,SPARC,SPG21,SPON2,SPOP,SPTBN1,SPTBN2,ST3GAL4,STARD7,STIP1,STK24,STK40,STOM,ST T3B,STX8,STXBP3,SULF2,SUMF1,SYAP1,SYNE1,SYT1,TBC1D16,TBC1D20,TBC1D24,TBRG4,TCIM,TCP1,TEP1,TEX2,THAP4,THRSP,TIGD2,TIMM44,TK1,TLE1,TMBIM1,TMEM126A,TMEM219,TMEM41A,TMEM41B,TMEM51,TMEM62,TMEM97,TNFAIP2,TNFAIP8L1,TNRC6A,TOP1MT,TPI1,TPR,TPRG1L,TPRKB,TRIB3,TRMT1,TSN,TSPAN33,TTC1,TTC19,TUBB,TUBB6,TUBGCP2,TXNL1,UBAC1,UBE2F,UBE2L3,UBR1,UBTF,UBXN1,UBXN6,UCK1,UGP2,UGT2B10,ULK1,UNG,UPP2,UROC1,UROD,USP47,UTP14A,UTRN,UXS1,VPS13A,WDR18,WDR26,WDR81,WIPI2,WNT5B,YPEL3,YWHAH,ZCCHC24,ZFAND2B,ZKSCAN1,ZNF23,ZNF32,ZNF326,ZNF654	
Neurological Disease	Motor Dysfunction Or Movement Disorder	Motor Dysfunction Or Movement Disorder	5.12E-08	-2.191	ABCB1,AFG3L2,AHCY,ALAS1,ALG13,ARIH2,ATP1B1,BID,CA3,CAMKK2,CDH2,CDO1,CHD6,CNP,CYTH1,DDC,DGKZ,DICER1,DIXDC1,DNAJB1,DNAJC5,E2F5,EGR1,ENPP1,ERBB3,F3,FGFRL1,FGG,FKBP4,FOXO3,HMGCR,HRAS,HSPB1,ID4,IL18,ITPA,JMJD1C,LGR5,LMNA,MBNL2,MCOLN1,MPDZ,MYO1B,NGEF,NINJ1,NME1,NPC1,NUDT1,NUP62,OXR1,PDCD2,PDLIM1,PDPK1,PFKFB1,PLXNA2,PMPCA,PNKD,PPP1R1B,PRDX6,PTBP2,RAN,RCAN1,RGS2,SARS1,SCARB2,SCOC,SCYL1,SDC4,SERPI	86

					NE2,SGTB,SLC1A4,SLC30A10,SOD2,SPTBN2,STARD4,STOM,SYNE1,SYT1,TBC1D24,TPI1,TRIB3,TSN,UBAC1,VPS13A,WDR26,WDR81	
Neurological Disease	Movement Disorder	Movement Disorders	4.11E-07	-2.37	ABCB1,AFG3L2,AHCY,ALAS1,ALG13,ARIH2,ATP1B1,BID,CA3,CAMKK2,CDH2,CNP,CYTH1,DDC,DGKZ,DICER1,DIXDC1,DNAJB1,DNAJC5,E2F5,EGR1,ERBB3,F3,FGFRL1,FGG,FKBP4,FOXO3,HMGCR,HRAS,HSPB1,ID4,IL18,ITPA,JMJD1C,LGR5,LMNA,MBNL2,MCOLN1,MPDZ,MYO1B,NGEF,NINJ1,NME1,NPC1,NUDT1,NUP62,OXR1,PDCD2,PDLIM1,PDPK1,PFKFB1,PLXNA2,PMPCA,PNKD,PPP1R1B,PRDX6,PTBP2,RAN,RCAN1,RGS2,SARS1,SCARB2,SCOC,SCYL1,SDC4,SGTB,SLC1A4,SLC30A10,SOD2,SPTBN2,STARD4,STOM,SYNE1,SYT1,TBC1D24,TPI1,TRIB3,TSN,UBAC1,VPS13A,WDR26,WDR81	82
Cancer,Organismal Injury And Abnormalities	Extracranial Solid Tumor	Extracranial Solid Tumor	3.07E-29	-2.464	AASDH,ABCB1,ABCB4,ABCG2,ABCG5,ABCG8,ABTB2,ACAD8,ACAT2,ACLY,ACOX2,ACSL3,ACSL4,ACSL5,ACSM1,ACSM5,ACSS2,ACTR6,ADH4,AFG3L2,AGPAT2,AHCY,AHNAK,AHSA1,AKAP11,AKAP9,AKR1D1,ALAS1,ALDH1L1,ALG13,AMDHD1,ANKRD12,ANP32A,ANXA7,ARF3,ARGLU1,ARHGAP18,ARHGAP21,ARHGAP6,ARIH2,ARL15,ARL4A,ARL6IP4,ARSG,ASAP2,ASB13,ATG3,ATG4D,ATP1A1,ATP1B1,ATP6V1D,ATXN2L,AUH,BAAT,BAP1,BDH1,BHMT,BHMT2,BID,BIK,BLMH,BRD8,BUD23,C11orf54,C9orf152,C9orf16,CA3,CAMKK2,CAP	596

N2,CARS1,CBLB,CC
BE1,CCDC66,CCT3,
CCT4,CCT5,CCT6A,
CCT7,CD2AP,CD47,
CD9,CD99L2,CDA,C
DC14B,CDH1,CDH2,
CDO1,CEBPD,CENP
V,CES1,CGN,CHCH
D3,CHCHD4,CHD6,C
HPT1,CHRAC1,CIB3,
CITED2,CLDND1,CL
K2,CLMN,CLOCK,CL
TA,CMTM6,CMTM8,
CNN3,CNOT4,CNOT
6,CNP,COL27A1,CO
L5A3,CORO1B,CPEB
4,CPT1A,CRIP2,CRL
S1,CROT,CS,CSRNP
1,CTDSP1,CTPS2,CT
SH,CUL4B,CXADR,C
YB5B,CYLD,CYP2U1
,CYP39A1,CYP4F2,C
YP7A1,CYTH1,DAZA
P1,DCTN1,DDC,DDX
60,DEK,DGKZ,DHX9,
DIAPH2,DICER1,DIO
1,DIPK2A,DIXDC1,D
MD,DMTF1,DNAJB1,
DNAJC5,DOCK4,DP
P4,DPP9,DSP,DSTN,
DTYMK,DUSP19,DVL
1,E2F5,ECHDC1,EC
HDC3,EEPD1,EFHD2
,EGR1,EIF1AX,EIF4E
BP1,ELF1,ELOVL6,E
NDOG,ENPP1,ERBB
3,ESRRA,EXOC6,EX
OSC2,EXTL2,F11,F2
R,F3,FADS2,FAM102
A,FAM118B,FAM160
B1,FAM172A,FAM76
B,FAM89A,FBXO31,F
BXO9,FECH,FGA,FG
B,FGFR2,FGFRL1,F
GG,FH,FKBP4,FLCN,
FMO1,FMO5,FOXK1,
FOXO3,FPGS,FTSJ3,
G6PD,GABARAP,GA
S2L3,GCAT,GCLC,G
CNT2,GDA,GNA12,G
NG12,GOLGB1,GPD
2,GPN1,GRHPR,GSD
MD,GSK3A,GSS,GST
M2,GTF2A2,GTF2F1,
GTF3C2,GYG1,HEC
A,HECTD3,HERC4,H
LF,HMG20B,HMGCR,
HNRNPC,HNRNPD,H
OMER2,HOOK1,HRA
S,HSD17B2,HSD3B7,
HSPB1,ICK,ID2,ID4,I
GFBP2,IL18,ING4,IR

F6,IRS1,ITPA,ITPK1,
JAK2,JMJD1C,KAT2
B,KCMF1,KDM6A,KE
AP1,KHK,KIAA0100,
KITLG,KLF12,KLF9,K
LHDC2,KLHL21,KLH
L7,KPNB1,KTN1,KYN
U,L2HGDH,LARS2,L
GALS4,LGR5,LIN7A,
LIN7C,LITAF,LMNA,L
PIN1,LRIG1,LRP12,L
RRC28,LRRC3,LSM1
4B,LSS,LTBR,MACR
OH2A1,MAGED1,MA
GI3,MAP1LC3B,MAP
2K3,MAP3K7,MBNL2,
MCOLN1,MEF2A,MIA
2,MKNK2,MLLT10,ML
X,MMD,MOCOS,MO
N2,MPDZ,MPP1,MPR
IP,MRNIP,MRPL12,M
RPL20,MRPL24,MRP
L34,MRPL44,MRPS2
2,MRPS25,MRPS27,
MRPS9,MT1F,MYO1
B,MYO5B,MYO6,N4B
P2,N4BP2L1,NAGK,N
CALD,NCOA1,NDUF
A12,NDUFA9,NDUFB
9,NEK7,NET1,NFKBI
A,NGEF,NHLRC2,NI
CN1,NINJ1,NIPBL,N
ME1,NOP10,NOSIP,
NPC1,NR1H4,NR2F6
,NR3C2,NR5A2,NRIP
1,NT5C,NUDT1,NUD
T19,NUDT7,NUP62,N
UP88,NUS1,OAZ1,O
SBPL1A,OTUD6B,OX
R1,PALMD,PAN2,PA
N3,PAQR9,PCDH1,P
CGF5,PDCD2,PDE2A
,PDE9A,PDGFC,PDG
FRB,PDLIM1,PDP2,P
DPK1,PDRG1,PER1,
PER3,PES1,PEX26,P
EX6,PFDN2,PFKFB1,
PGAP1,PHF20L1,PH
KA2,PIK3AP1,PIK3C
A,PKHD1,PLD1,PLX
NA2,PMPCA,PNKD,P
NRC1,POLE4,POR,P
PIF,PPM1A,PPOX,PP
P1R12A,PPP1R14B,
PPP1R1B,PPP1R3B,
PPP1R9A,PPP2R1A,
PRDX6,PRPF19,PRP
F39,PRPF6,PRPS1,P
RPS2,PSEN2,PSMA6
,PSMD1,PSMD11,PS
MD2,PSMD8,PSME2,
PSMF1,PSMG1,PSP

C1,PSPH,PTBP1,PT
BP2,PXMP4,QDPR,R
AB11FIP2,RAB1B,RA
BEPK,RABGAP1L,RA
I14,RALGPS2,RAN,R
ASA3,RASSF3,RB1C
C1,RBBP5,RBBP7,R
BKS,RCAN1,RCL1,R
EPIN1,RGS2,RHBDD
1,RHOD,RICTOR,RN
F14,RNF144A,RNF16
7,ROCK1,RPA1,RPIA
,RUFY3,SAA4,SAR1
B,SARS1,SBF2,SCA
PER,SCARB2,SCGN,
SCOC,SCYL1,SDC4,
SDR42E1,SEC63,SE
CISBP2L,SENP6,SE
RPINA6,SERPINE2,S
ERPINH1,SESN3,SE
TDB2,SFRP5,SGK2,
SGTB,SH3BGRL2,SH
3D19,SHMT1,SHPRH
,SIRT5,SLC10A1,SLC
16A5,SLC1A4,SLC20
A1,SLC20A2,SLC25A
32,SLC27A2,SLC2A2
,SLC2A5,SLC2A9,SL
C30A10,SLC35A3,SL
C35B1,SLC35B3,SLC
38A2,SLC39A10,SLC
39A8,SLC6A12,SLC9
A3R1,SLK,SMAD7,S
MAP1,SNAPC2,SNR
NP200,SNTB1,SNX1
0,SNX5,SNX9,SOAT2
,SOD2,SPARC,SPG2
1,SPON2,SPOP,SPT
BN1,SPTBN2,ST3GA
L4,STARD4,STARD7,
STIP1,STK24,STK40,
STOM,STT3B,STX18,
STX8,STXBP3,SULF
2,SUMF1,SYAP1,SY
NE1,SYT1,TBC1D16,
TBC1D20,TBC1D24,
TBRG4,TCIM,TCP1,T
EP1,TEX2,THAP4,TH
RSP,TIFA,TIGD2,TIM
M10,TIMM44,TK1,TL
CD4,TLE1,TMBIM1,T
MEM126A,TMEM219,
TMEM41A,TMEM41B
,TMEM51,TMEM62,T
MEM97,TNFAIP2,TN
FAIP8L1,TNRC6A,TO
P1MT,TPI1,TPR,TPR
G1L,TPRKB,TRIB3,T
RMT1,TSN,TSPAN33
,TSTA3,TTC1,TTC19,
TUBB,TUBB6,TUBG
CP2,TXN2,TXNL1,UB

AC1,UBE2F,UBE2L3,
UBR1,UBTF,UBXN1,
UBXN6,UCK1,UGP2,
UGT2B10,ULK1,UNG
,UPP2,UROC1,UROD
,USP47,UTP14A,UTR
N,UXS1,VPS13A,WD
R18,WDR26,WDR81,
WIPI2,WNT5B,YPEL3
,YWHAH,ZCCHC24,Z
FAND2B,ZKSCAN1,Z
NF23,ZNF32,ZNF326
,ZNF654

An increase in the Z-score value indicates pathway activation, while the decrease in the Z-score indicates pathway inhibition. On a logarithmic scale (-log (p-value) a P-value equal to 0.05 corresponds to 1.3 and the statistical significance of the result increases with the decrease in p-value and with the increase in - log (p-value) values.
